# Supplementary figures and images for: Systematic mapping of mitochondrial calcium uniporter channel (MCUC)-mediated calcium signaling networks
Source: EMBO J. 2024 Sep 11;43(21):22. doi: 10.1038/s44318-024-00219-w (PMC11535509; doi:10.1038/s44318-024-00219-w)

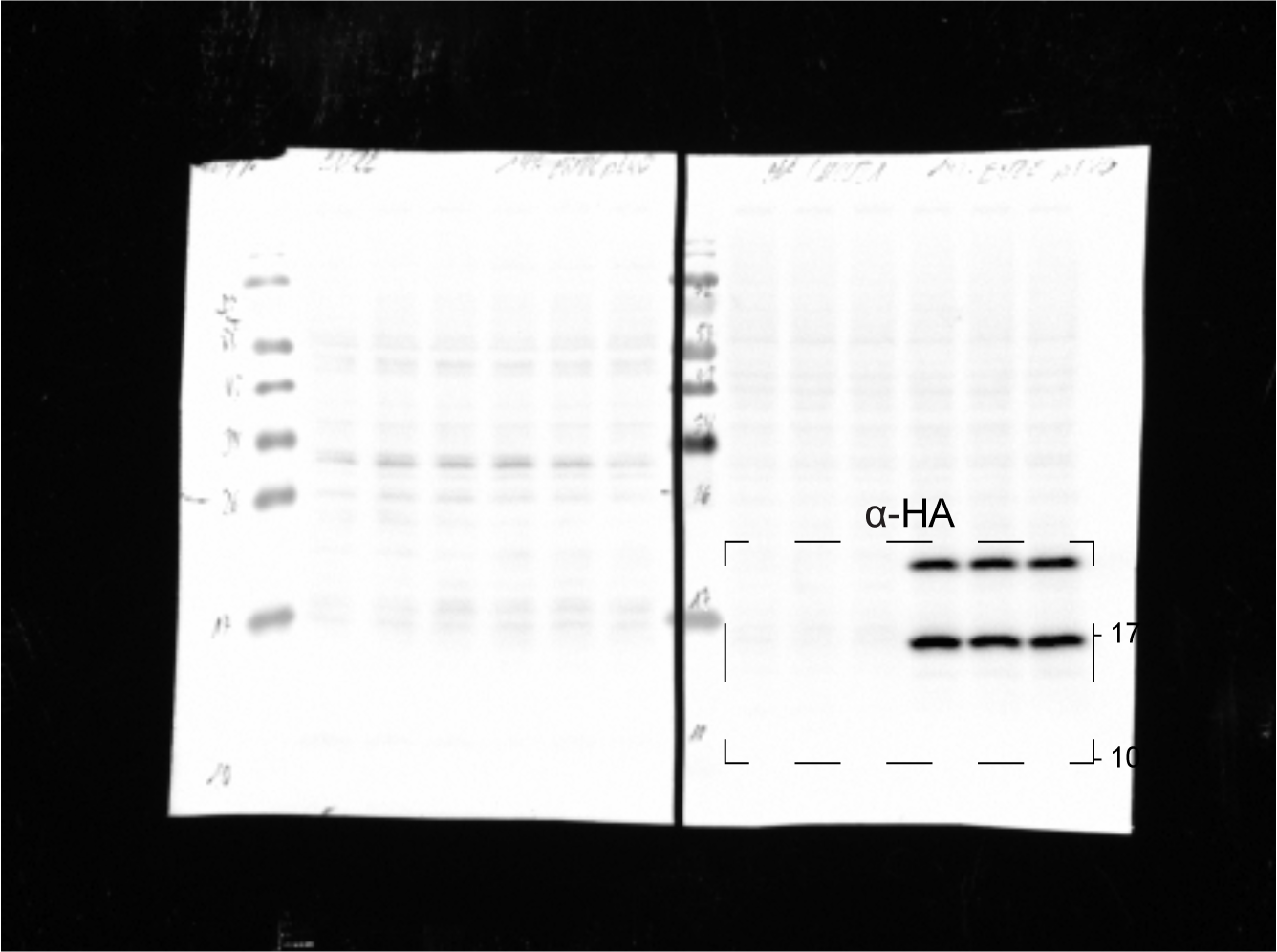

Supplement: Supplementary file 7 — Source data Fig. 2 [file 44318_2024_219_MOESM7_ESM.zip › Figure 2/2A/EMRE-HA_MW.tif]

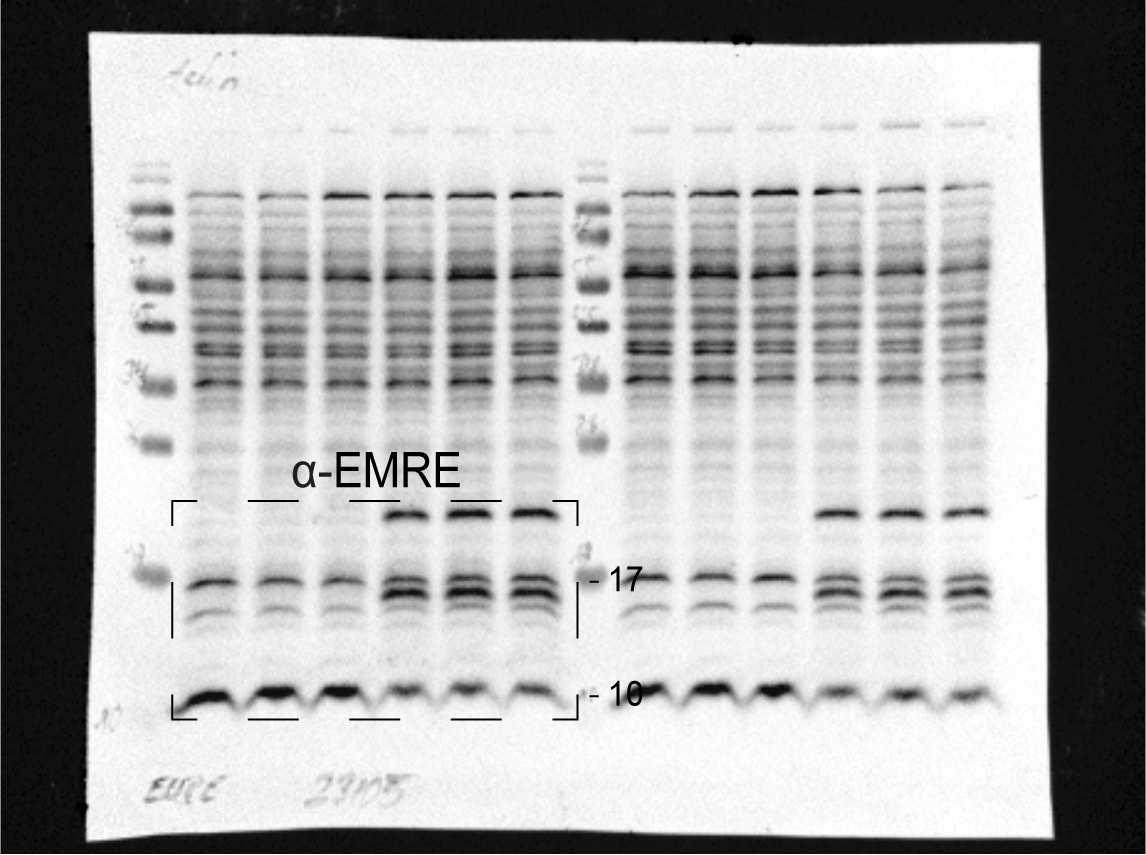

Supplement: Supplementary file 7 — Source data Fig. 2 [file 44318_2024_219_MOESM7_ESM.zip › Figure 2/2A/EMRE_MW.tif]

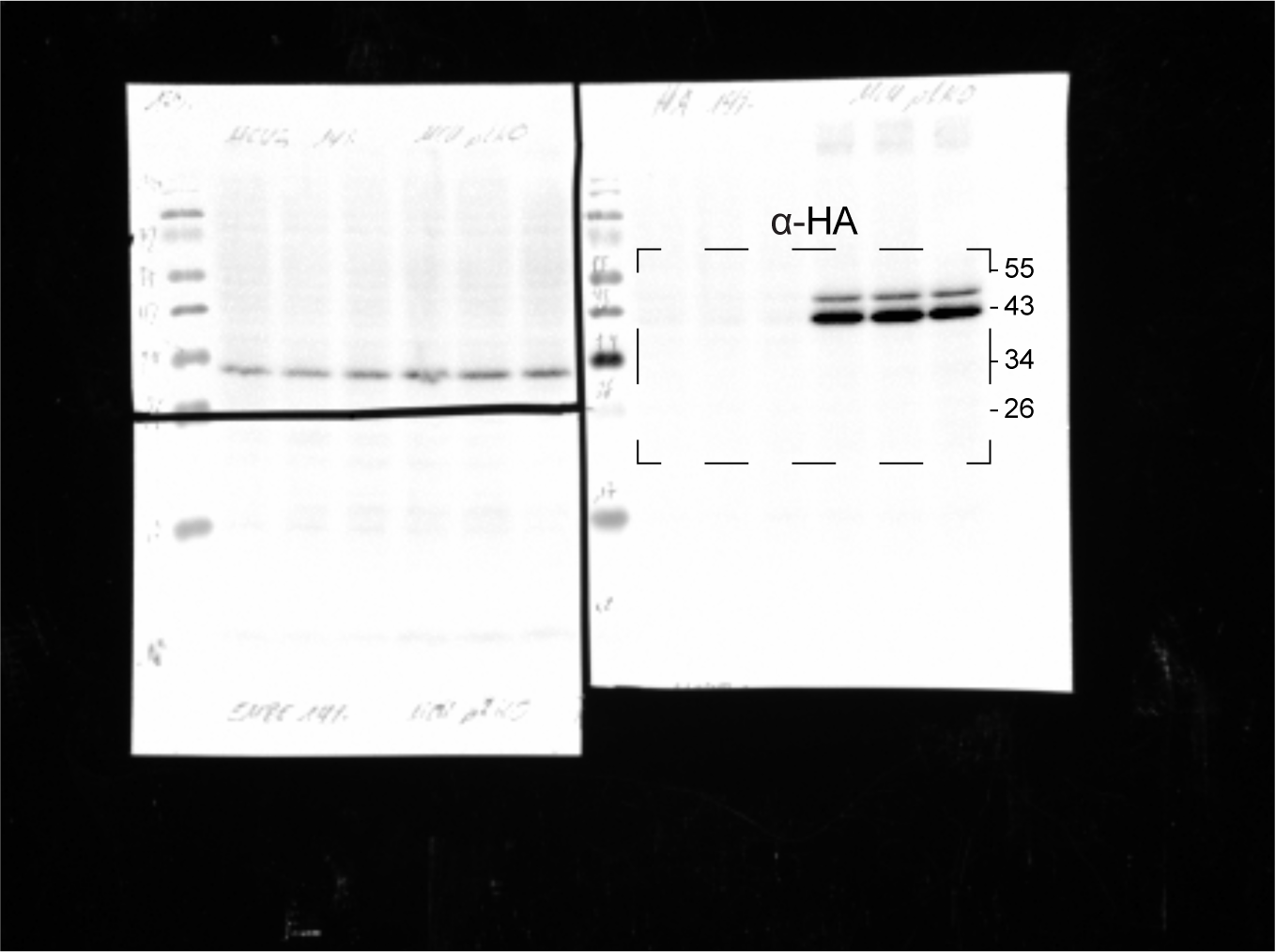

Supplement: Supplementary file 7 — Source data Fig. 2 [file 44318_2024_219_MOESM7_ESM.zip › Figure 2/2A/MCU-HA_MW.tif]

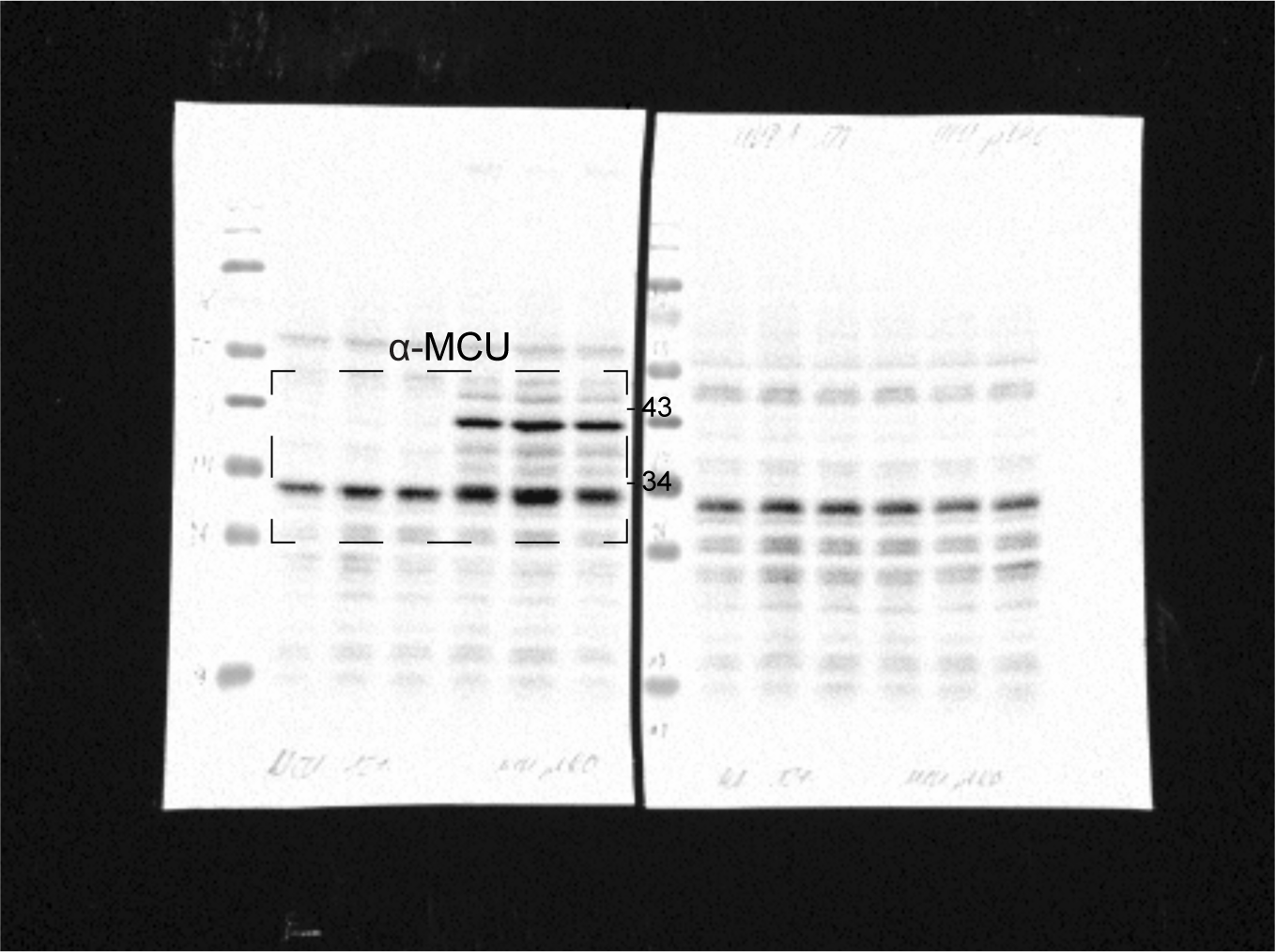

Supplement: Supplementary file 7 — Source data Fig. 2 [file 44318_2024_219_MOESM7_ESM.zip › Figure 2/2A/MCU_MW.tif]

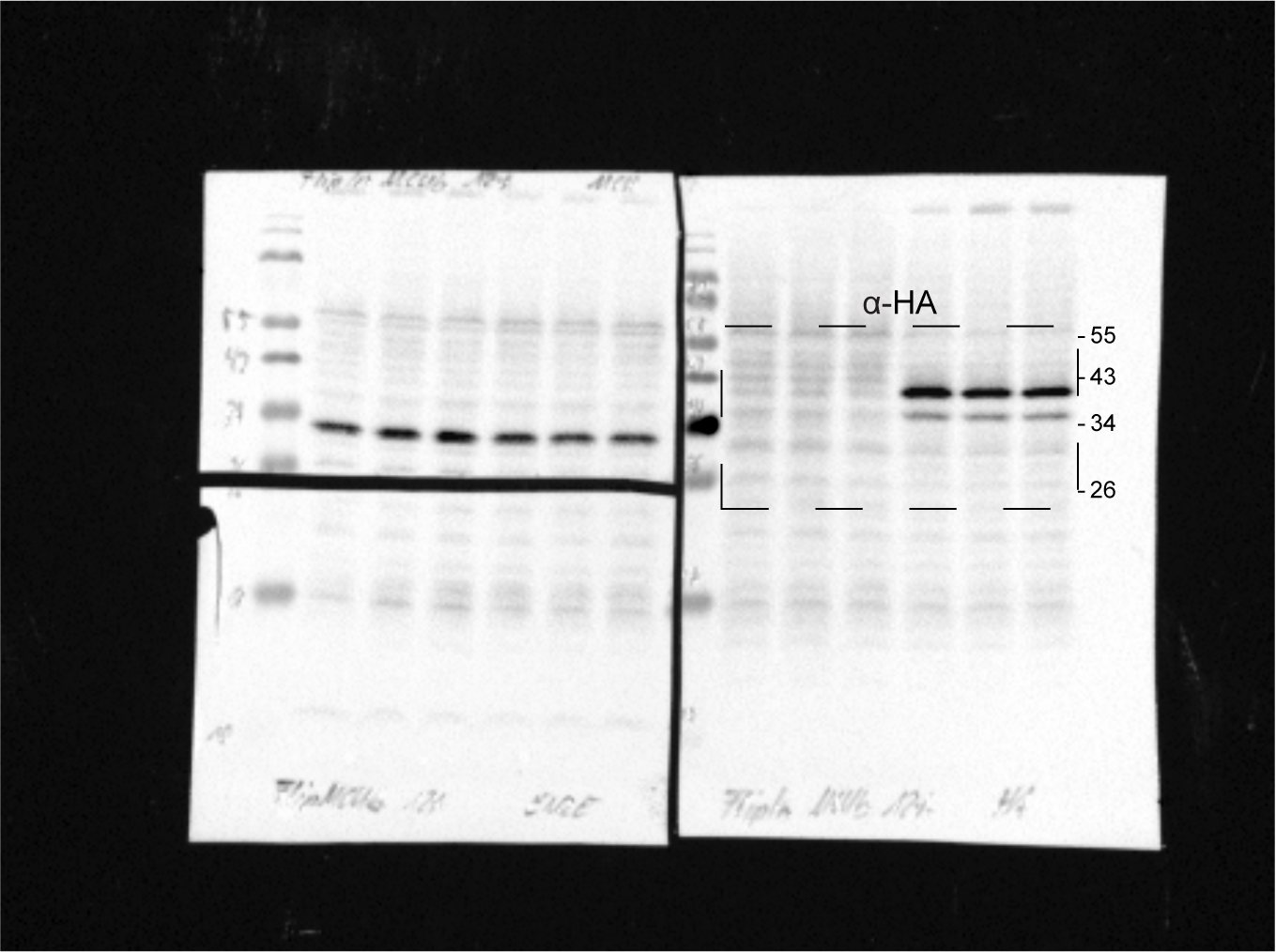

Supplement: Supplementary file 7 — Source data Fig. 2 [file 44318_2024_219_MOESM7_ESM.zip › Figure 2/2A/MCUb-HA_MW.tif]

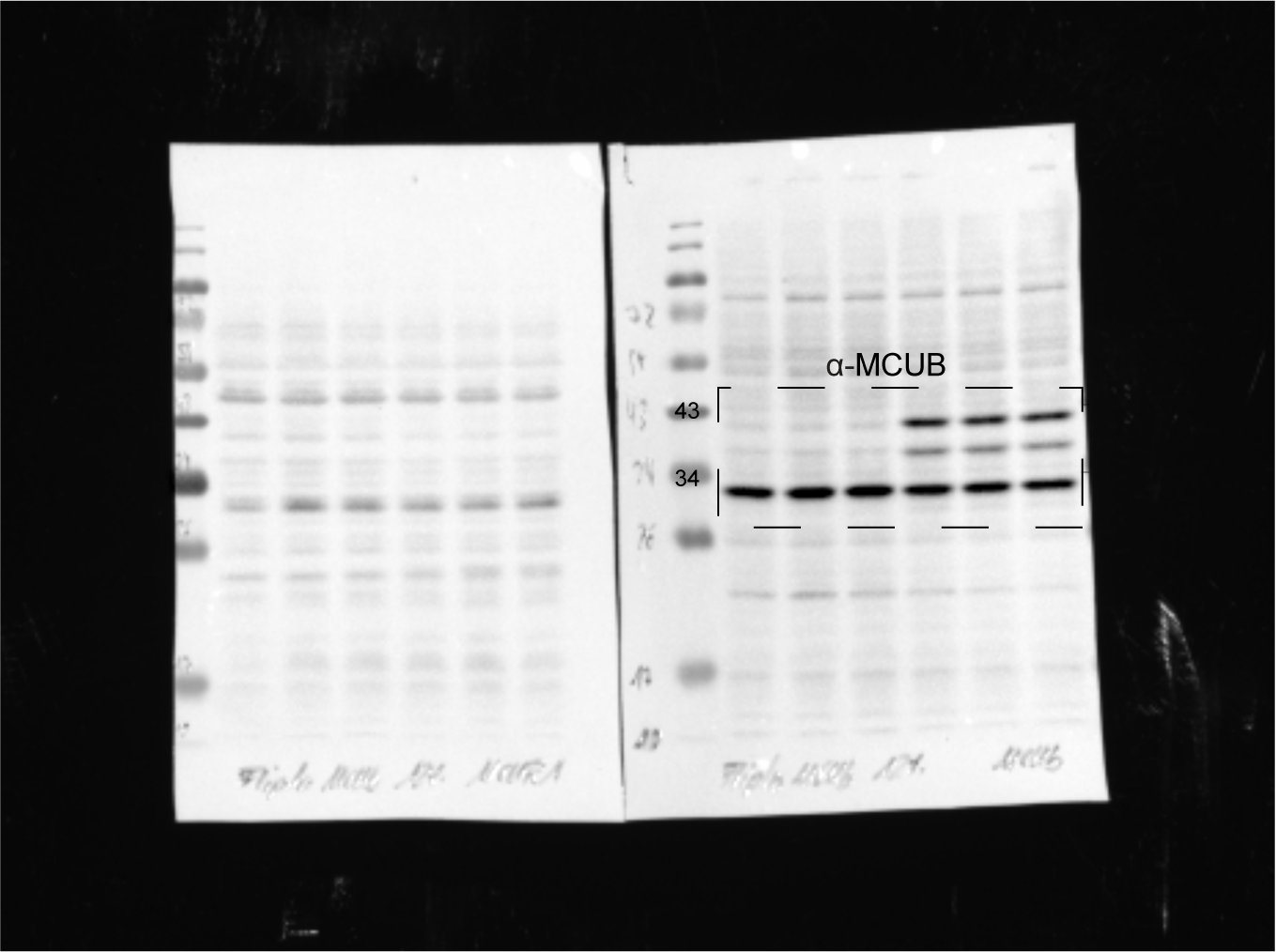

Supplement: Supplementary file 7 — Source data Fig. 2 [file 44318_2024_219_MOESM7_ESM.zip › Figure 2/2A/MCUb_MW.tif]

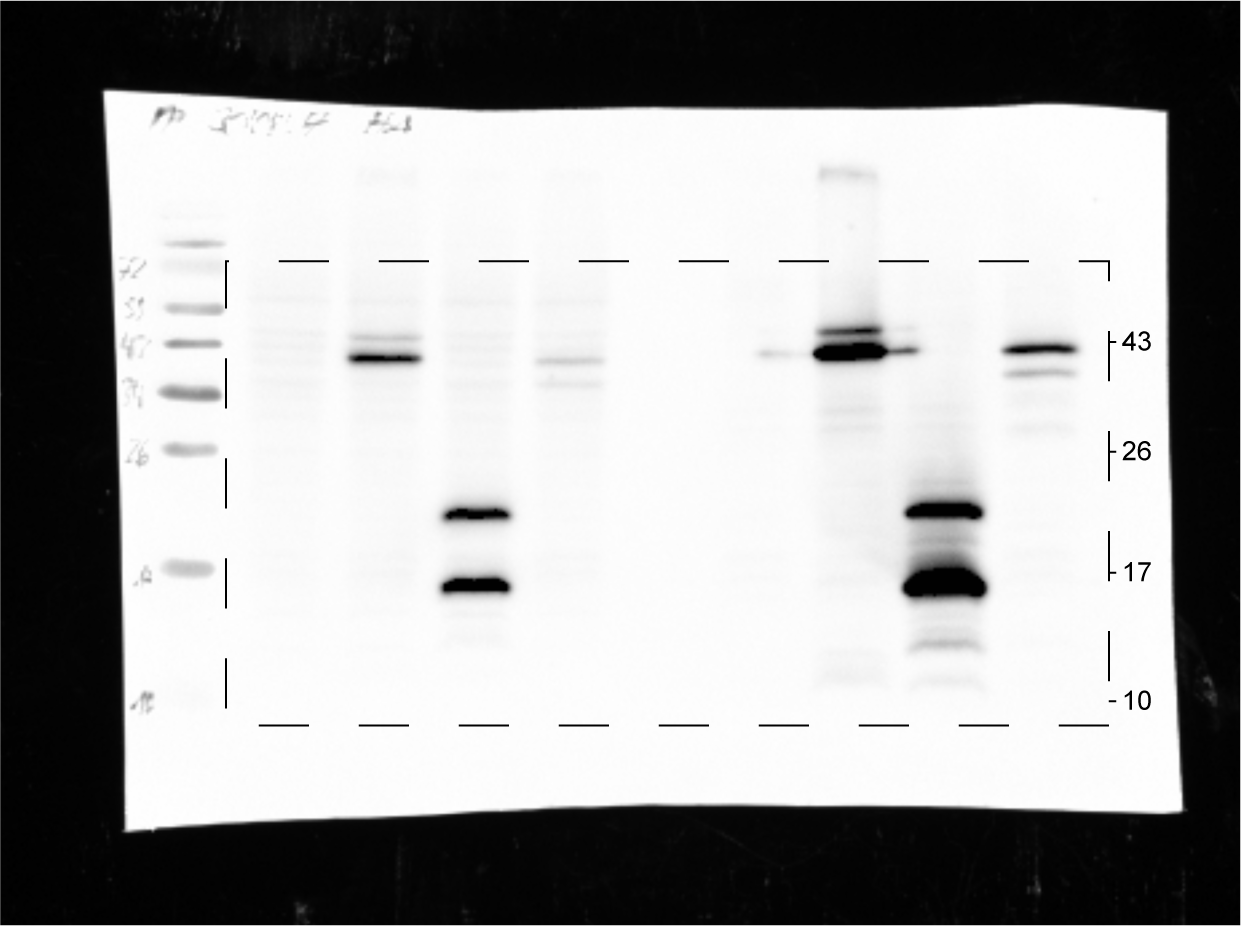

Supplement: Supplementary file 7 — Source data Fig. 2 [file 44318_2024_219_MOESM7_ESM.zip › Figure 2/2B/HA+marker_MW.tif]

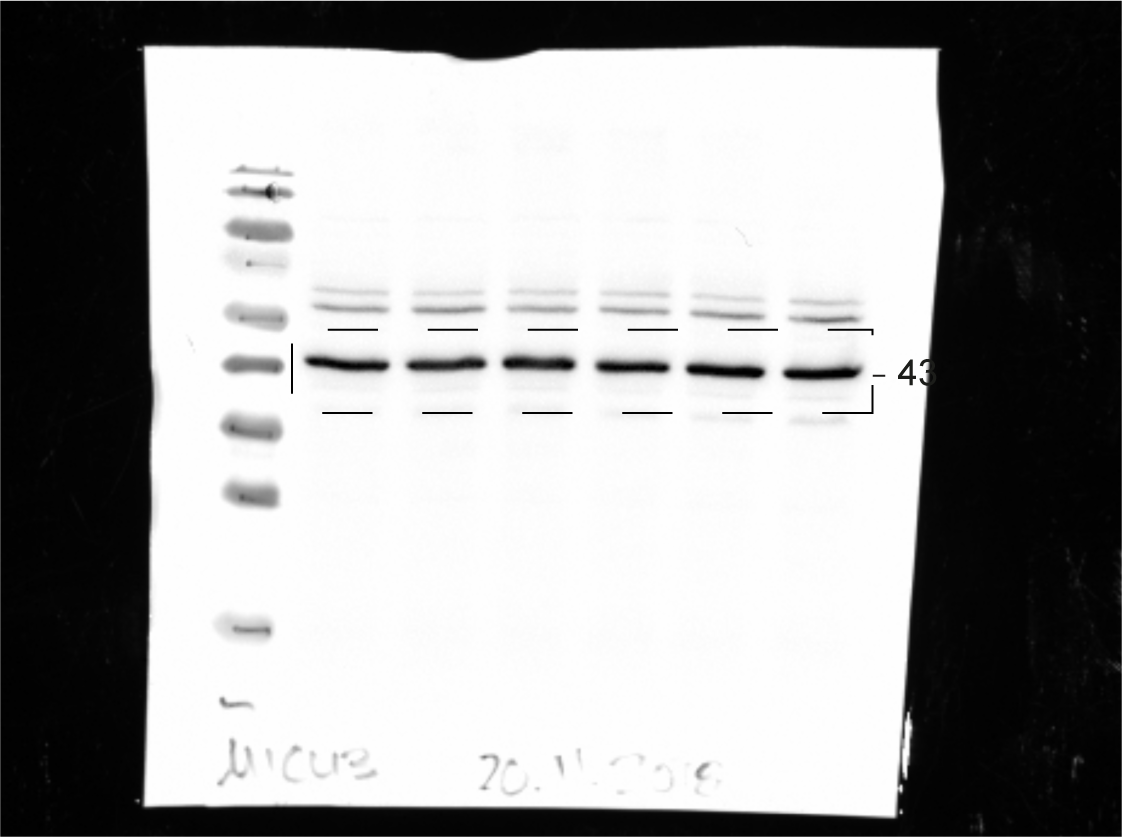

Supplement: Supplementary file 9 — Source data Fig. 4 [file 44318_2024_219_MOESM9_ESM.zip › Figure 4/4B/Actin_MW.tif]

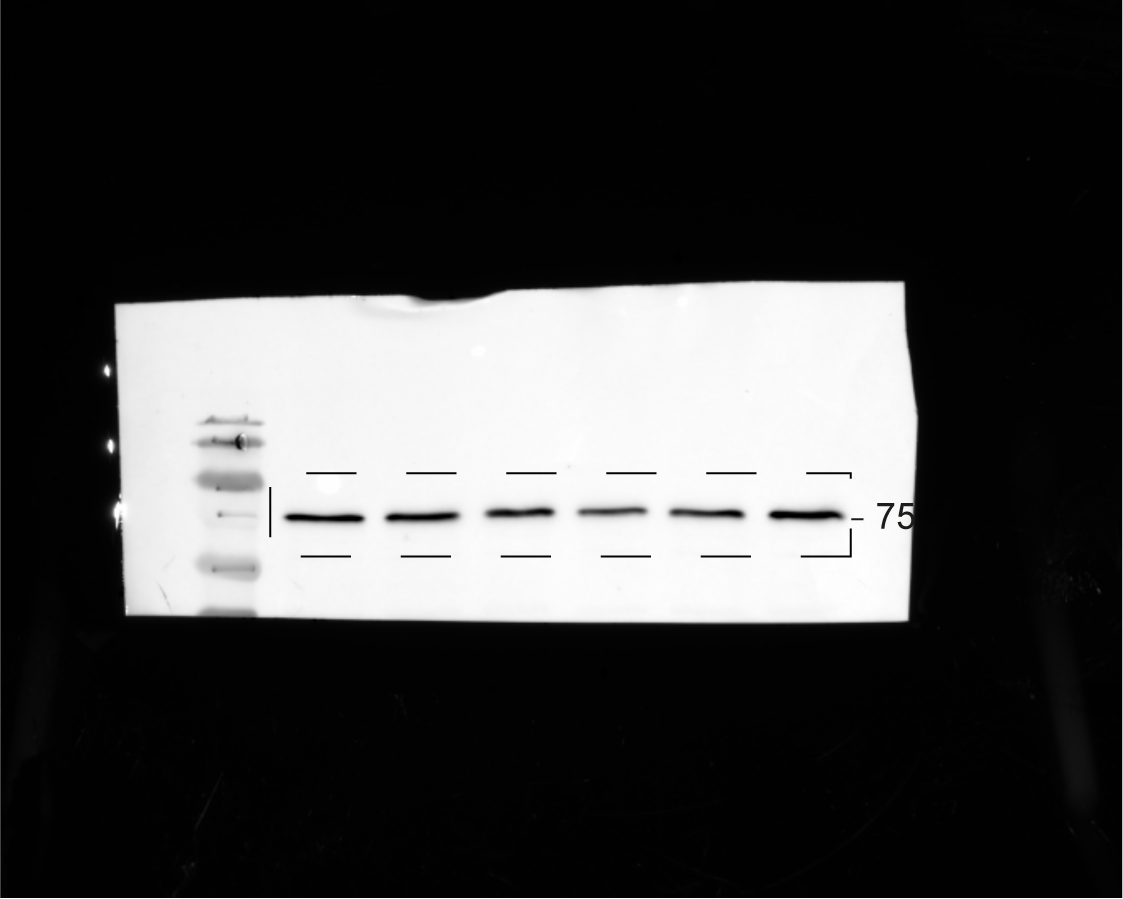

Supplement: Supplementary file 9 — Source data Fig. 4 [file 44318_2024_219_MOESM9_ESM.zip › Figure 4/4B/GRP75_MW.tif]

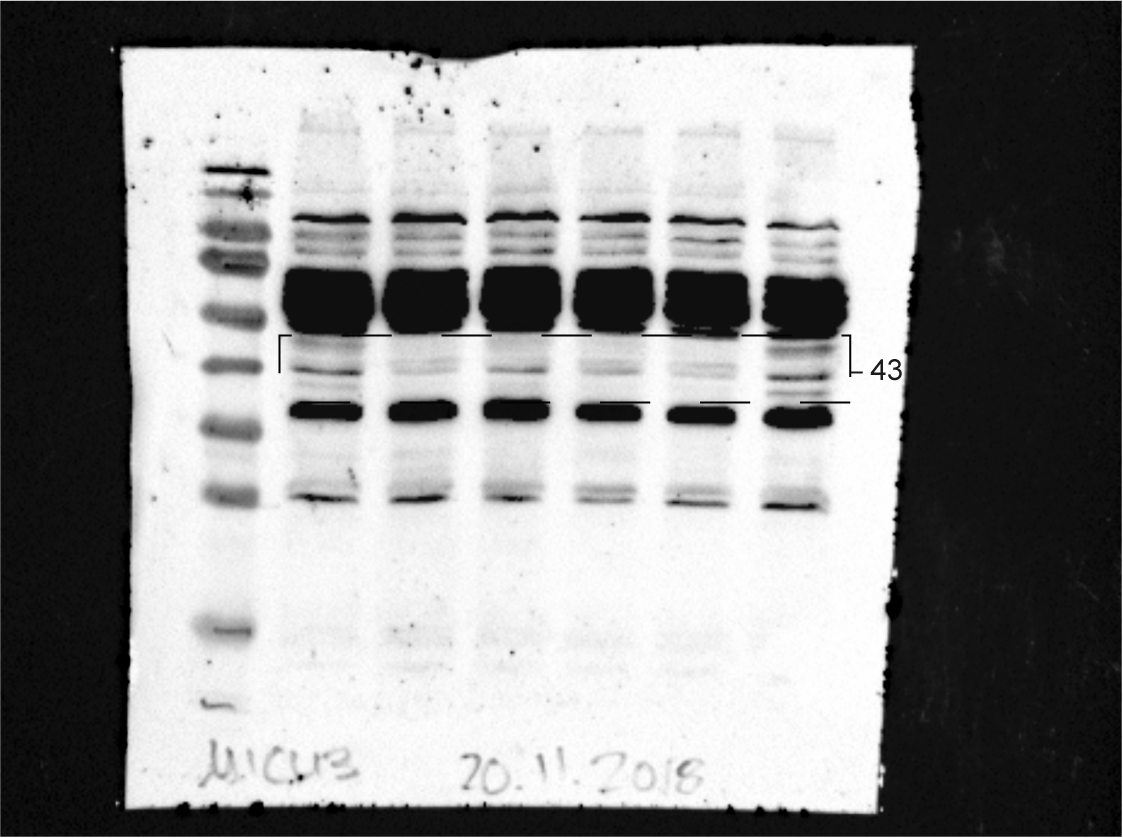

Supplement: Supplementary file 9 — Source data Fig. 4 [file 44318_2024_219_MOESM9_ESM.zip › Figure 4/4B/MICU3_MW.tif]

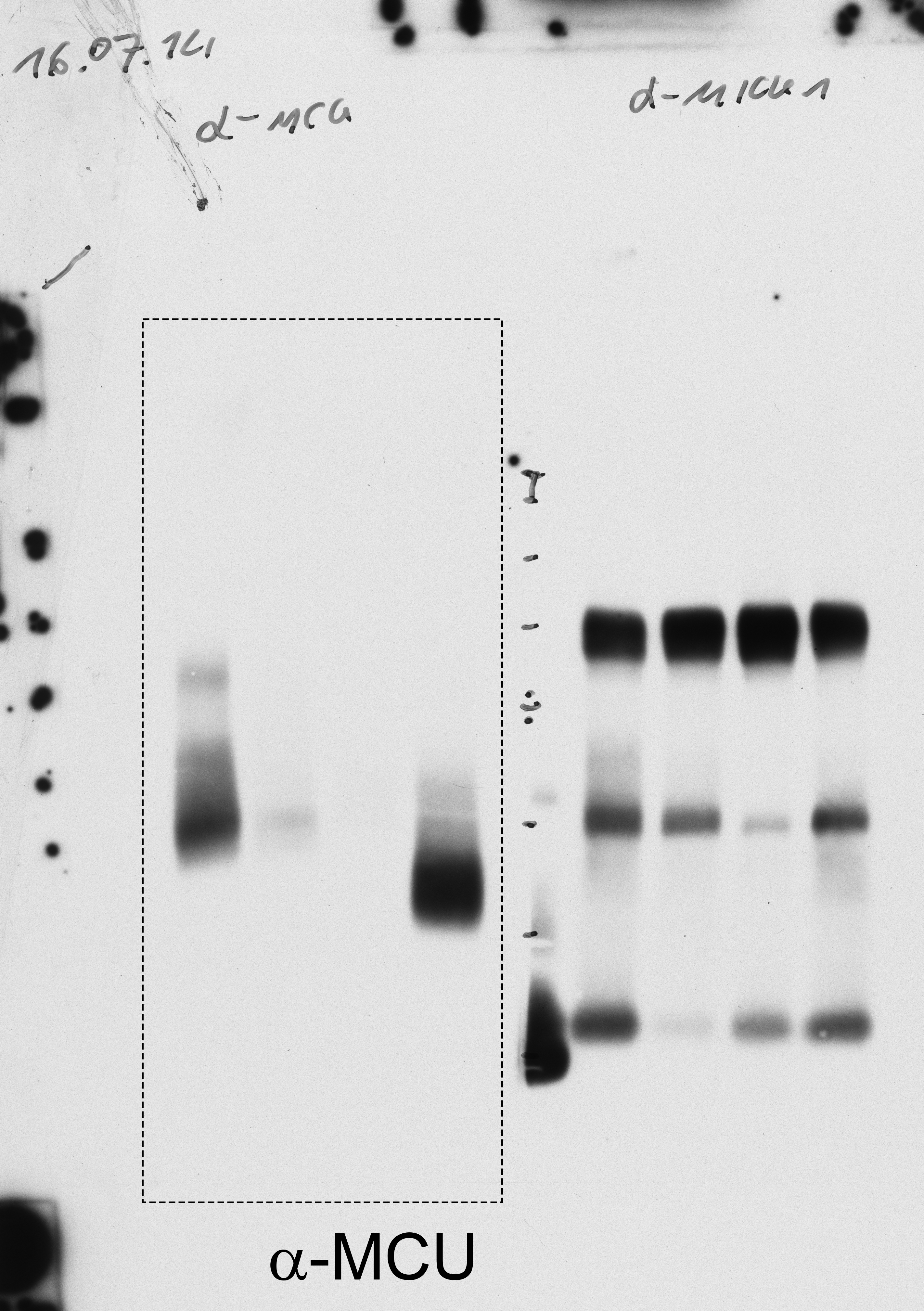

Supplement: Supplementary file 9 — Source data Fig. 4 [file 44318_2024_219_MOESM9_ESM.zip › Figure 4/4D/Fig 4D original scan.tif]

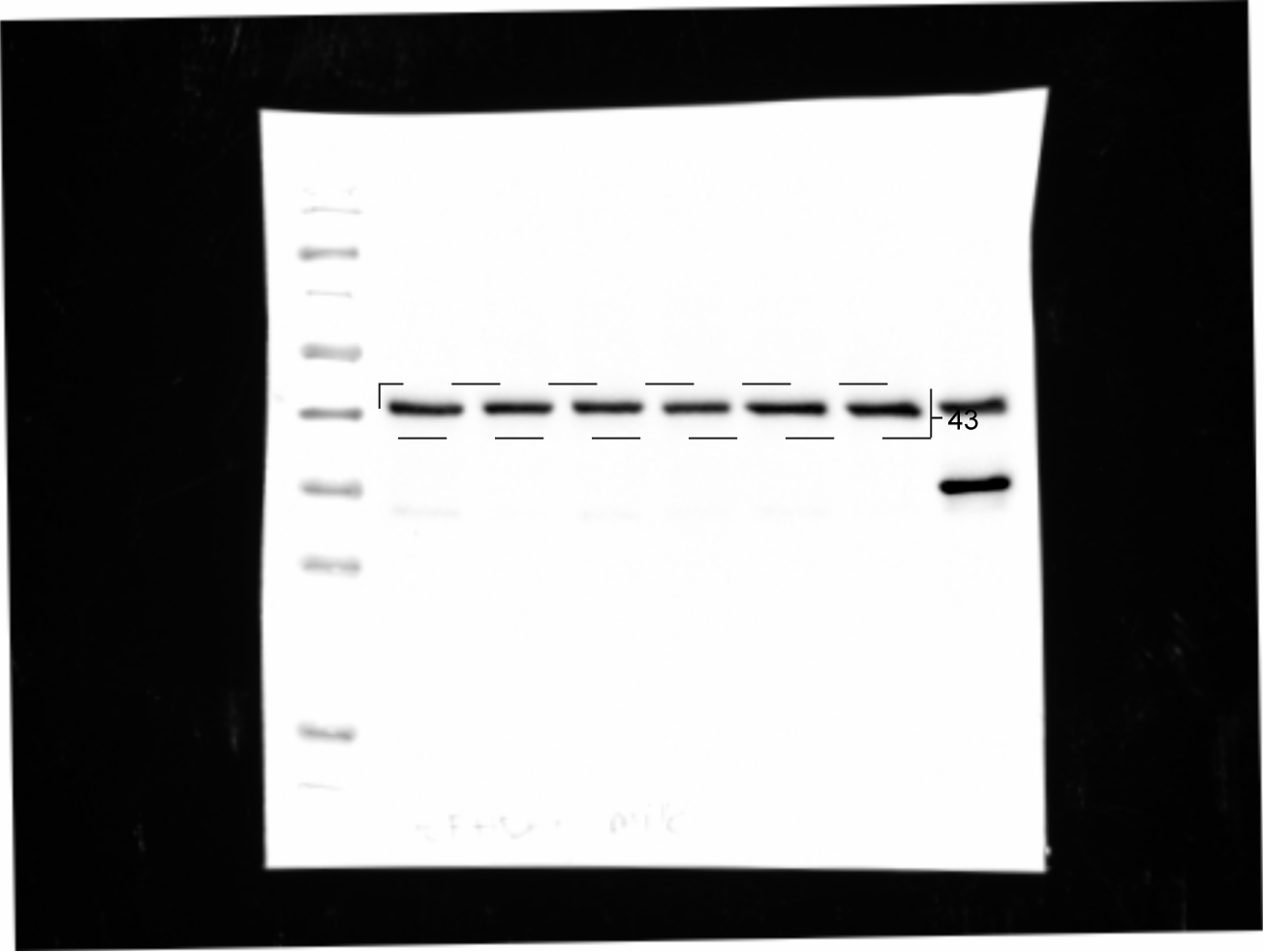

Supplement: Supplementary file 9 — Source data Fig. 4 [file 44318_2024_219_MOESM9_ESM.zip › Figure 4/4G/Actin_MW.tif]

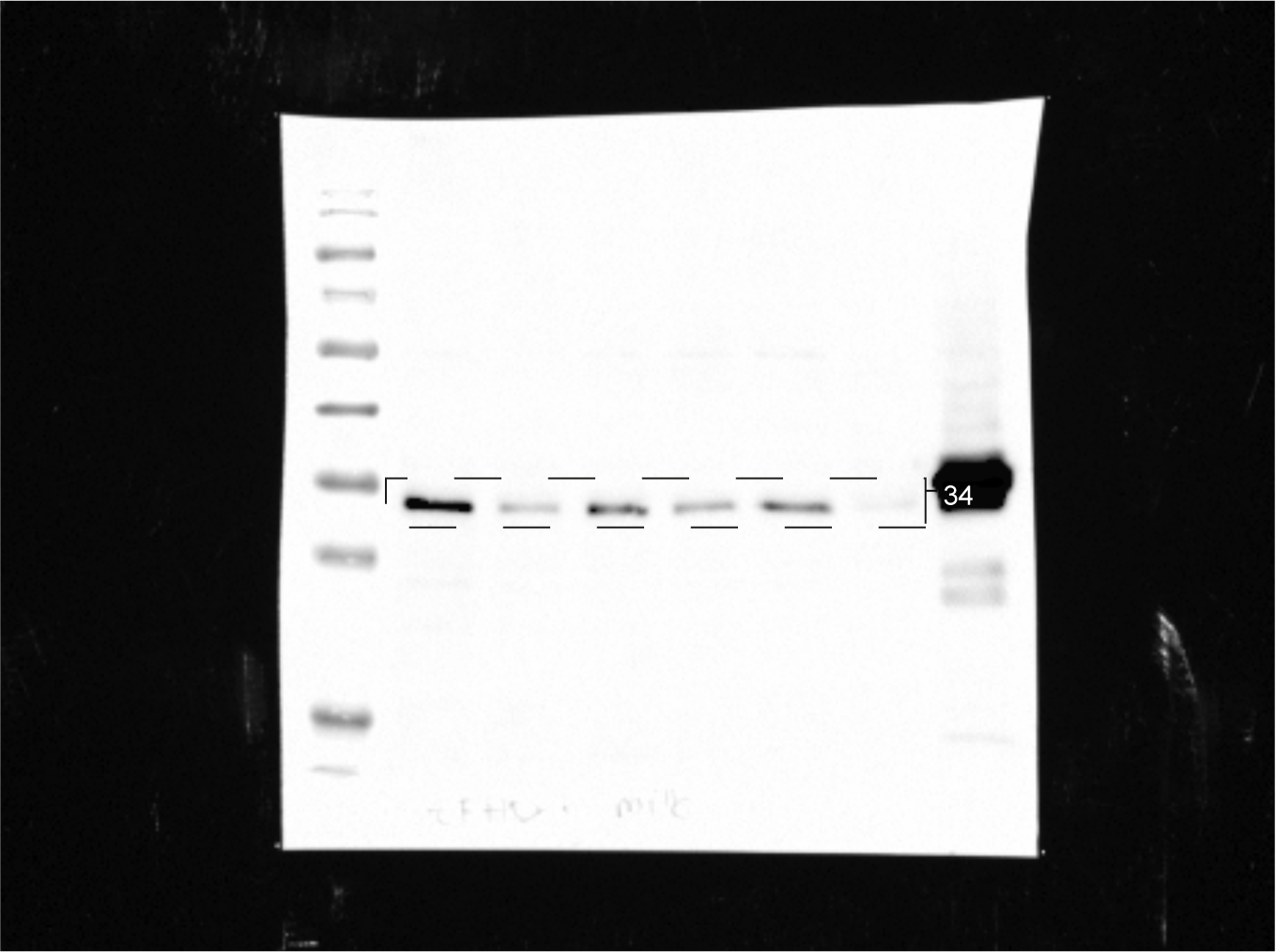

Supplement: Supplementary file 9 — Source data Fig. 4 [file 44318_2024_219_MOESM9_ESM.zip › Figure 4/4G/EFHD1_MW.tif]

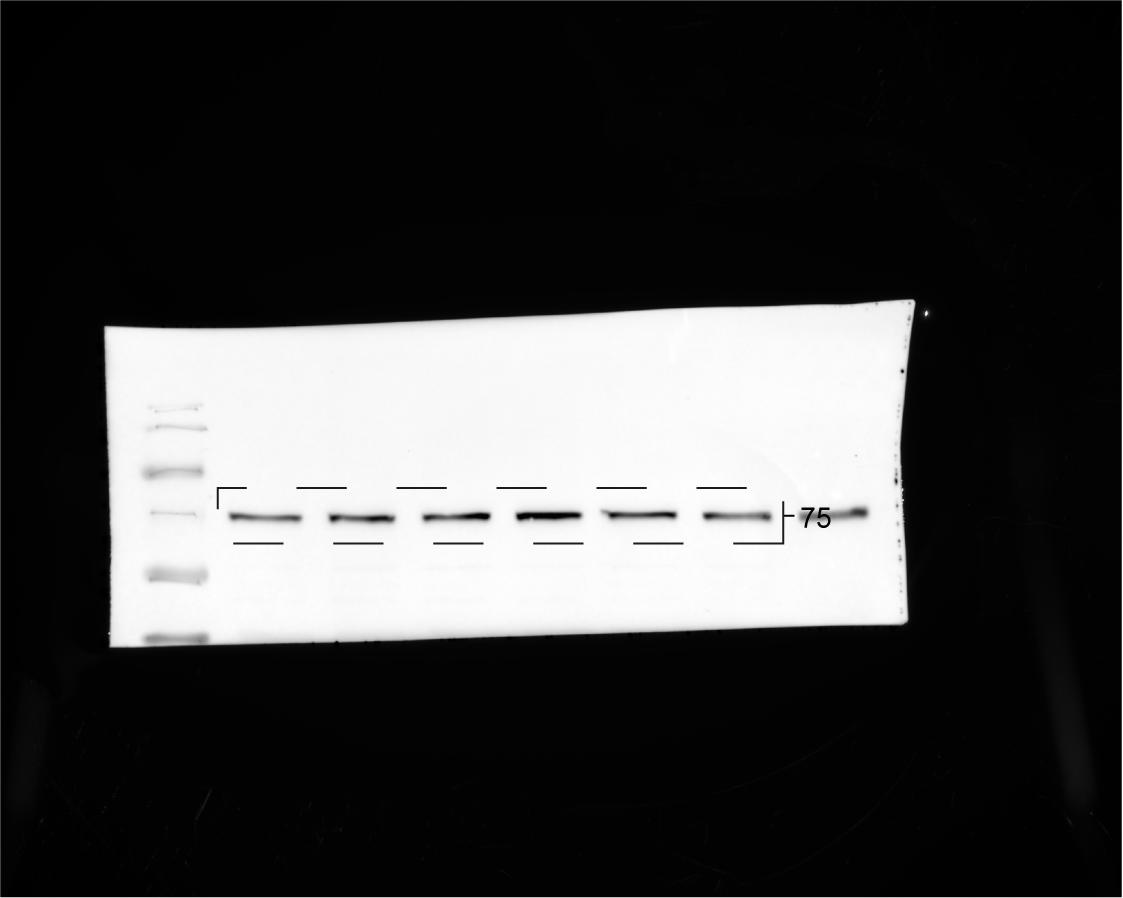

Supplement: Supplementary file 9 — Source data Fig. 4 [file 44318_2024_219_MOESM9_ESM.zip › Figure 4/4G/GRP75_MW.tif]

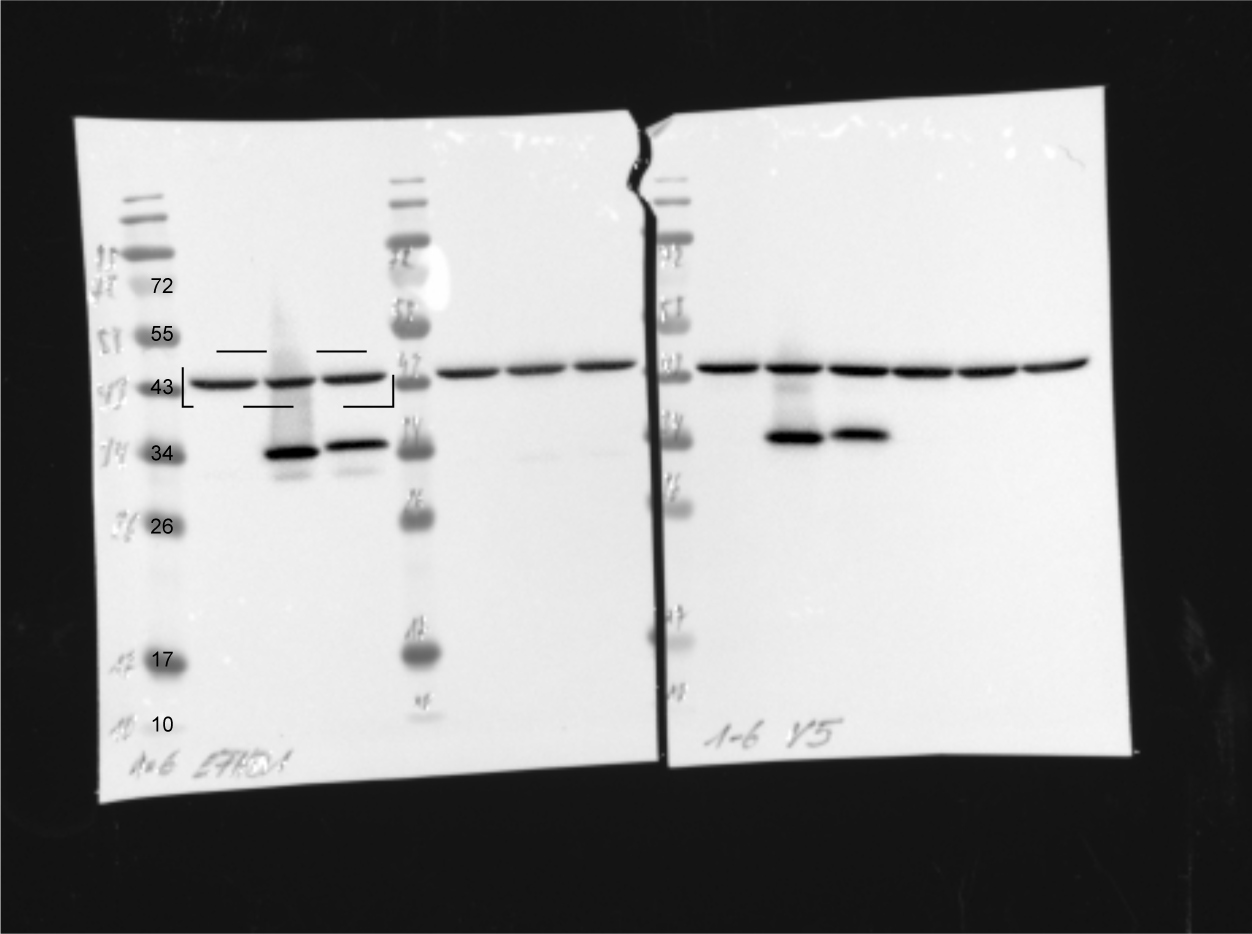

Supplement: Supplementary file 9 — Source data Fig. 4 [file 44318_2024_219_MOESM9_ESM.zip › Figure 4/4L/anti-Actin_MW.tif]

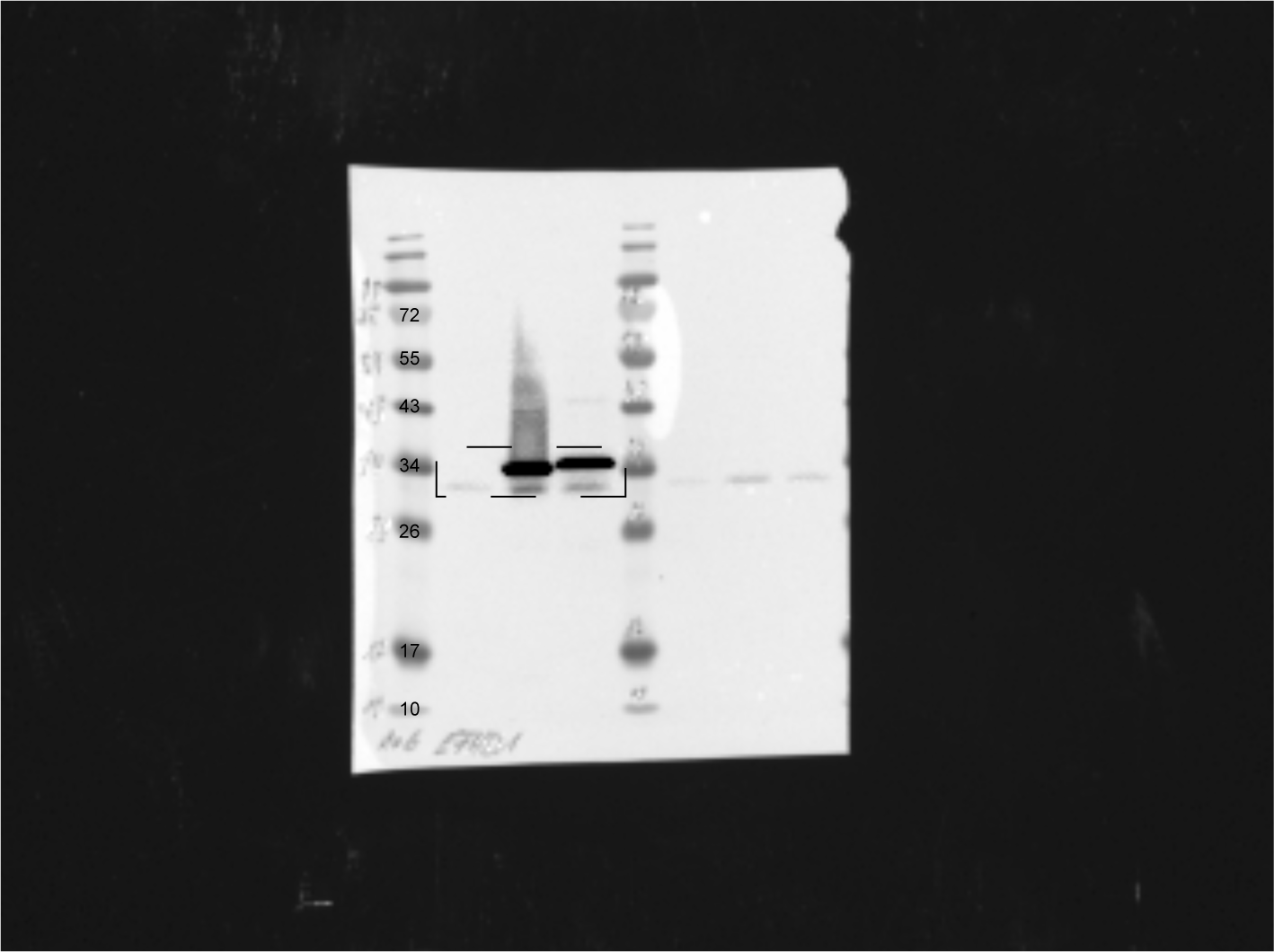

Supplement: Supplementary file 9 — Source data Fig. 4 [file 44318_2024_219_MOESM9_ESM.zip › Figure 4/4L/anti-EFHD1_MW.tif]

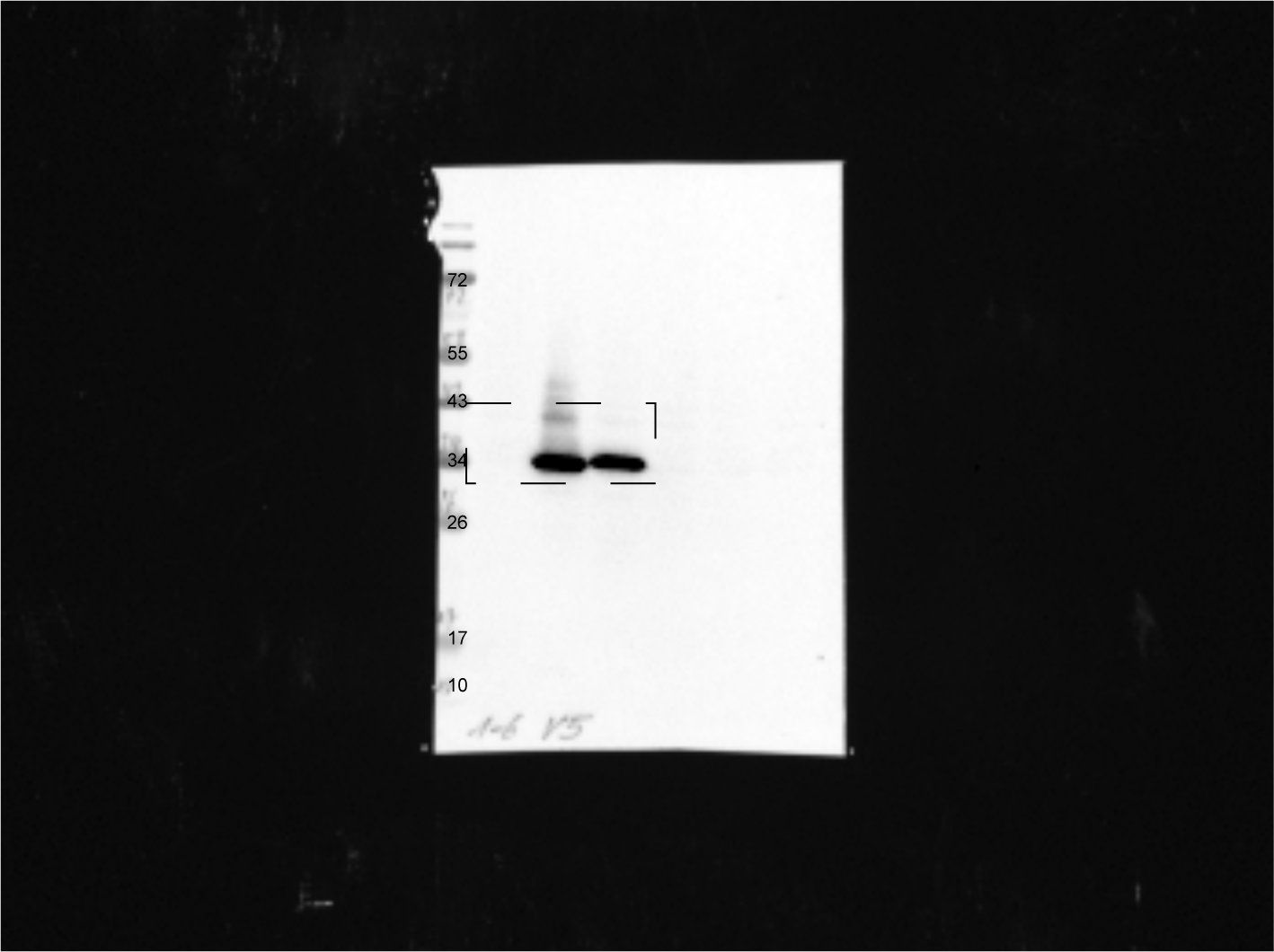

Supplement: Supplementary file 9 — Source data Fig. 4 [file 44318_2024_219_MOESM9_ESM.zip › Figure 4/4L/anti-V5_MW.tif]

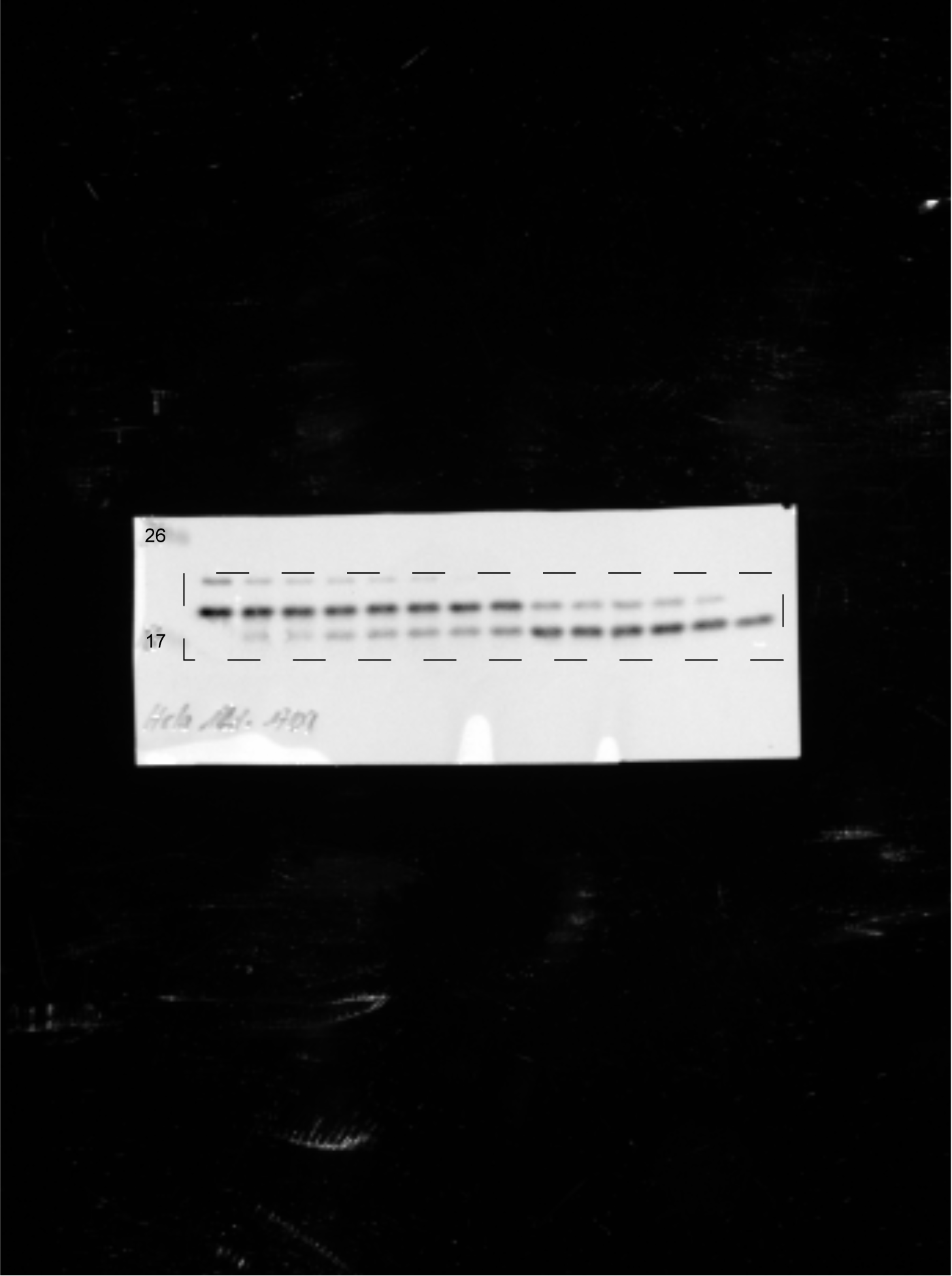

Supplement: Supplementary file 10 — Source data Fig. 5 [file 44318_2024_219_MOESM10_ESM.zip › Figure 5/5D/Cyclophillin_MW.tif]

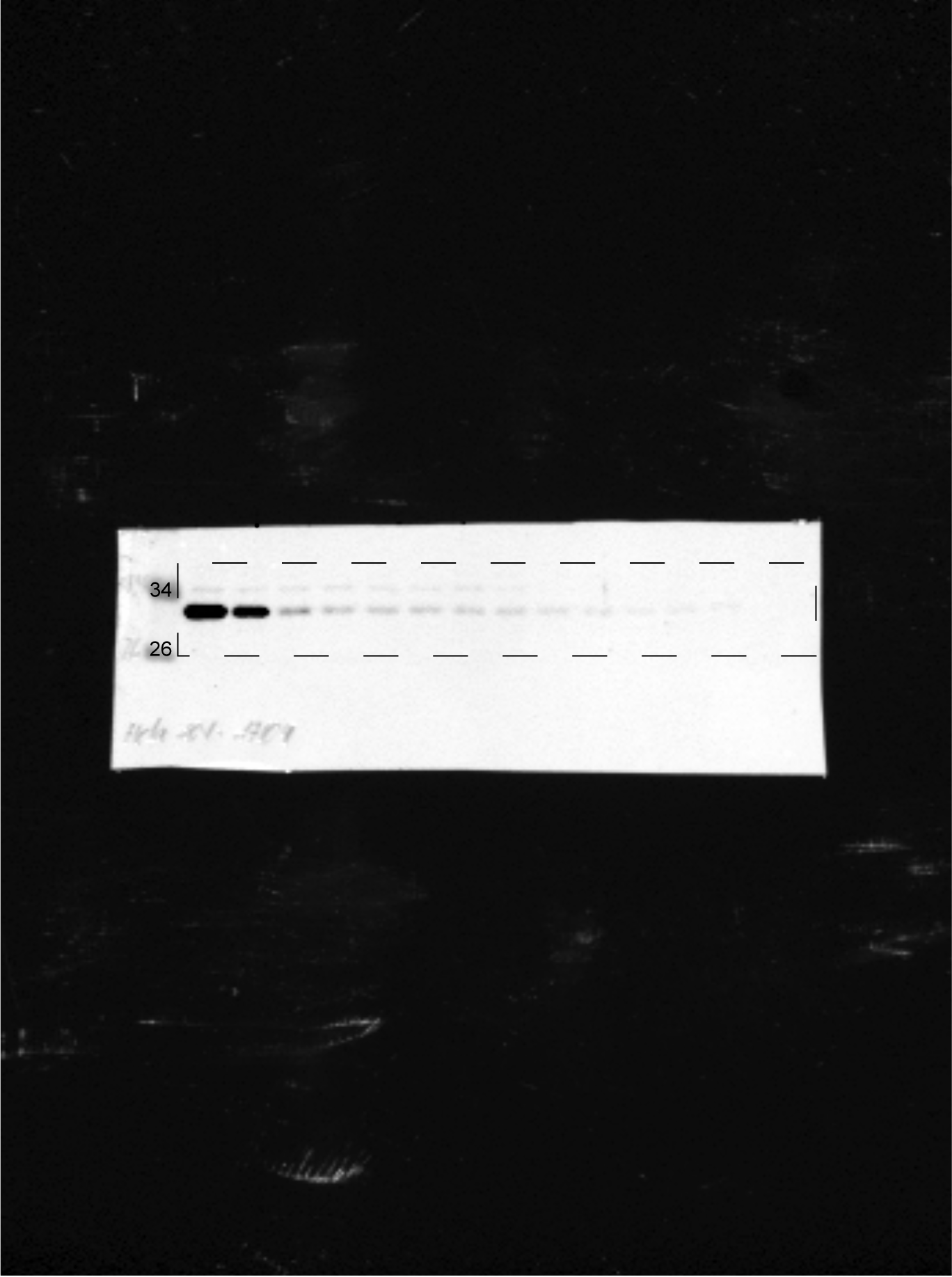

Supplement: Supplementary file 10 — Source data Fig. 5 [file 44318_2024_219_MOESM10_ESM.zip › Figure 5/5D/EFHD1_MW.tif]

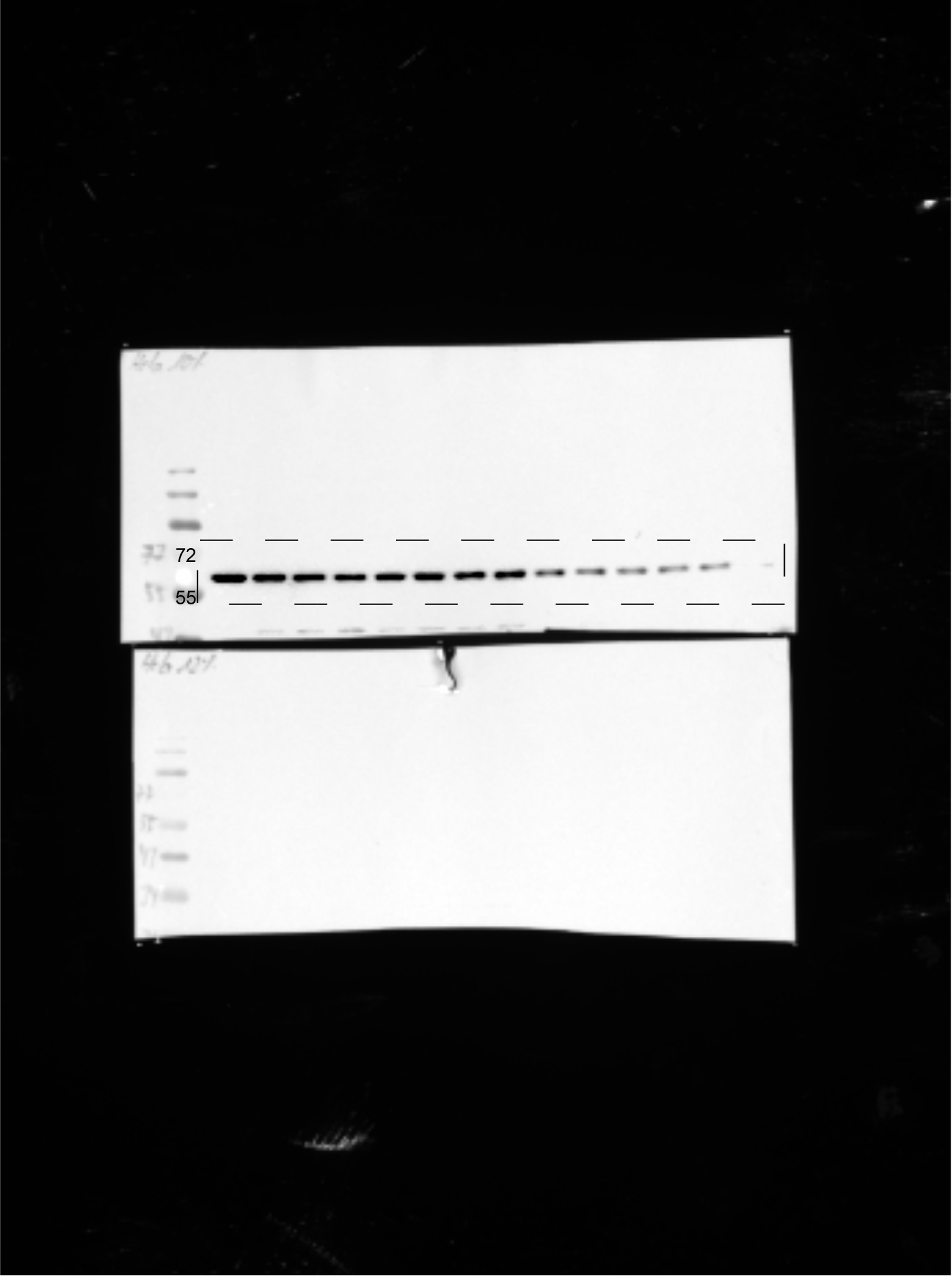

Supplement: Supplementary file 10 — Source data Fig. 5 [file 44318_2024_219_MOESM10_ESM.zip › Figure 5/5D/HSP60_MW.tif]

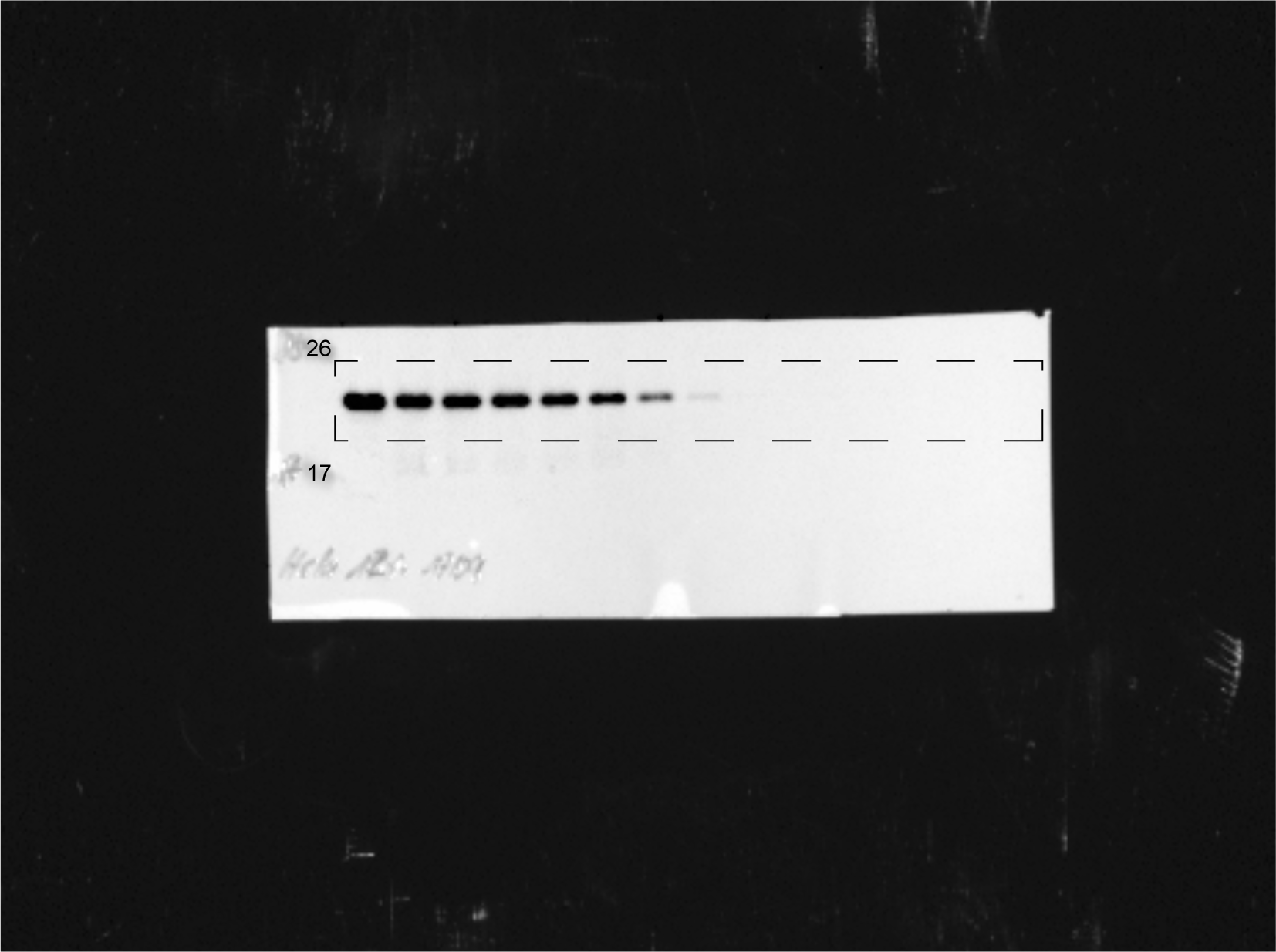

Supplement: Supplementary file 10 — Source data Fig. 5 [file 44318_2024_219_MOESM10_ESM.zip › Figure 5/5D/TIM23_MW.tif]

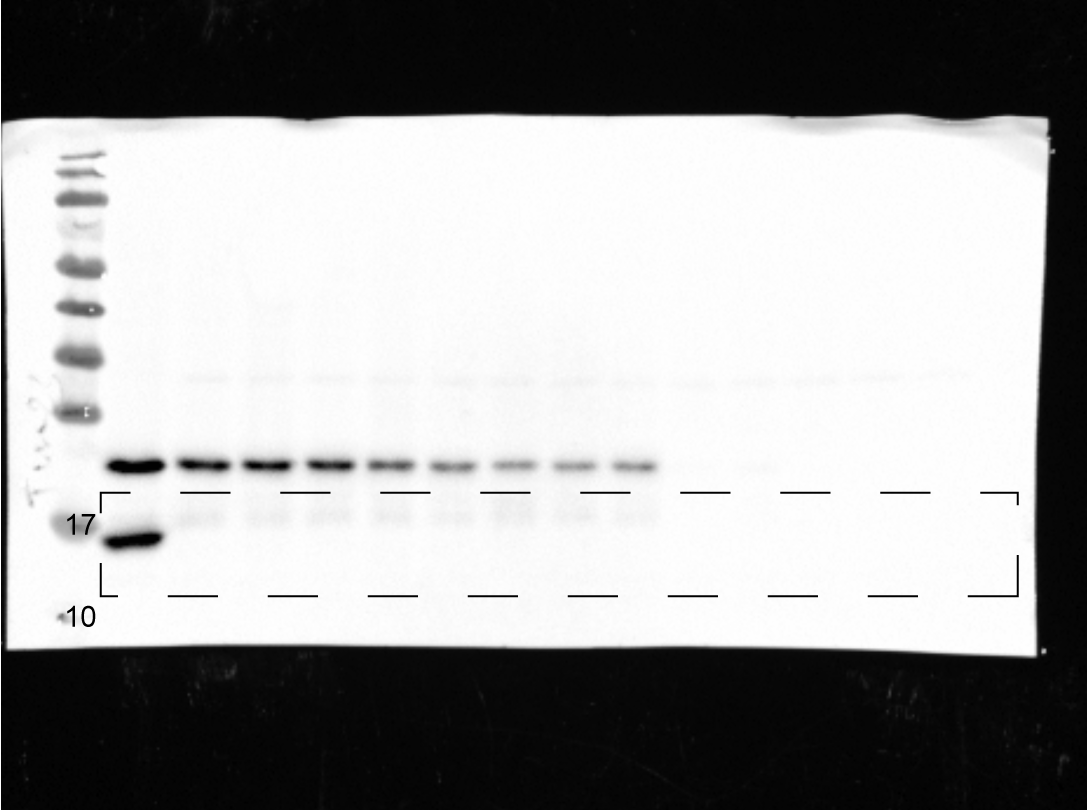

Supplement: Supplementary file 10 — Source data Fig. 5 [file 44318_2024_219_MOESM10_ESM.zip › Figure 5/5D/TOM20_MW.tif]

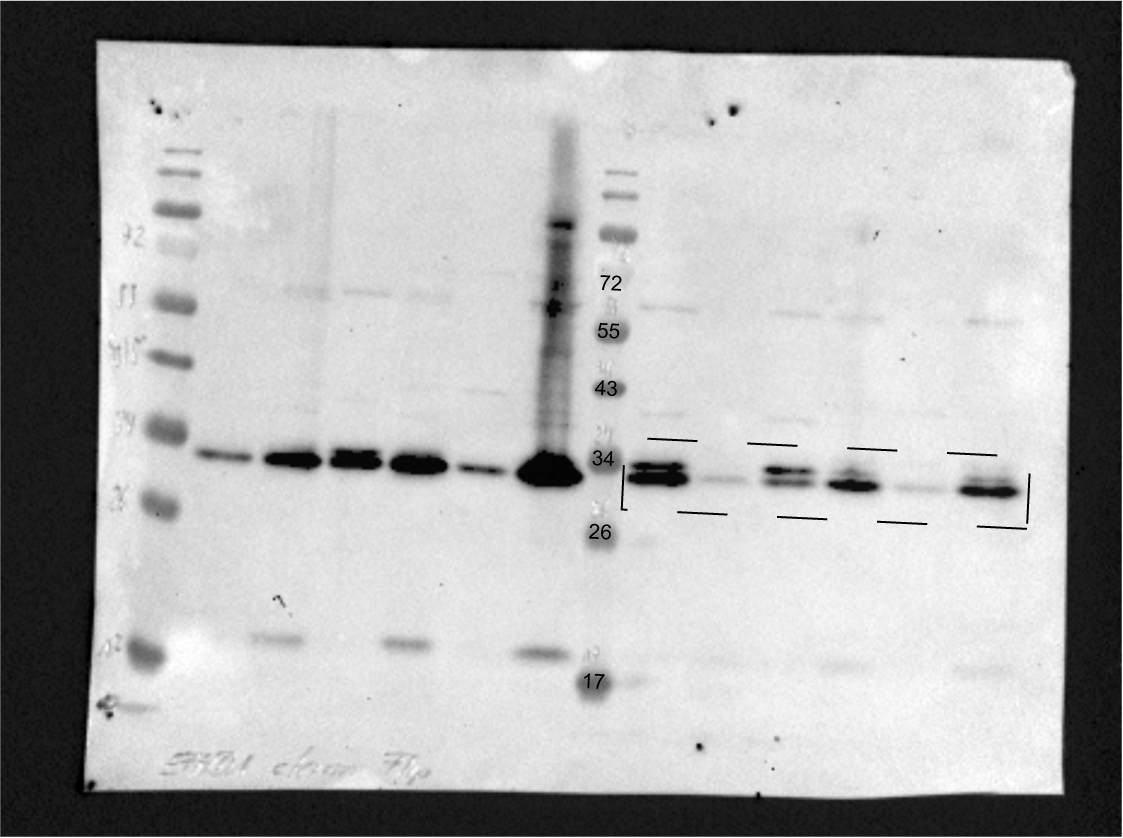

Supplement: Supplementary file 10 — Source data Fig. 5 [file 44318_2024_219_MOESM10_ESM.zip › Figure 5/5E/EFHD1_HEK.tif]

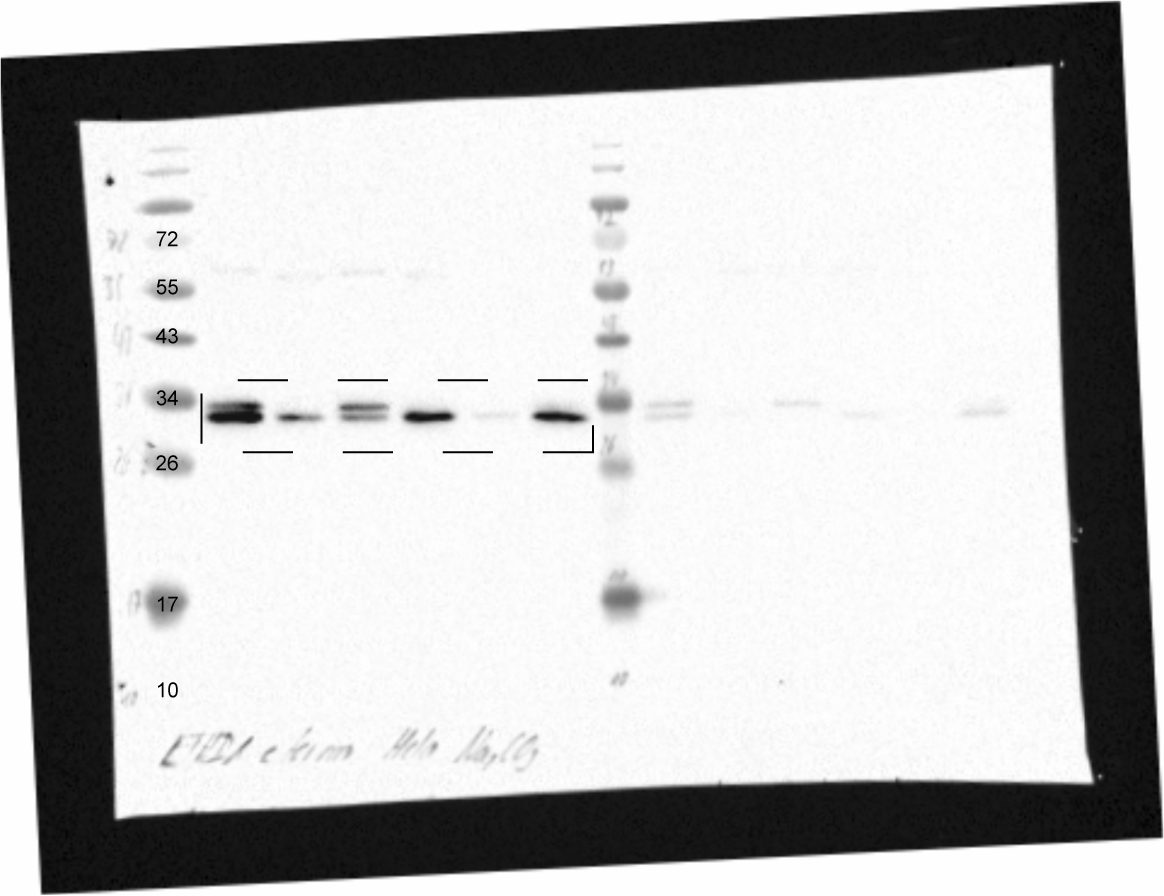

Supplement: Supplementary file 10 — Source data Fig. 5 [file 44318_2024_219_MOESM10_ESM.zip › Figure 5/5E/EFHD1_HeLa_MW.tif]

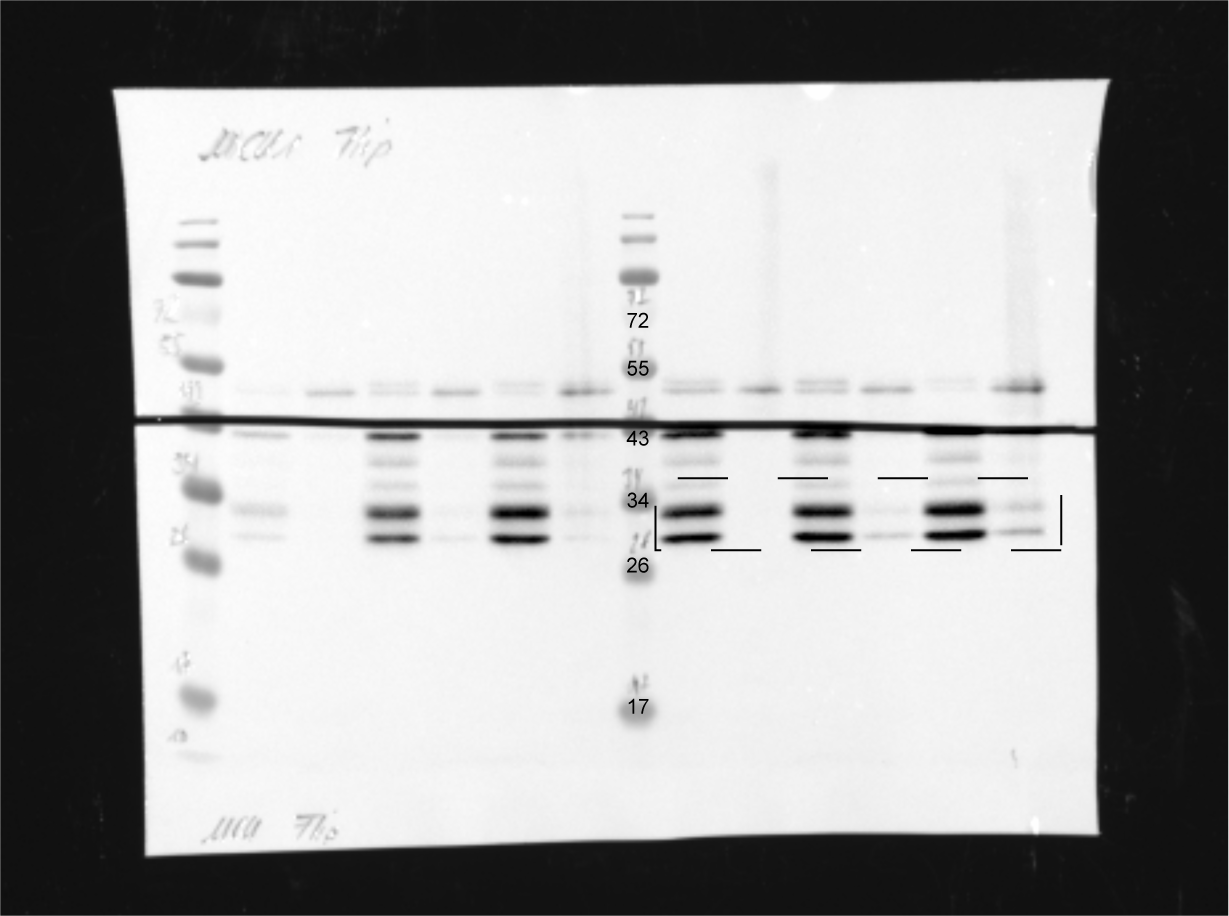

Supplement: Supplementary file 10 — Source data Fig. 5 [file 44318_2024_219_MOESM10_ESM.zip › Figure 5/5E/MCU_HEK.tif]

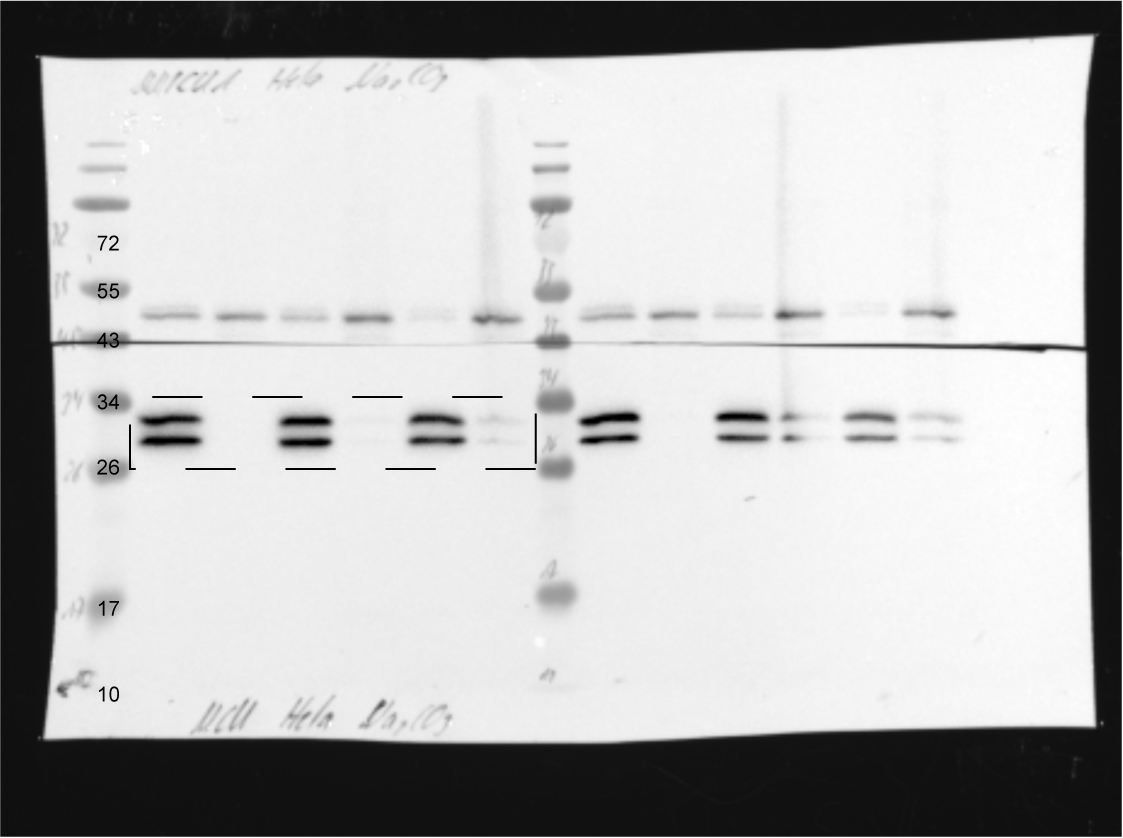

Supplement: Supplementary file 10 — Source data Fig. 5 [file 44318_2024_219_MOESM10_ESM.zip › Figure 5/5E/MCU_HeLa_MW.tif]

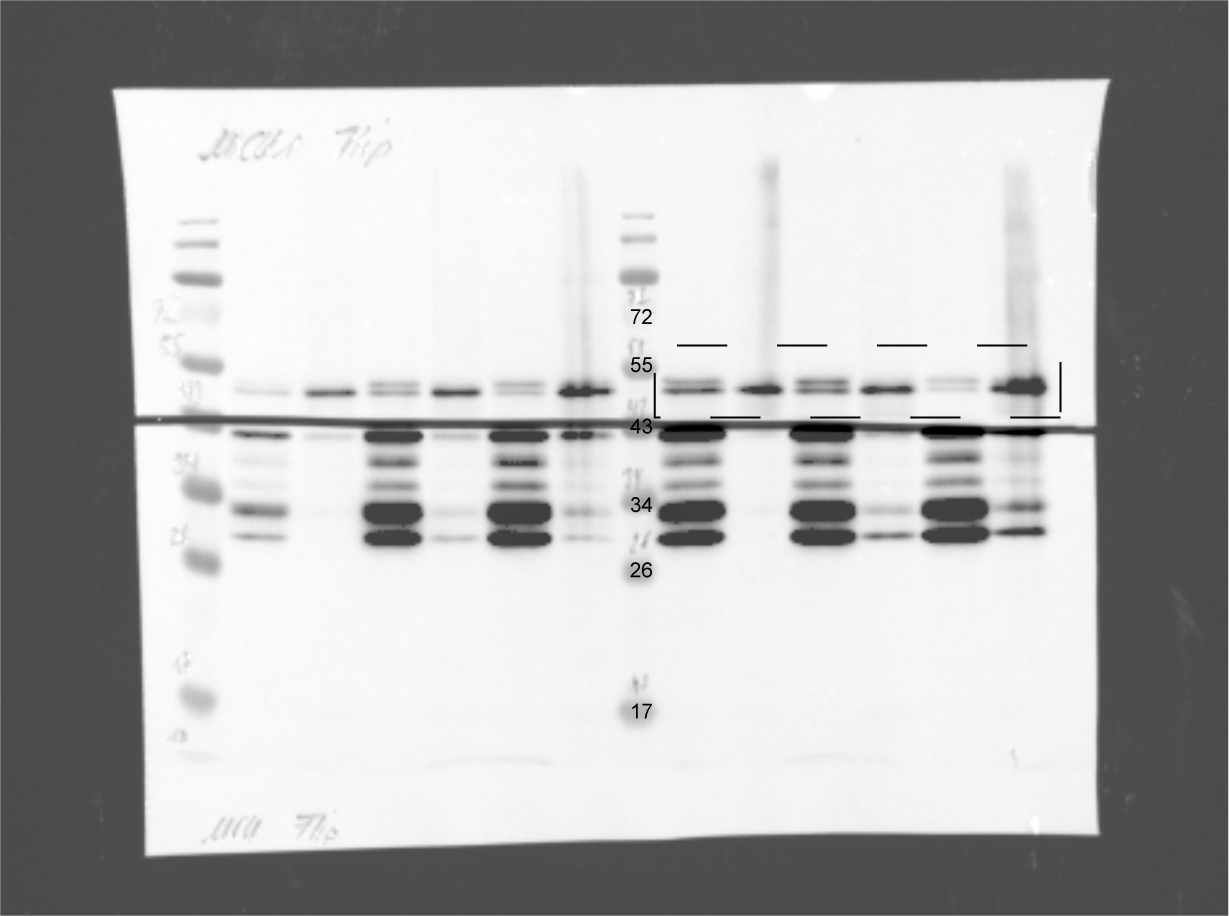

Supplement: Supplementary file 10 — Source data Fig. 5 [file 44318_2024_219_MOESM10_ESM.zip › Figure 5/5E/MICU1_HEK.tif]

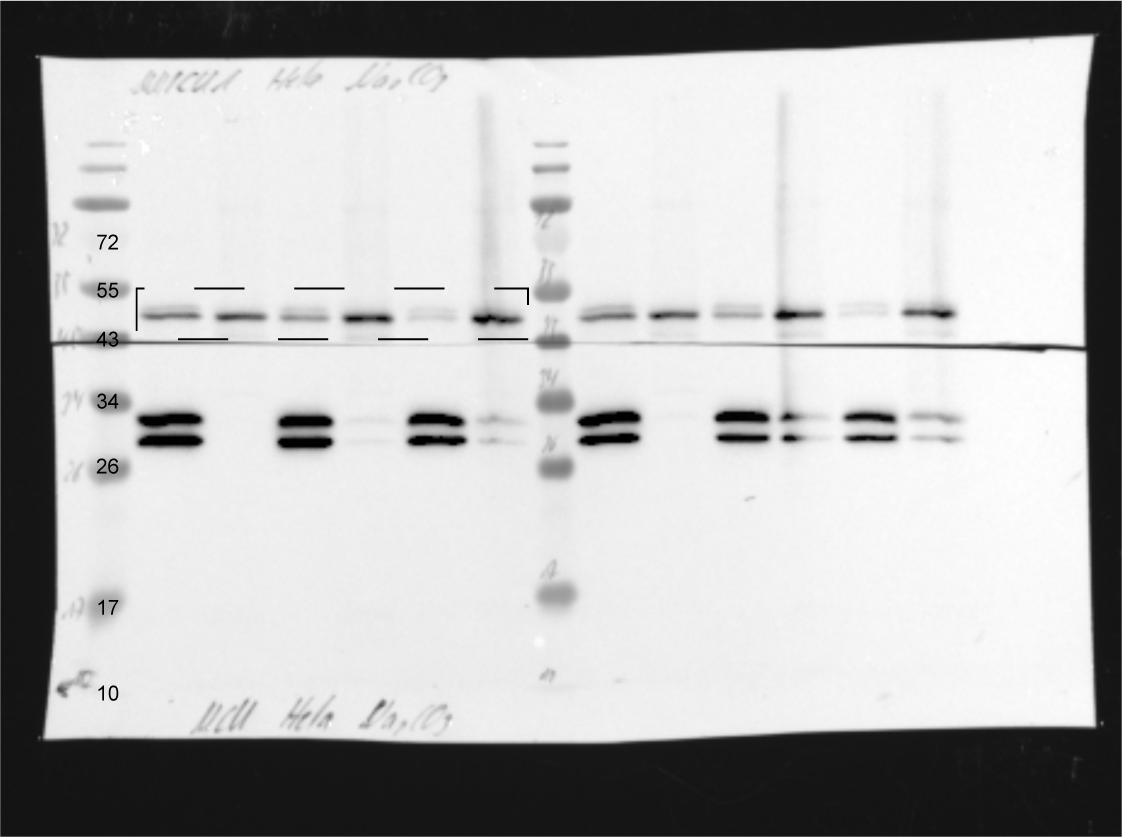

Supplement: Supplementary file 10 — Source data Fig. 5 [file 44318_2024_219_MOESM10_ESM.zip › Figure 5/5E/MICU1_HeLa_MW.tif]

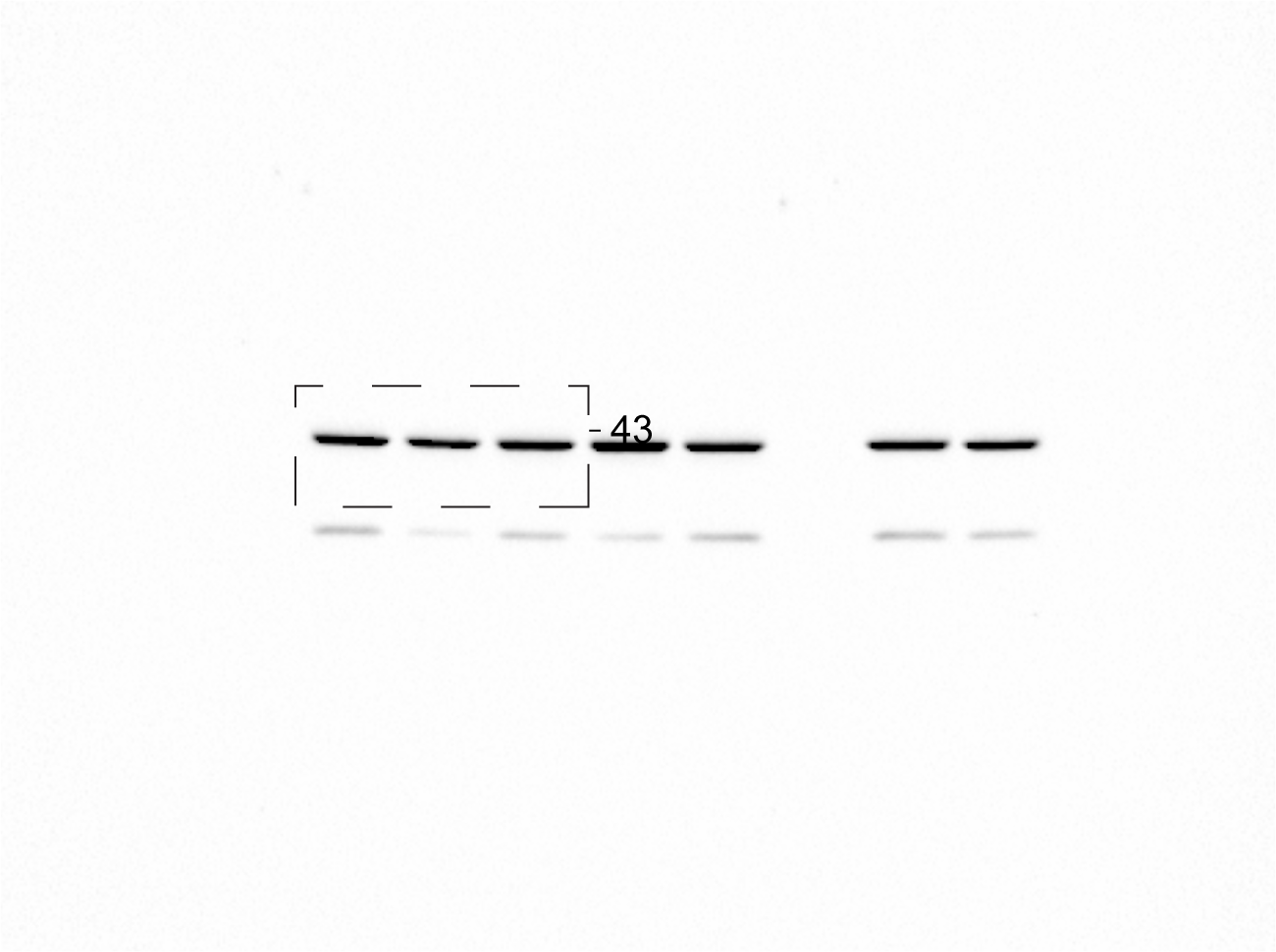

Supplement: Supplementary file 10 — Source data Fig. 5 [file 44318_2024_219_MOESM10_ESM.zip › Figure 5/5F/Actin_MW.tif]

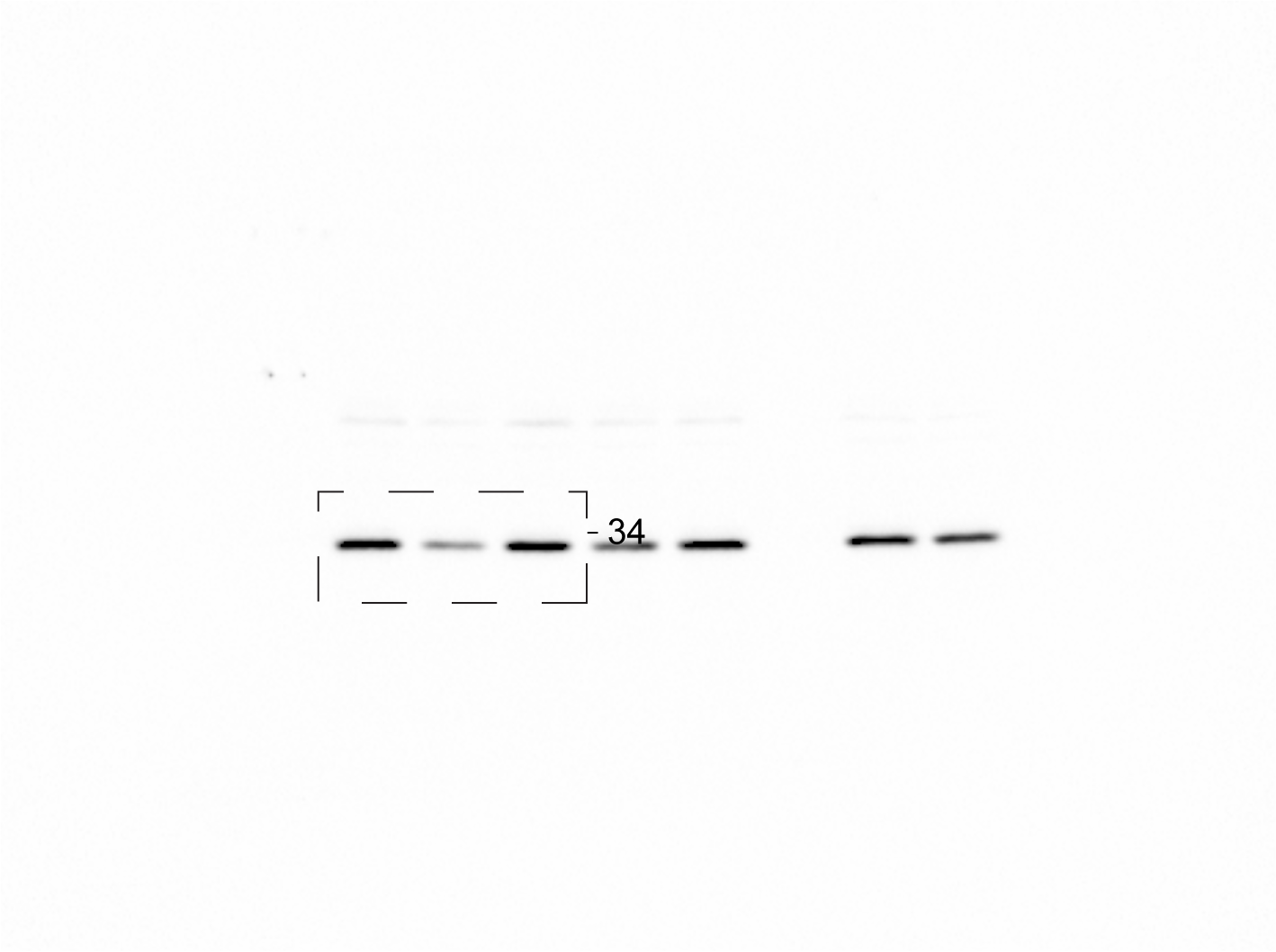

Supplement: Supplementary file 10 — Source data Fig. 5 [file 44318_2024_219_MOESM10_ESM.zip › Figure 5/5F/EFHD1_MW.tif]

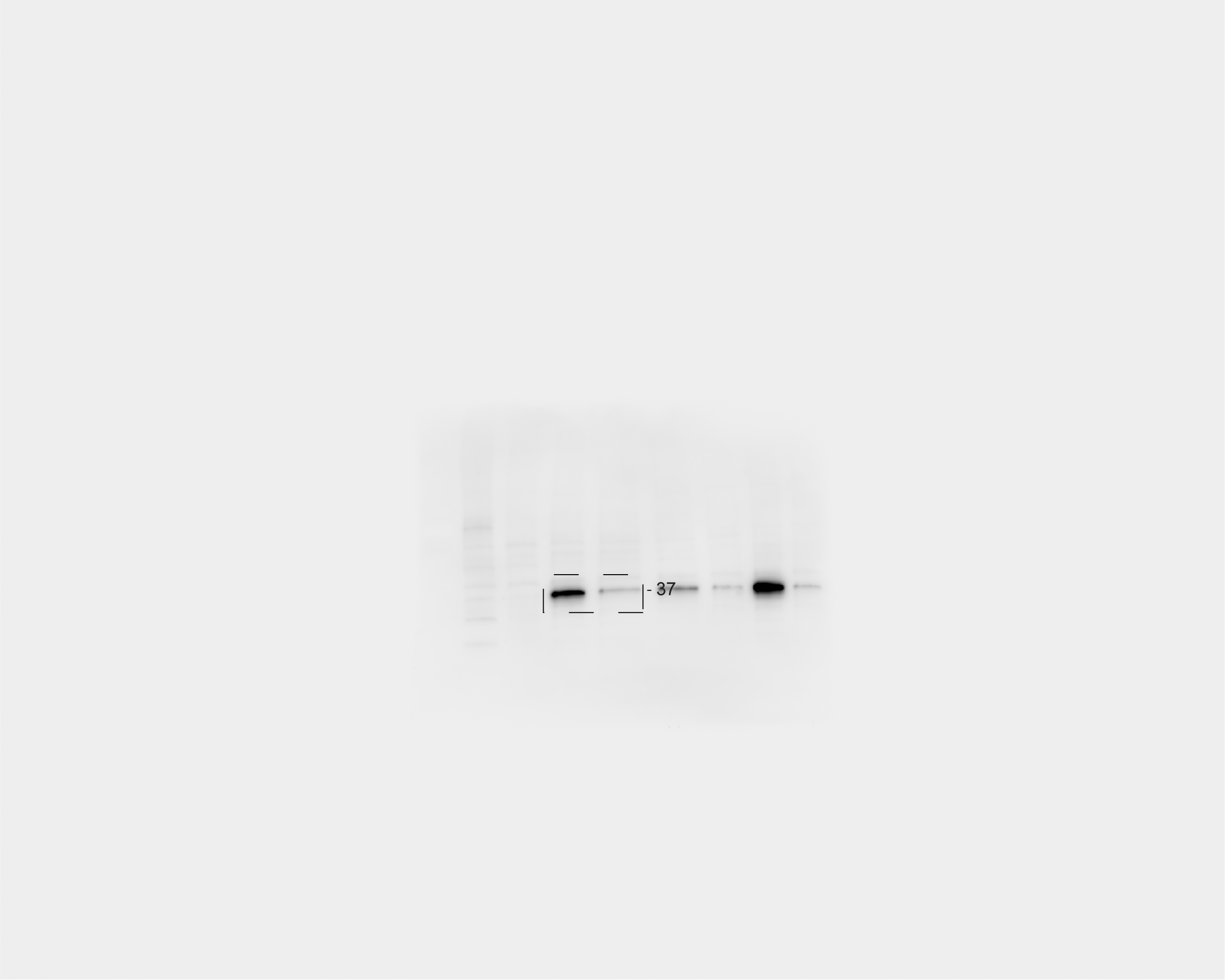

Supplement: Supplementary file 10 — Source data Fig. 5 [file 44318_2024_219_MOESM10_ESM.zip › Figure 5/5J/EFHD1_MW.tif]

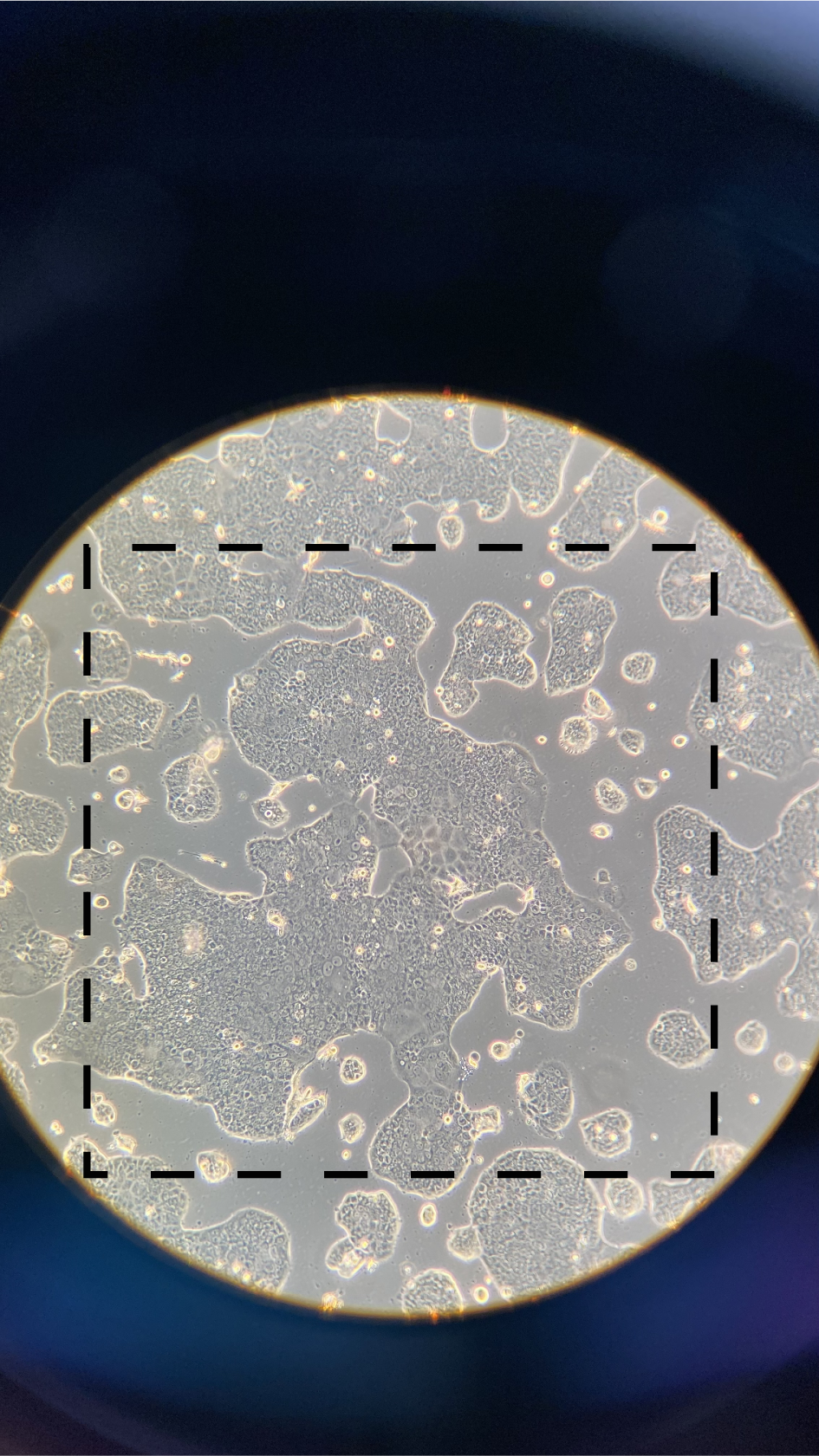

Supplement: Supplementary file 10 — Source data Fig. 5 [file 44318_2024_219_MOESM10_ESM.zip › Figure 5/5J/EFM19 pLKO with crop.tif]

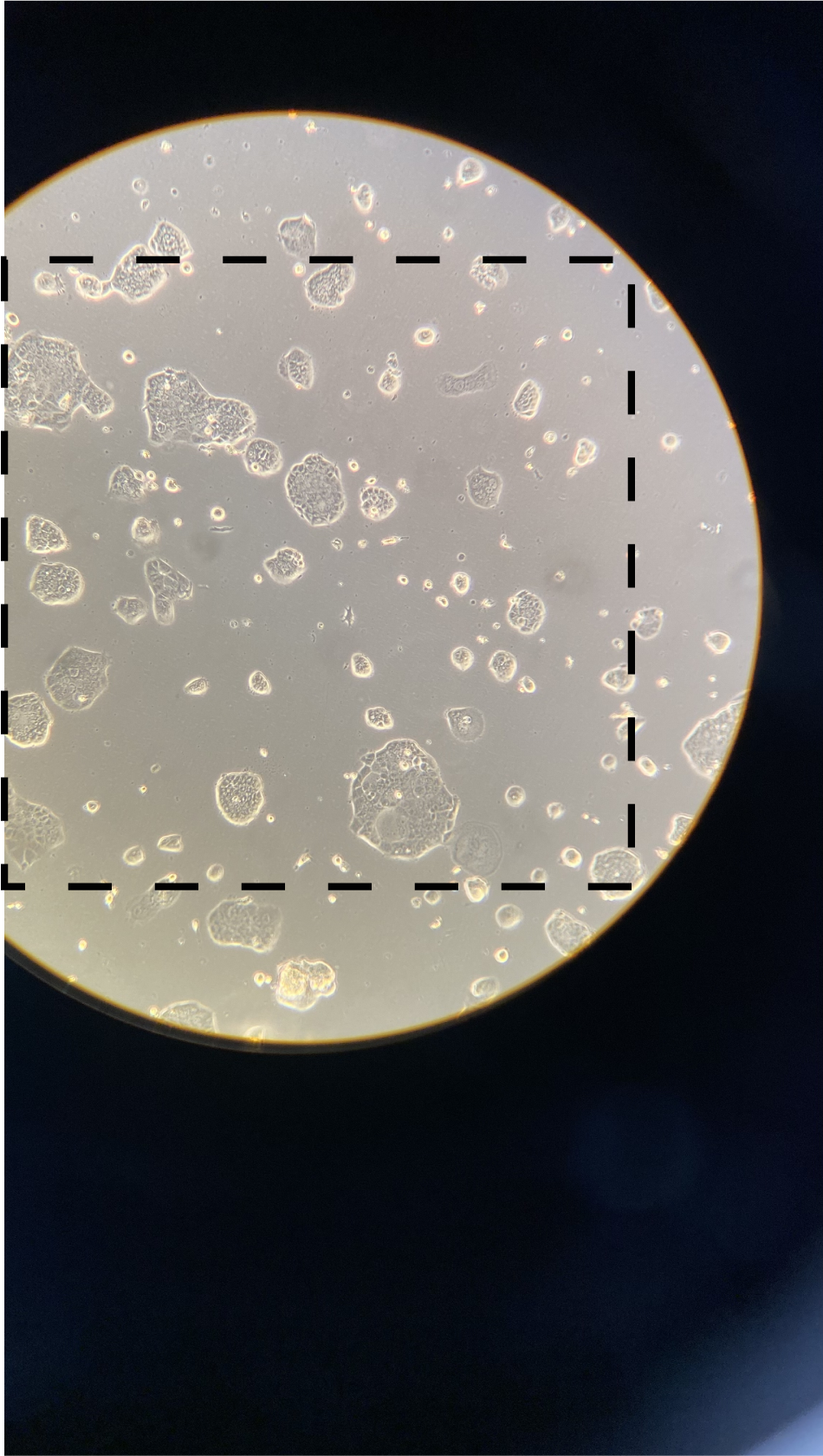

Supplement: Supplementary file 10 — Source data Fig. 5 [file 44318_2024_219_MOESM10_ESM.zip › Figure 5/5J/EFM19 shEFHD1 with crop.tif]

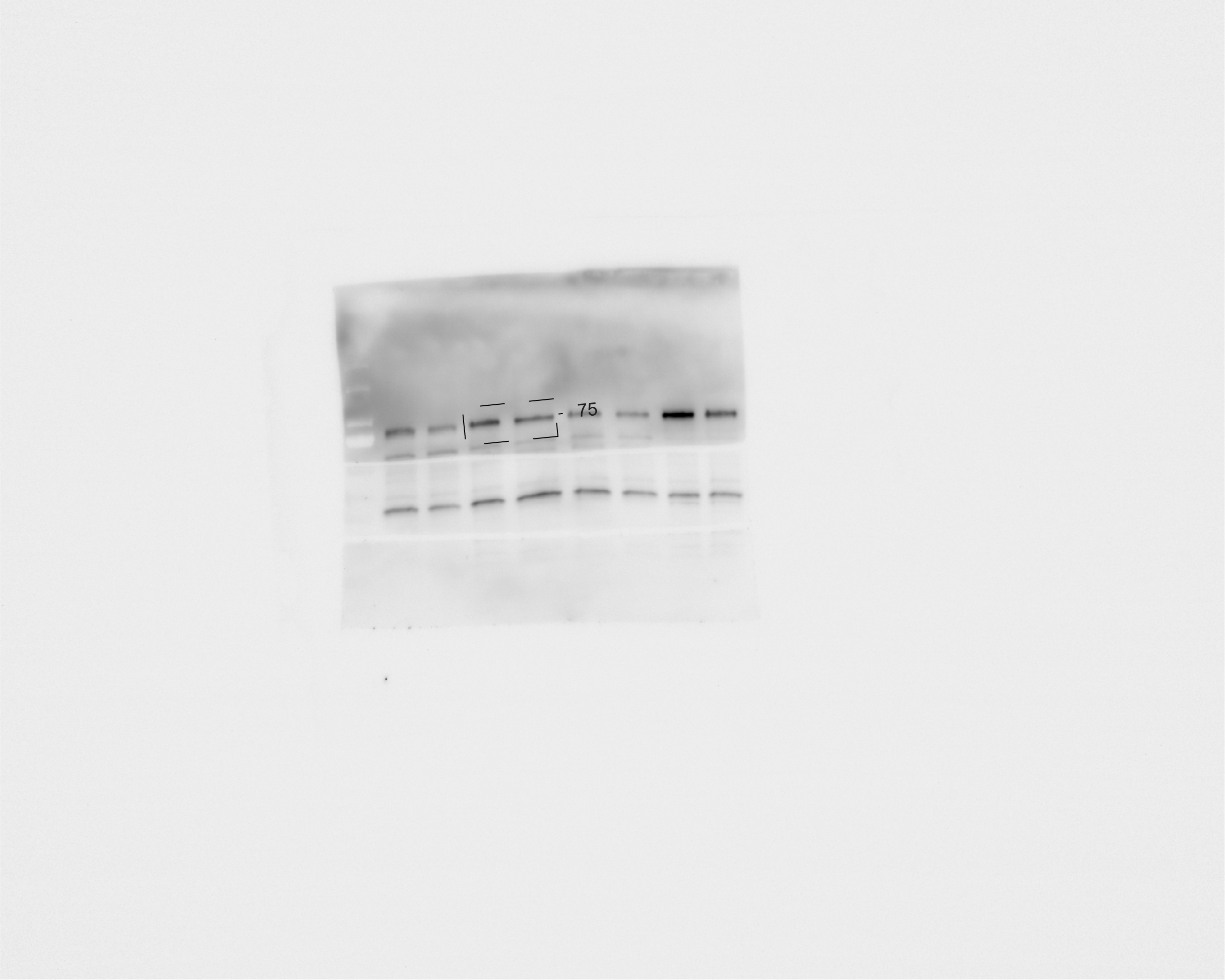

Supplement: Supplementary file 10 — Source data Fig. 5 [file 44318_2024_219_MOESM10_ESM.zip › Figure 5/5J/GRP75_EFM19.tif]

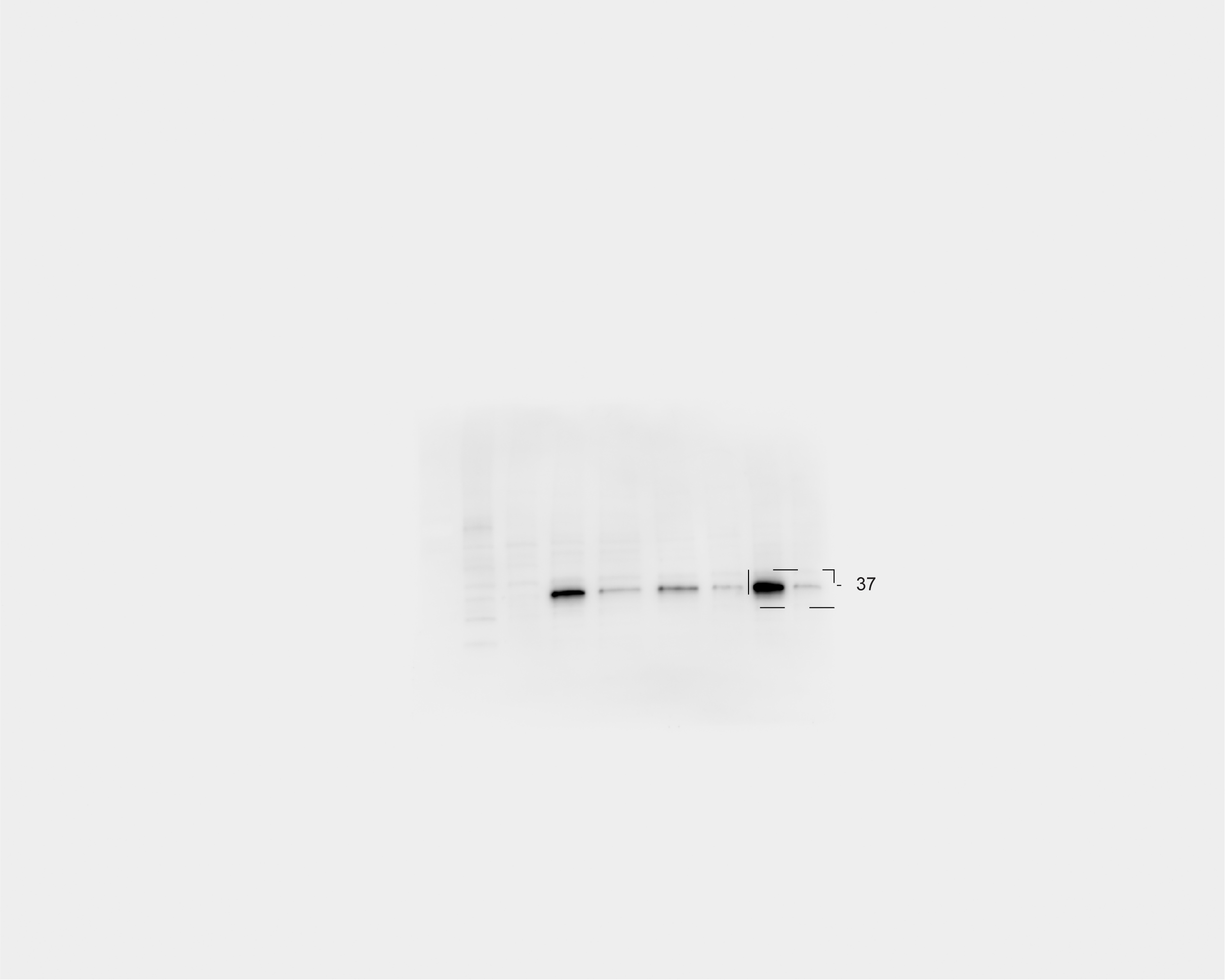

Supplement: Supplementary file 10 — Source data Fig. 5 [file 44318_2024_219_MOESM10_ESM.zip › Figure 5/5K/EFHD1_HCC1500.tif]

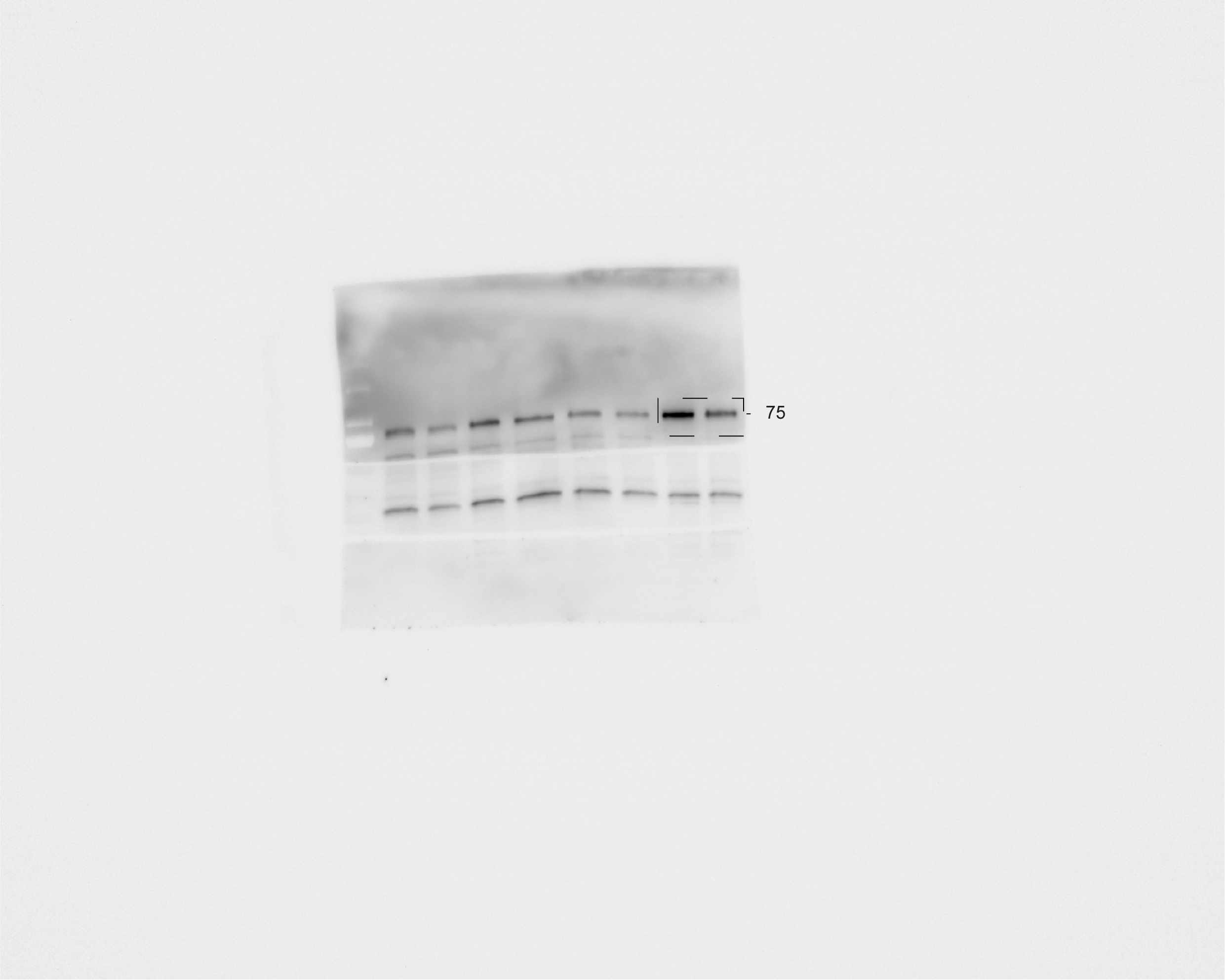

Supplement: Supplementary file 10 — Source data Fig. 5 [file 44318_2024_219_MOESM10_ESM.zip › Figure 5/5K/GRP75_HCC1500.tif]

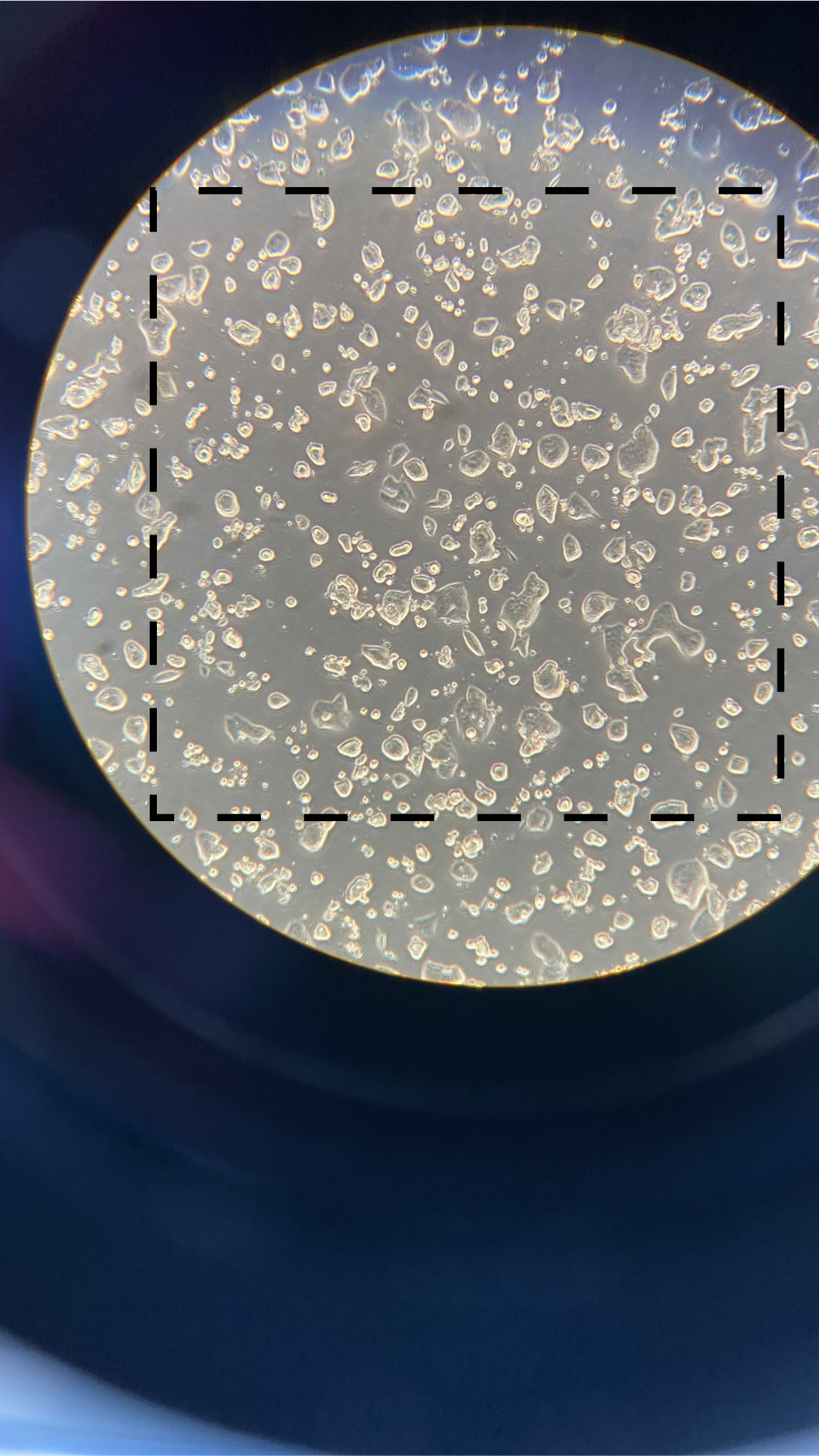

Supplement: Supplementary file 10 — Source data Fig. 5 [file 44318_2024_219_MOESM10_ESM.zip › Figure 5/5K/HCC1500 pLKO with crop.tif]

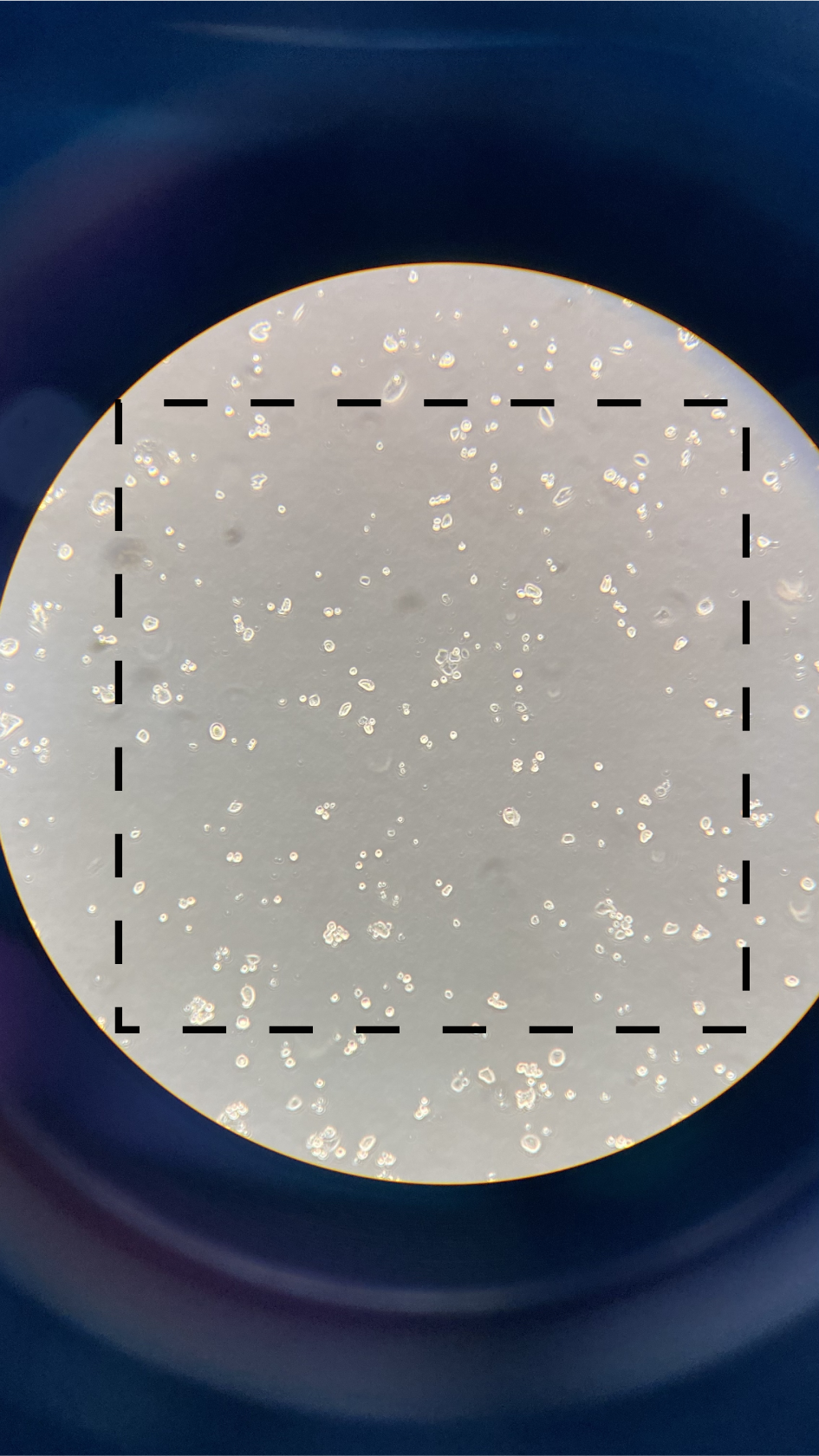

Supplement: Supplementary file 10 — Source data Fig. 5 [file 44318_2024_219_MOESM10_ESM.zip › Figure 5/5K/HCC1500 shEFHD1 with crop.tif]

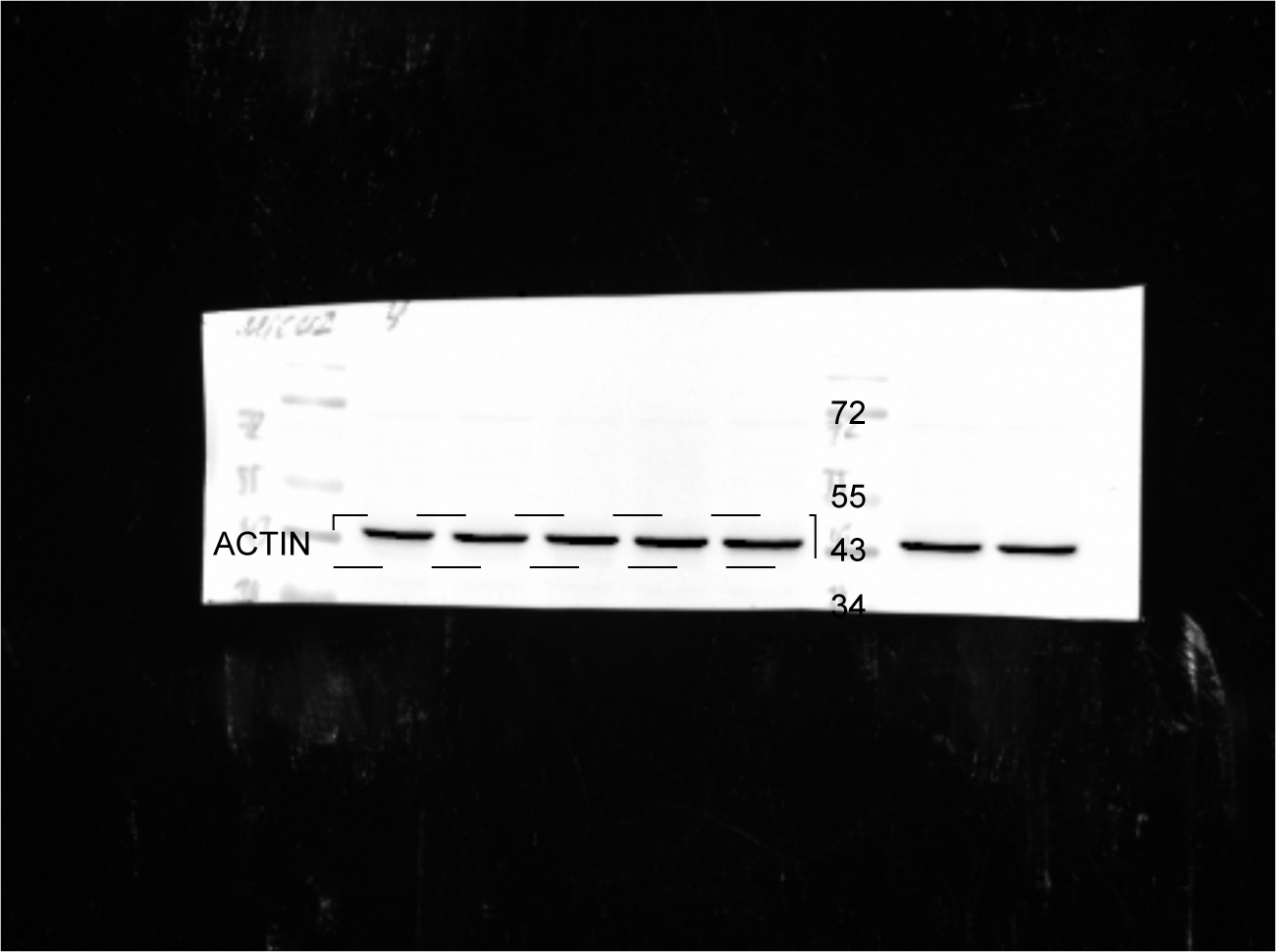

Supplement: Supplementary file 11 — Source data Fig. 6 [file 44318_2024_219_MOESM11_ESM.zip › Figure 6/6A/ACTIN_MW.tif]

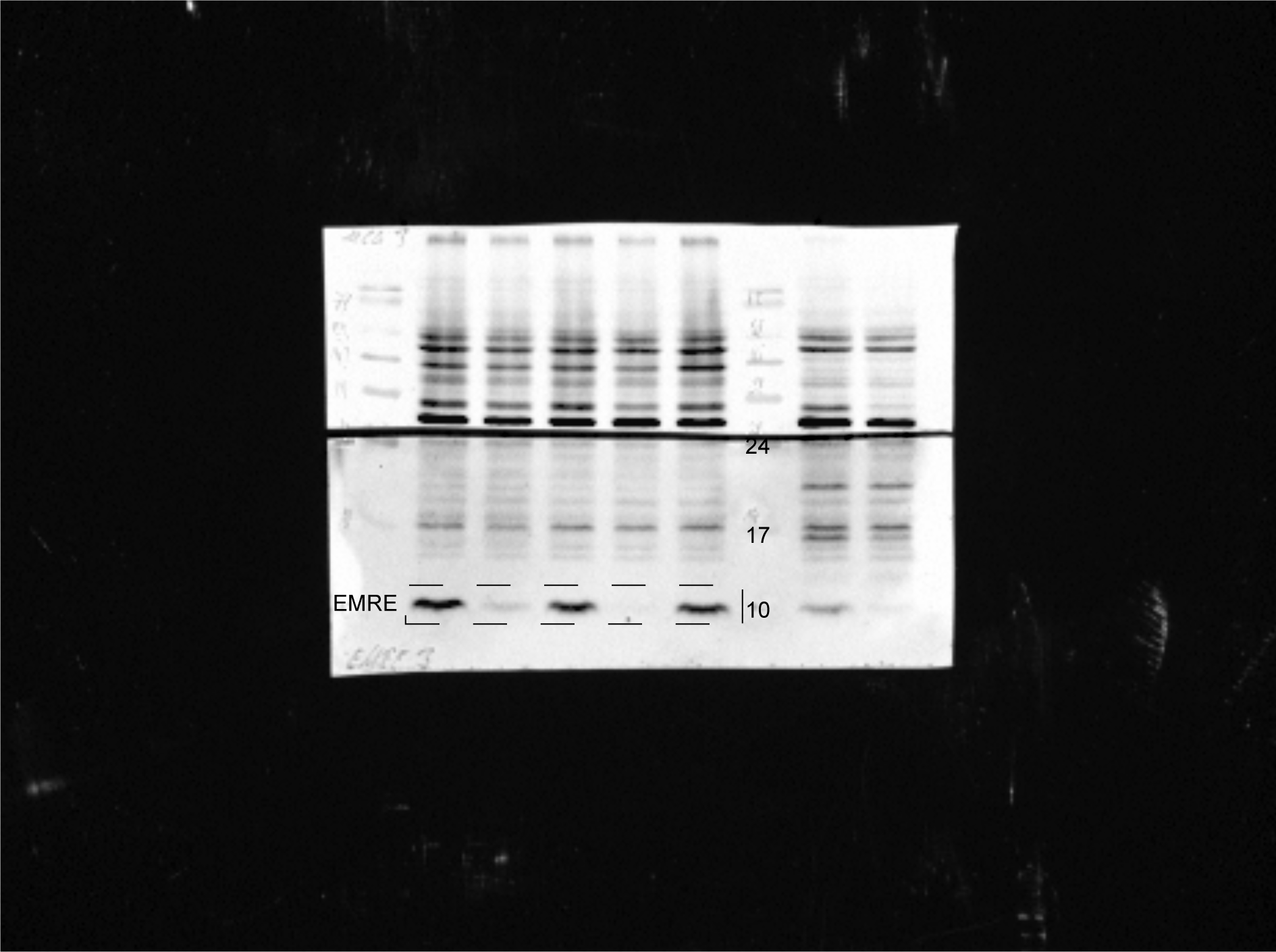

Supplement: Supplementary file 11 — Source data Fig. 6 [file 44318_2024_219_MOESM11_ESM.zip › Figure 6/6A/EMRE_MW.tif]

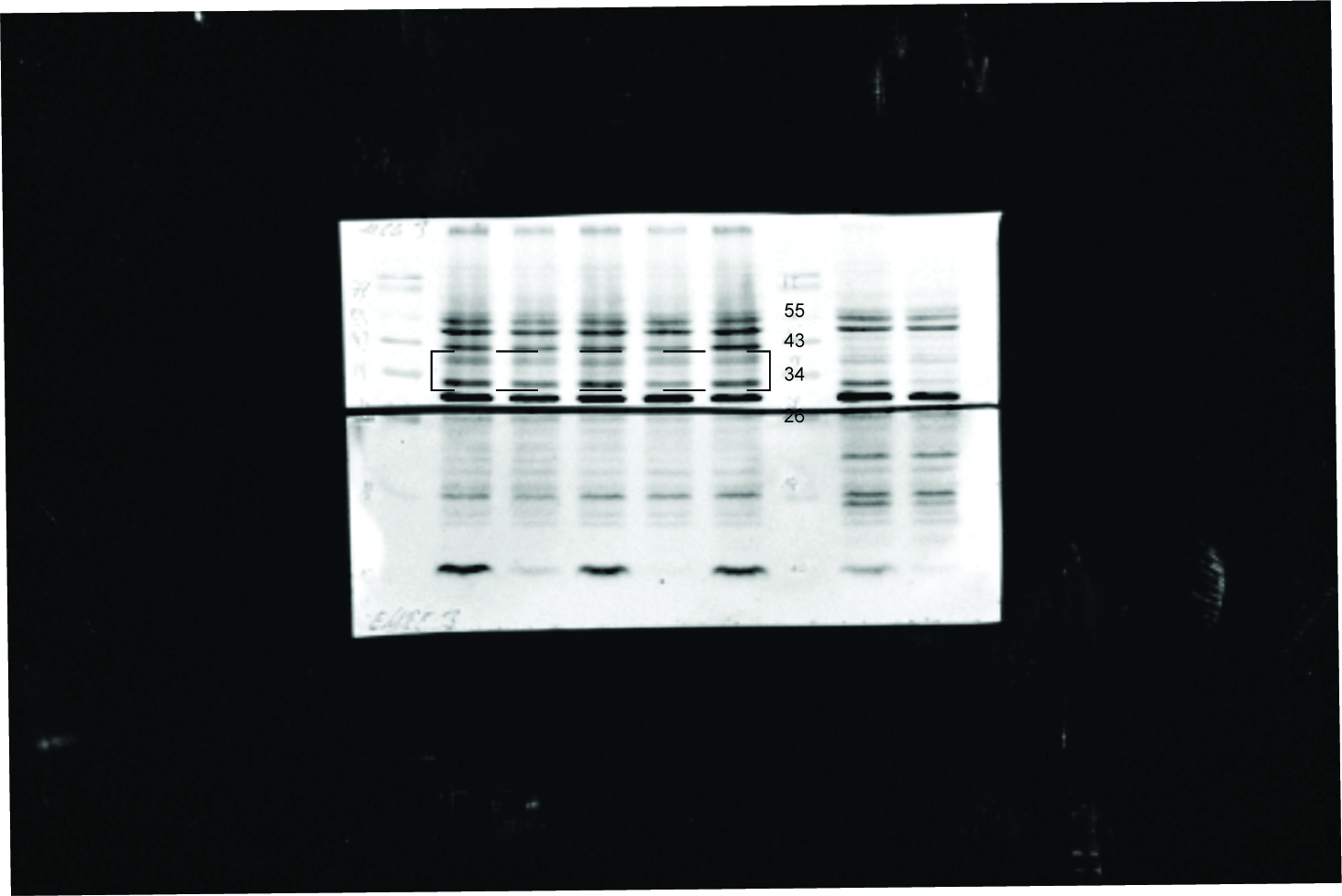

Supplement: Supplementary file 11 — Source data Fig. 6 [file 44318_2024_219_MOESM11_ESM.zip › Figure 6/6A/MCU_MW.tif]

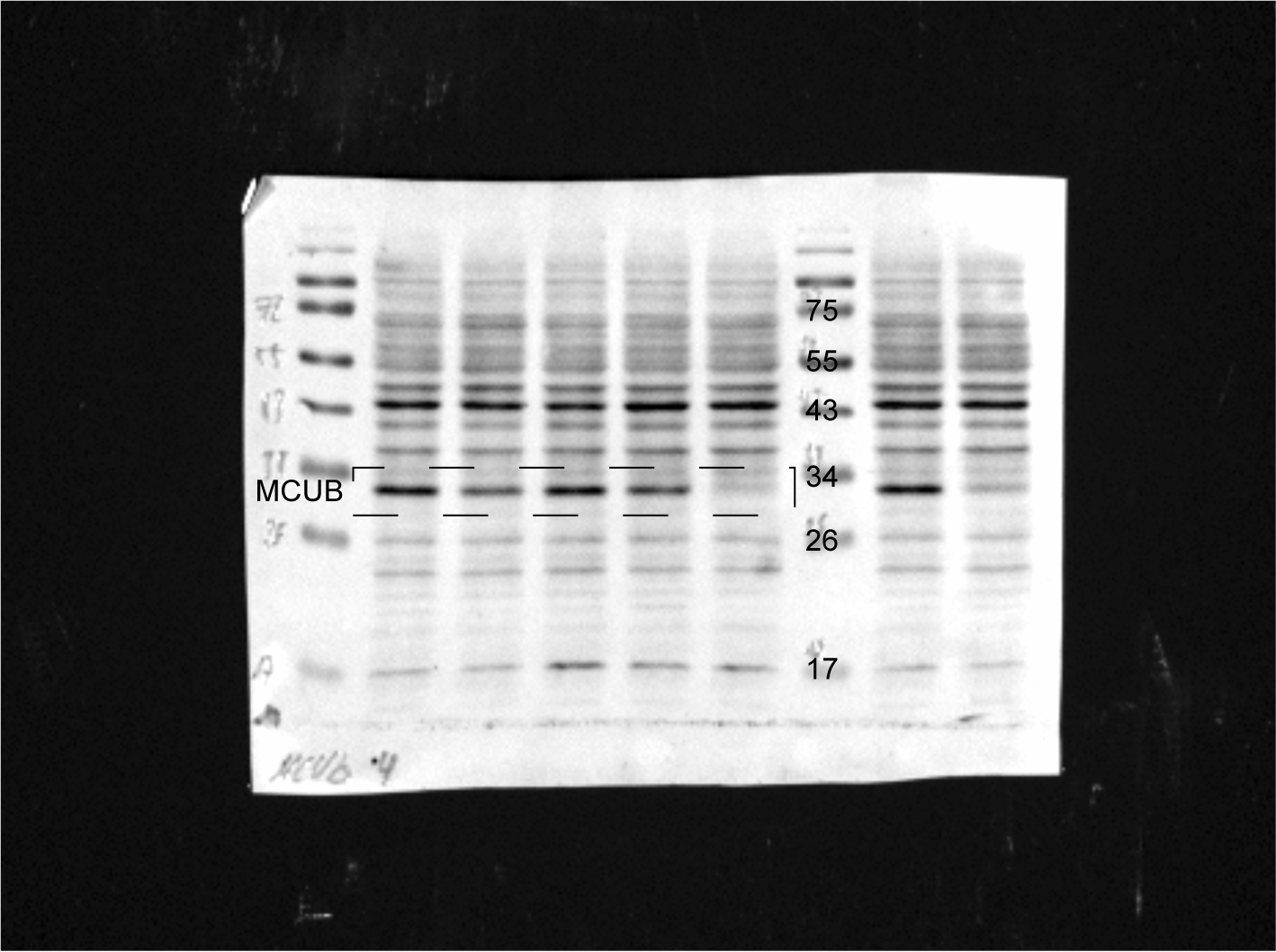

Supplement: Supplementary file 11 — Source data Fig. 6 [file 44318_2024_219_MOESM11_ESM.zip › Figure 6/6A/MCUB_MW.tif]

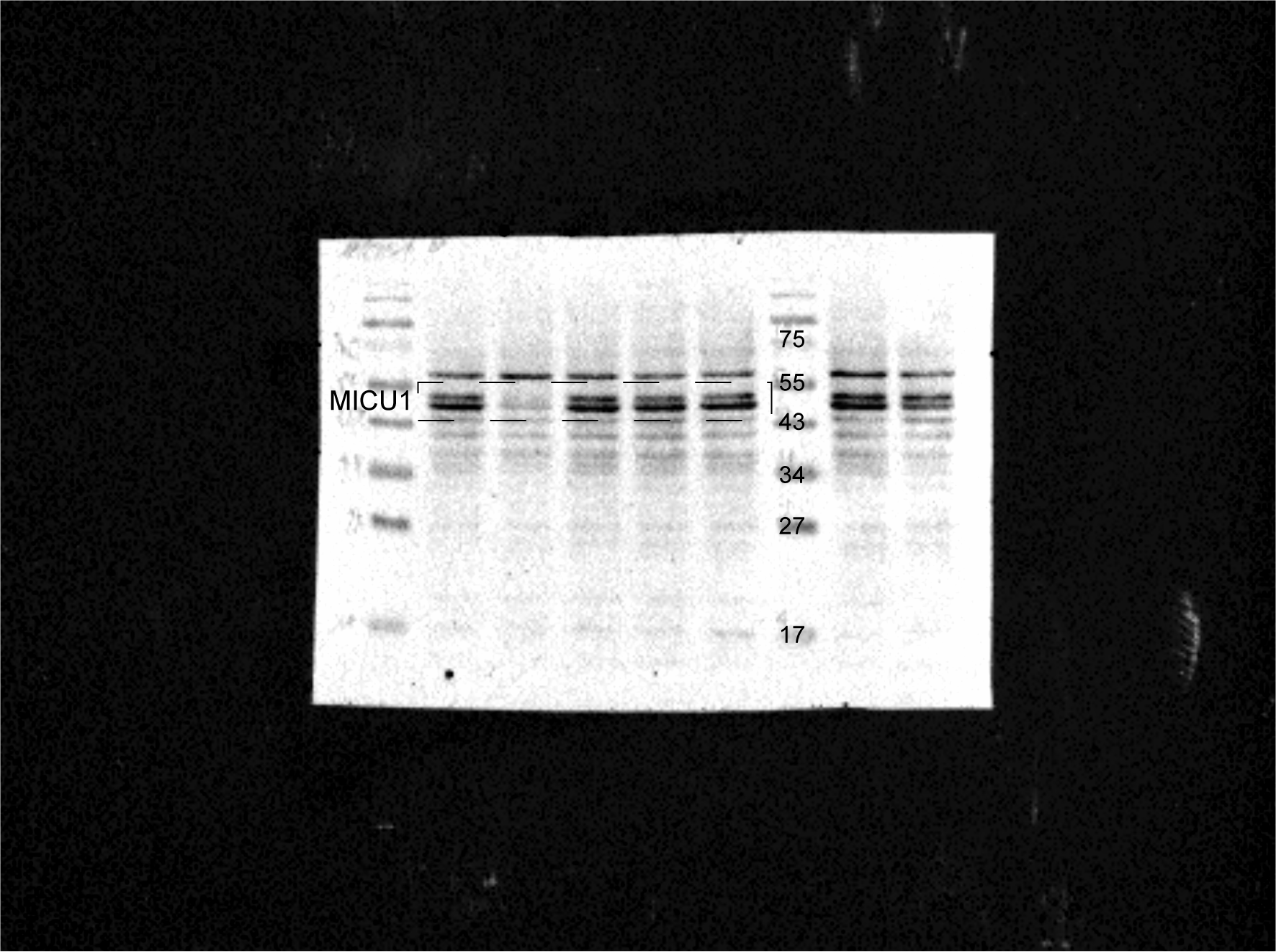

Supplement: Supplementary file 11 — Source data Fig. 6 [file 44318_2024_219_MOESM11_ESM.zip › Figure 6/6A/MICU1_MW.tif]

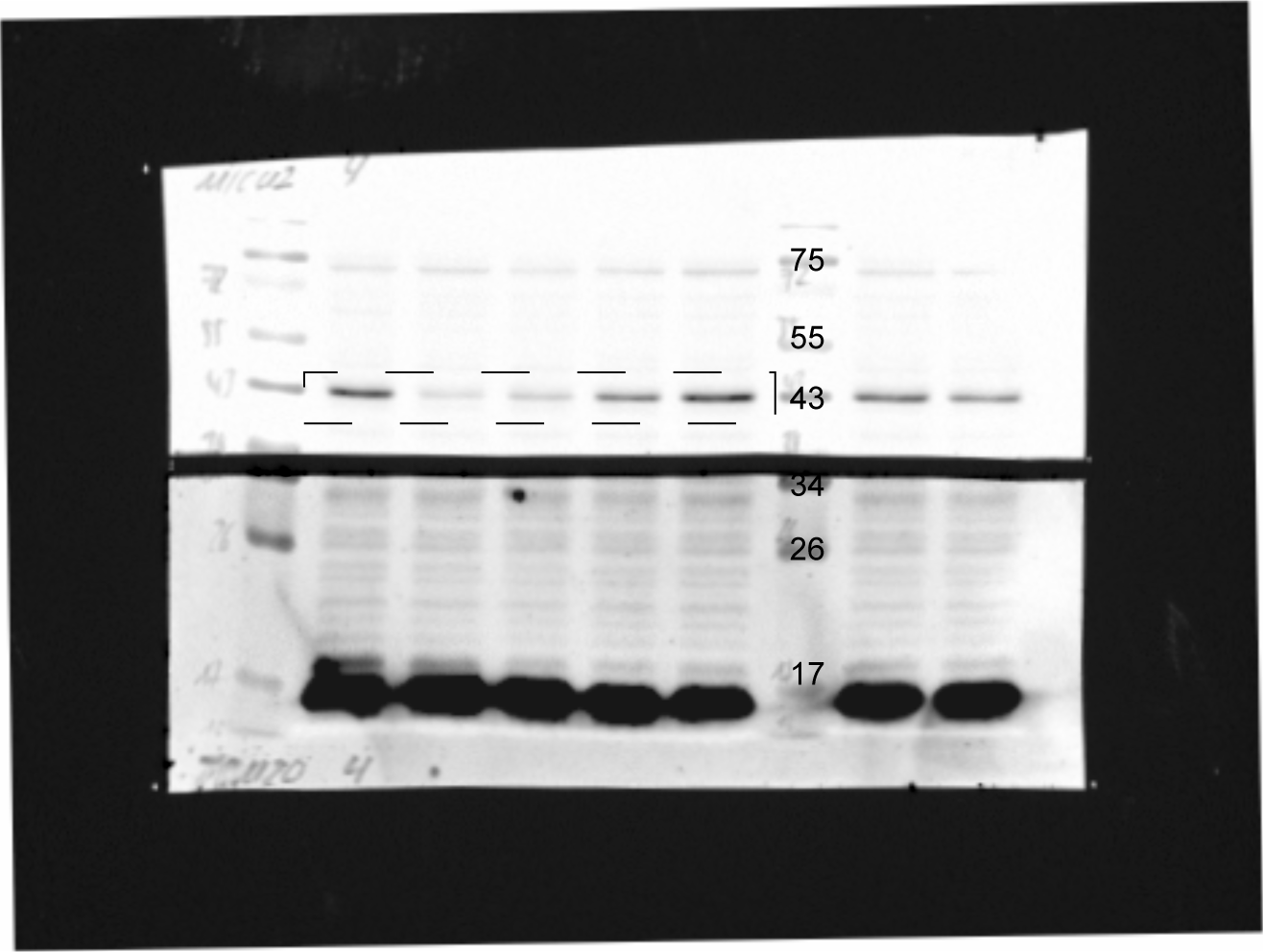

Supplement: Supplementary file 11 — Source data Fig. 6 [file 44318_2024_219_MOESM11_ESM.zip › Figure 6/6A/MICU2_MW.tif]

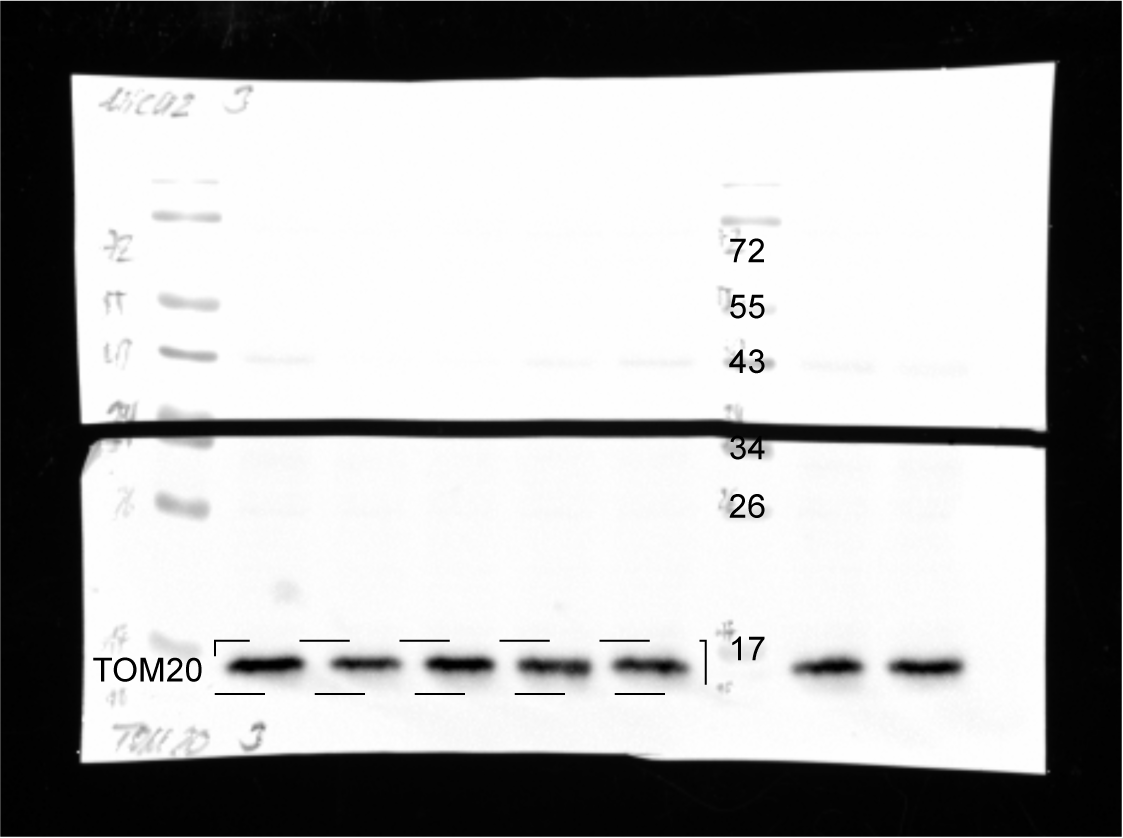

Supplement: Supplementary file 11 — Source data Fig. 6 [file 44318_2024_219_MOESM11_ESM.zip › Figure 6/6A/TOM20_MW.tif]

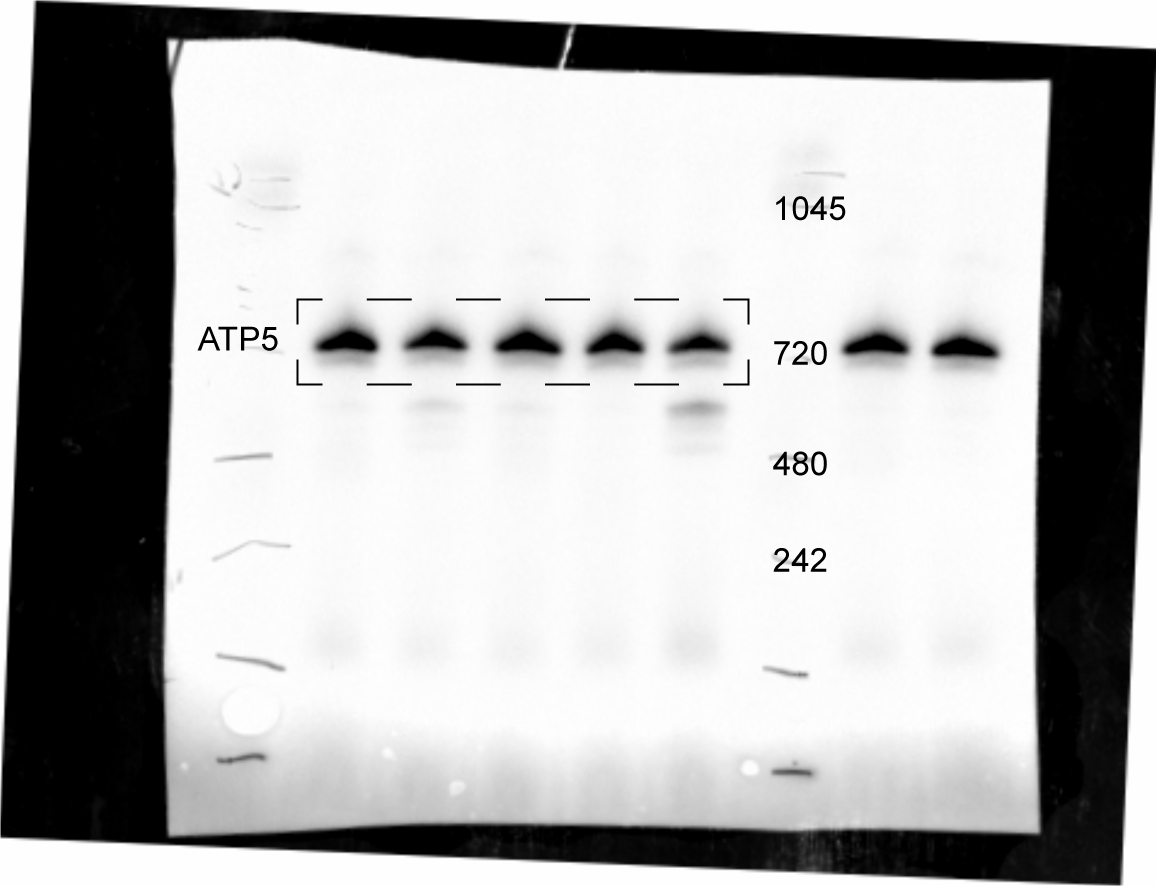

Supplement: Supplementary file 11 — Source data Fig. 6 [file 44318_2024_219_MOESM11_ESM.zip › Figure 6/6G/ATP5A_MW.tif]

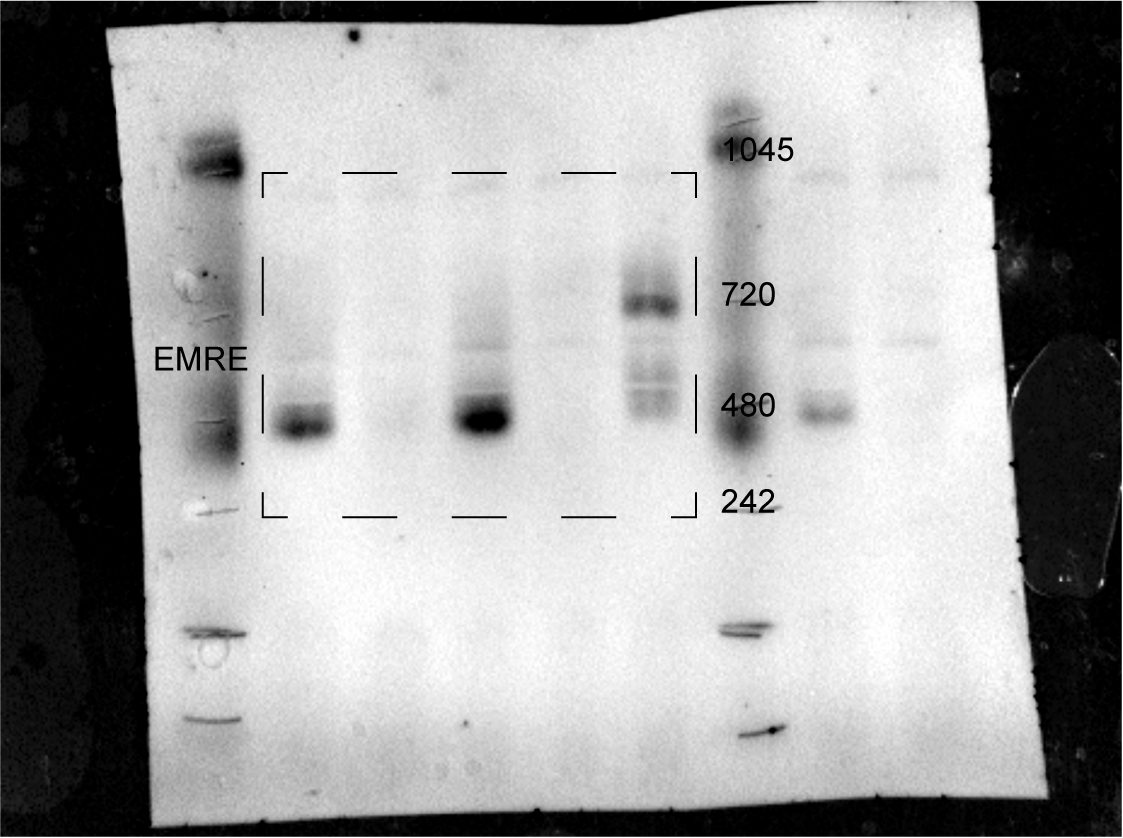

Supplement: Supplementary file 11 — Source data Fig. 6 [file 44318_2024_219_MOESM11_ESM.zip › Figure 6/6G/EMRE_MW.tif]

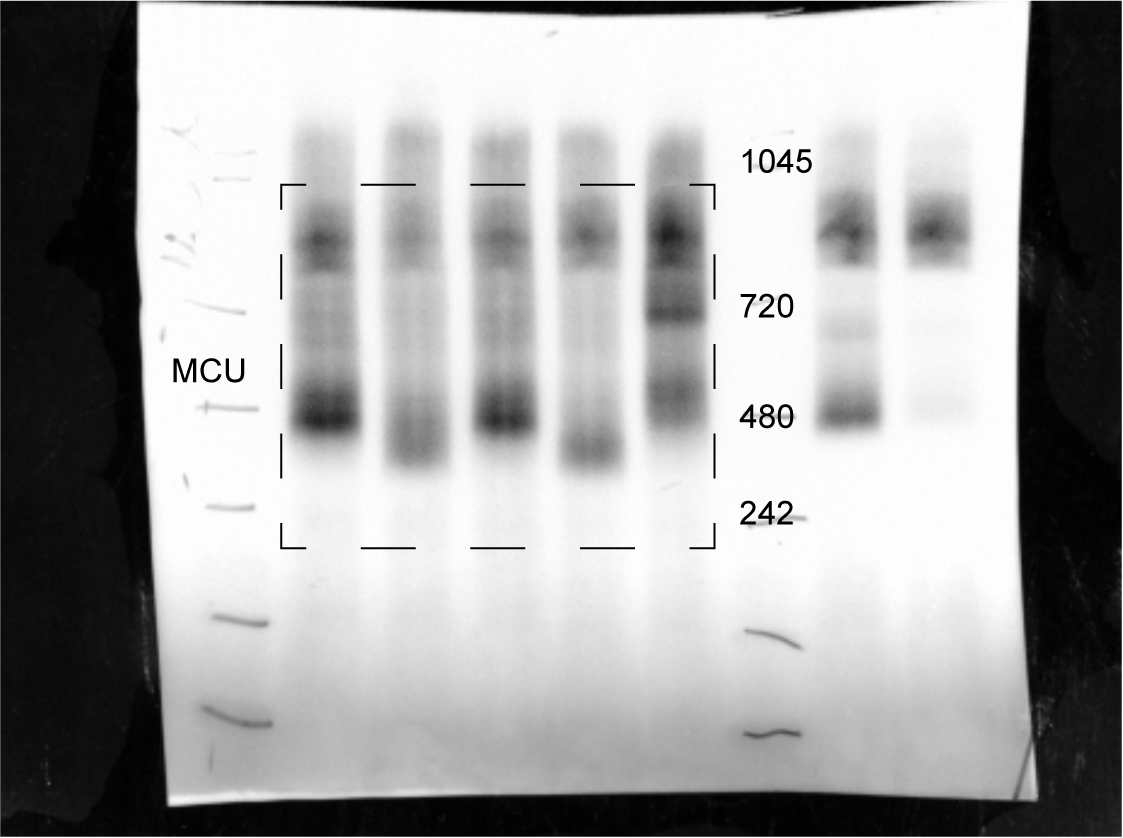

Supplement: Supplementary file 11 — Source data Fig. 6 [file 44318_2024_219_MOESM11_ESM.zip › Figure 6/6G/MCU_MW.tif]

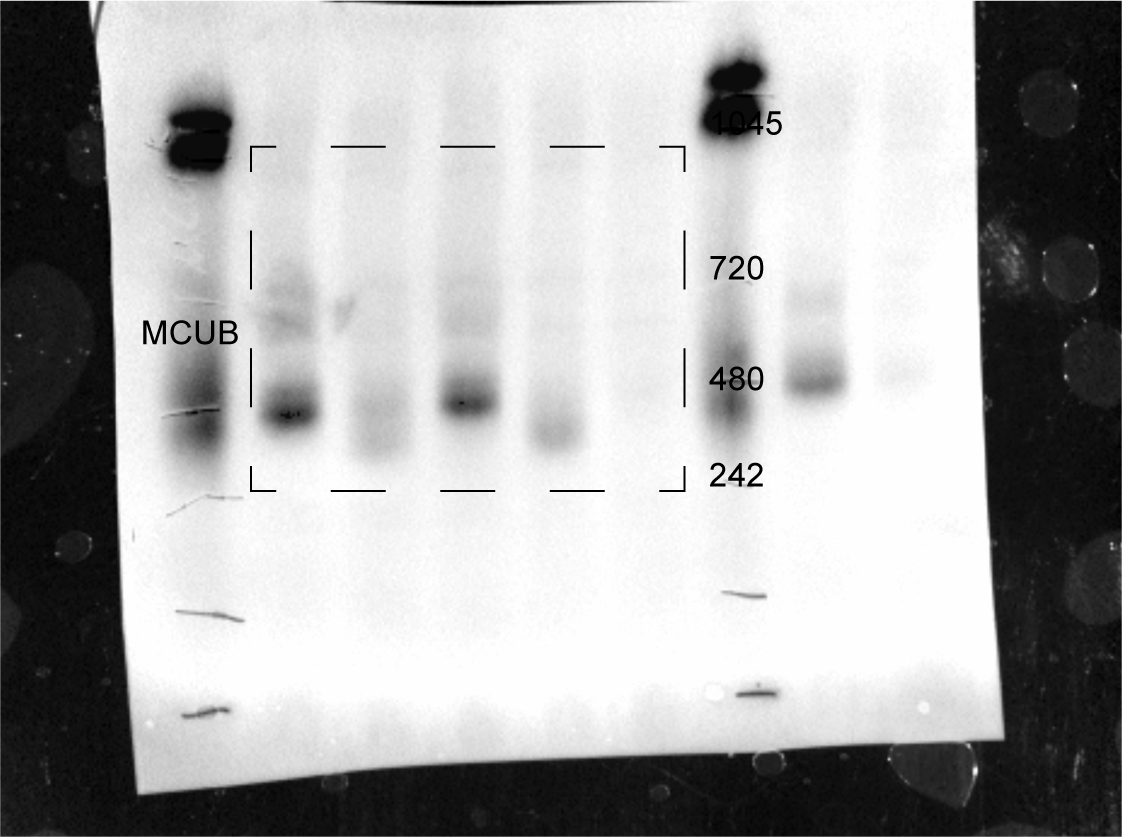

Supplement: Supplementary file 11 — Source data Fig. 6 [file 44318_2024_219_MOESM11_ESM.zip › Figure 6/6G/MCUB_MW.tif]

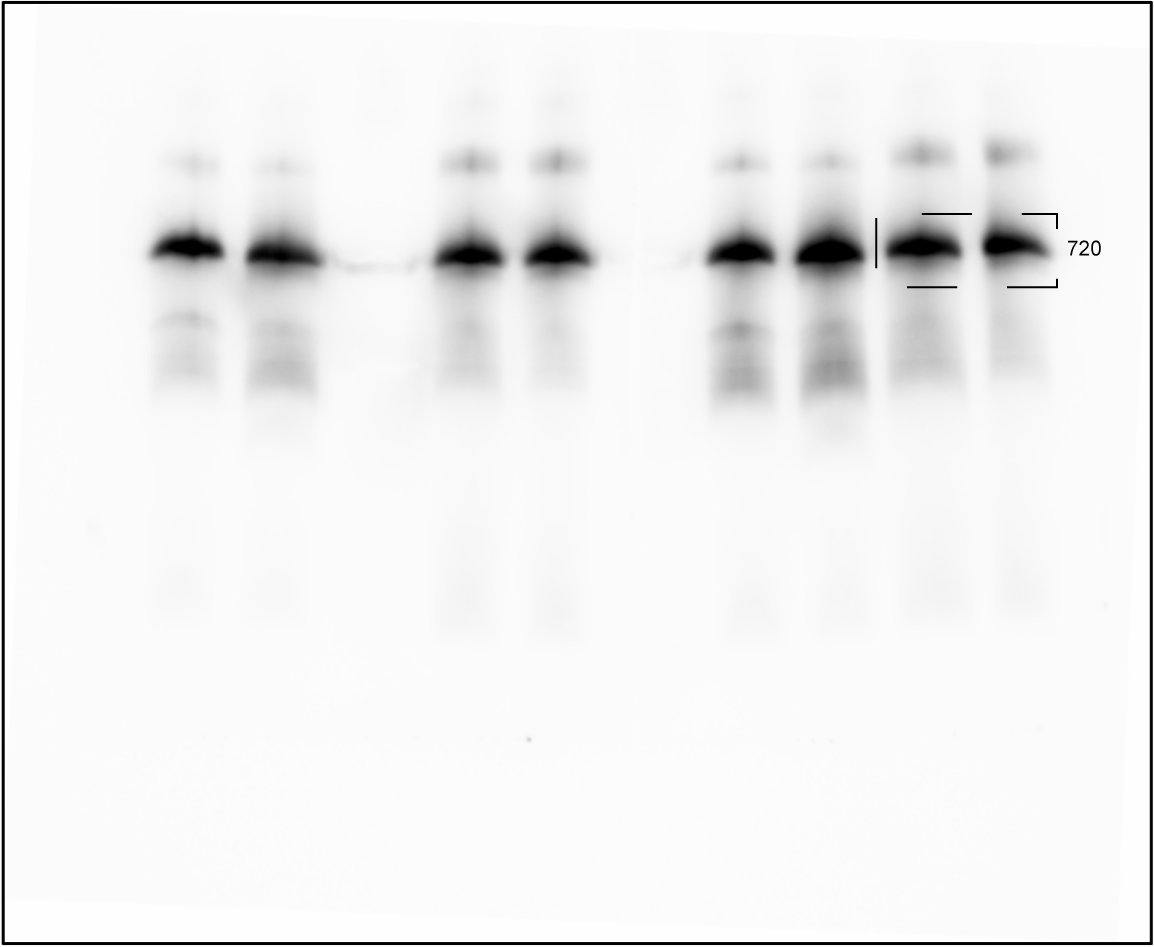

Supplement: Supplementary file 11 — Source data Fig. 6 [file 44318_2024_219_MOESM11_ESM.zip › Figure 6/6H/ATP5A_HEK_MW.tif]

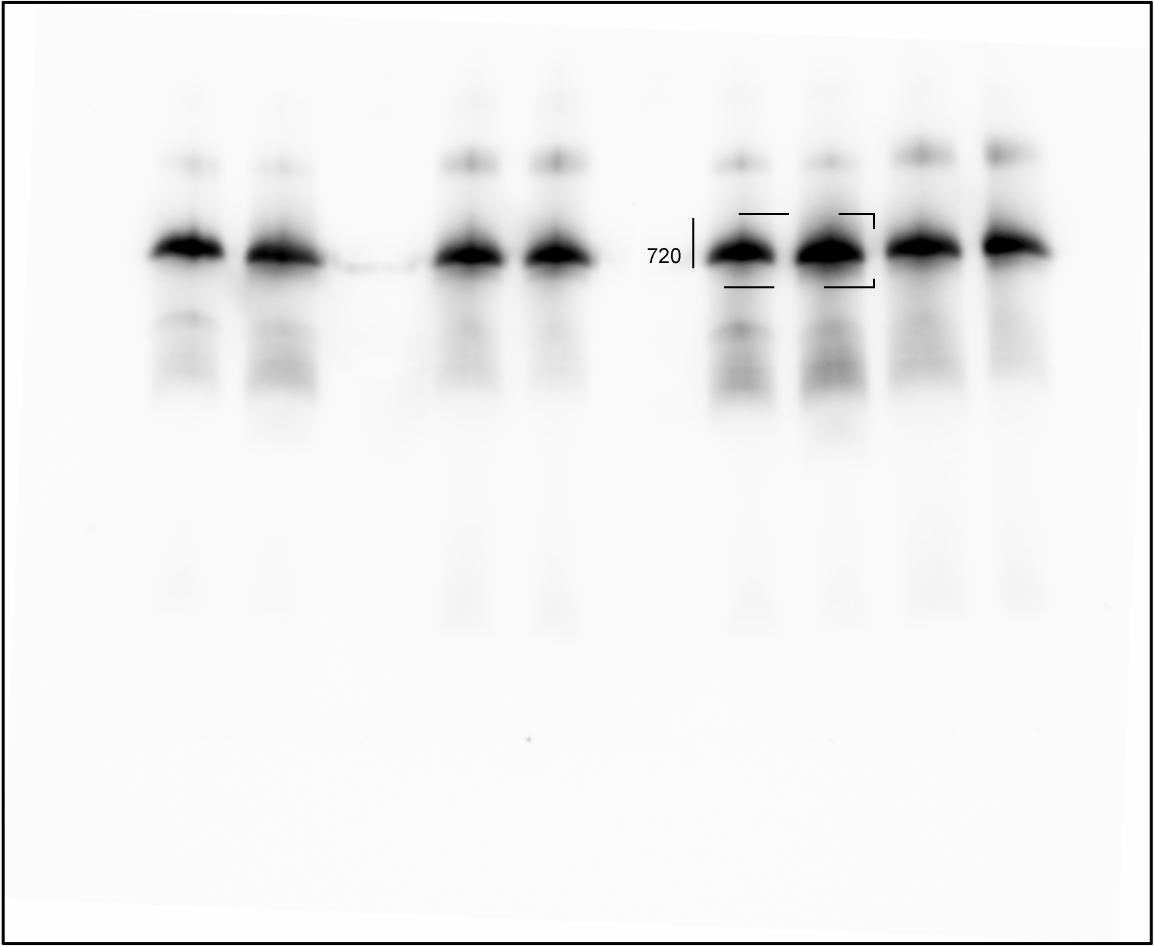

Supplement: Supplementary file 11 — Source data Fig. 6 [file 44318_2024_219_MOESM11_ESM.zip › Figure 6/6H/ATP5A_HeLa_MW.tif]

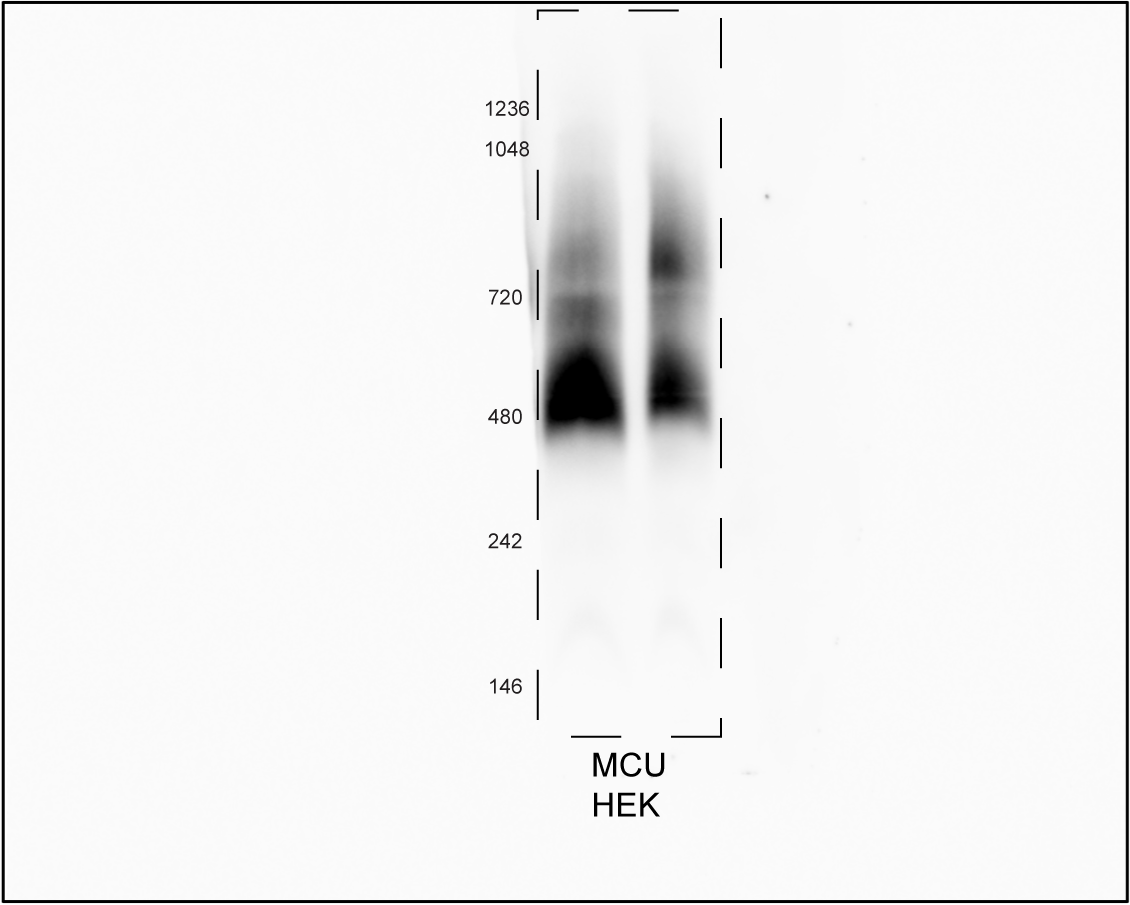

Supplement: Supplementary file 11 — Source data Fig. 6 [file 44318_2024_219_MOESM11_ESM.zip › Figure 6/6H/MCU_HEK.tif]

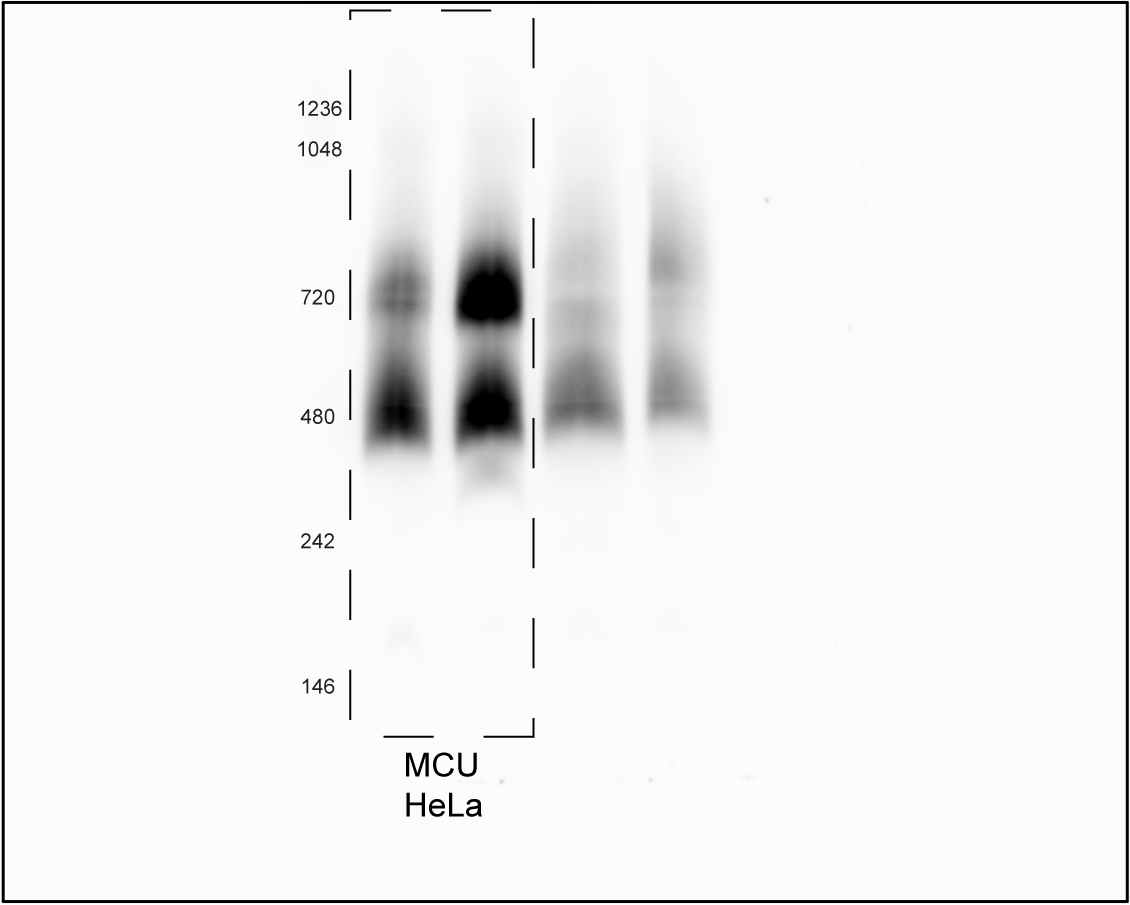

Supplement: Supplementary file 11 — Source data Fig. 6 [file 44318_2024_219_MOESM11_ESM.zip › Figure 6/6H/MCU_HeLa_MW.tif]

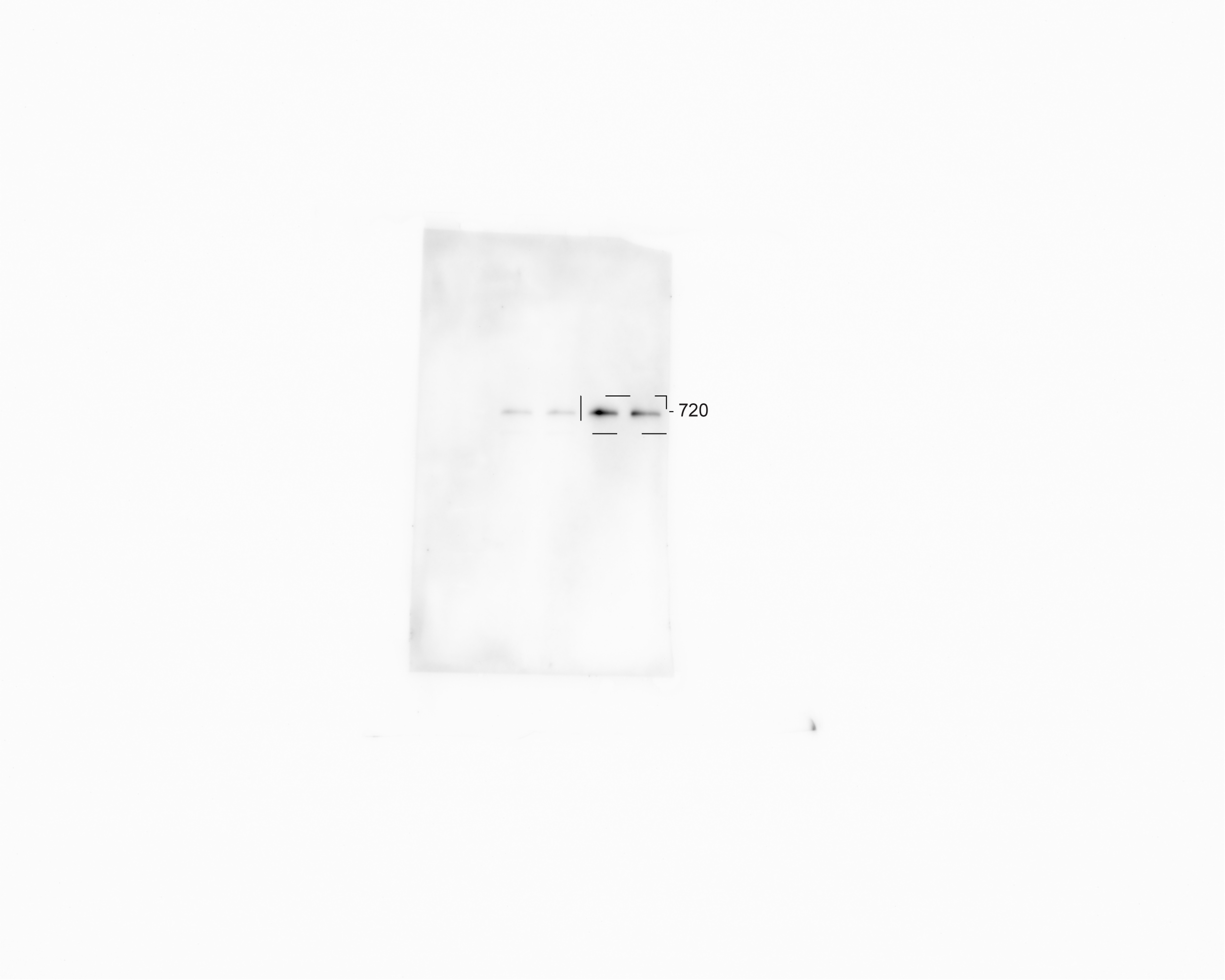

Supplement: Supplementary file 11 — Source data Fig. 6 [file 44318_2024_219_MOESM11_ESM.zip › Figure 6/6I/ATP5A_MW.tif]

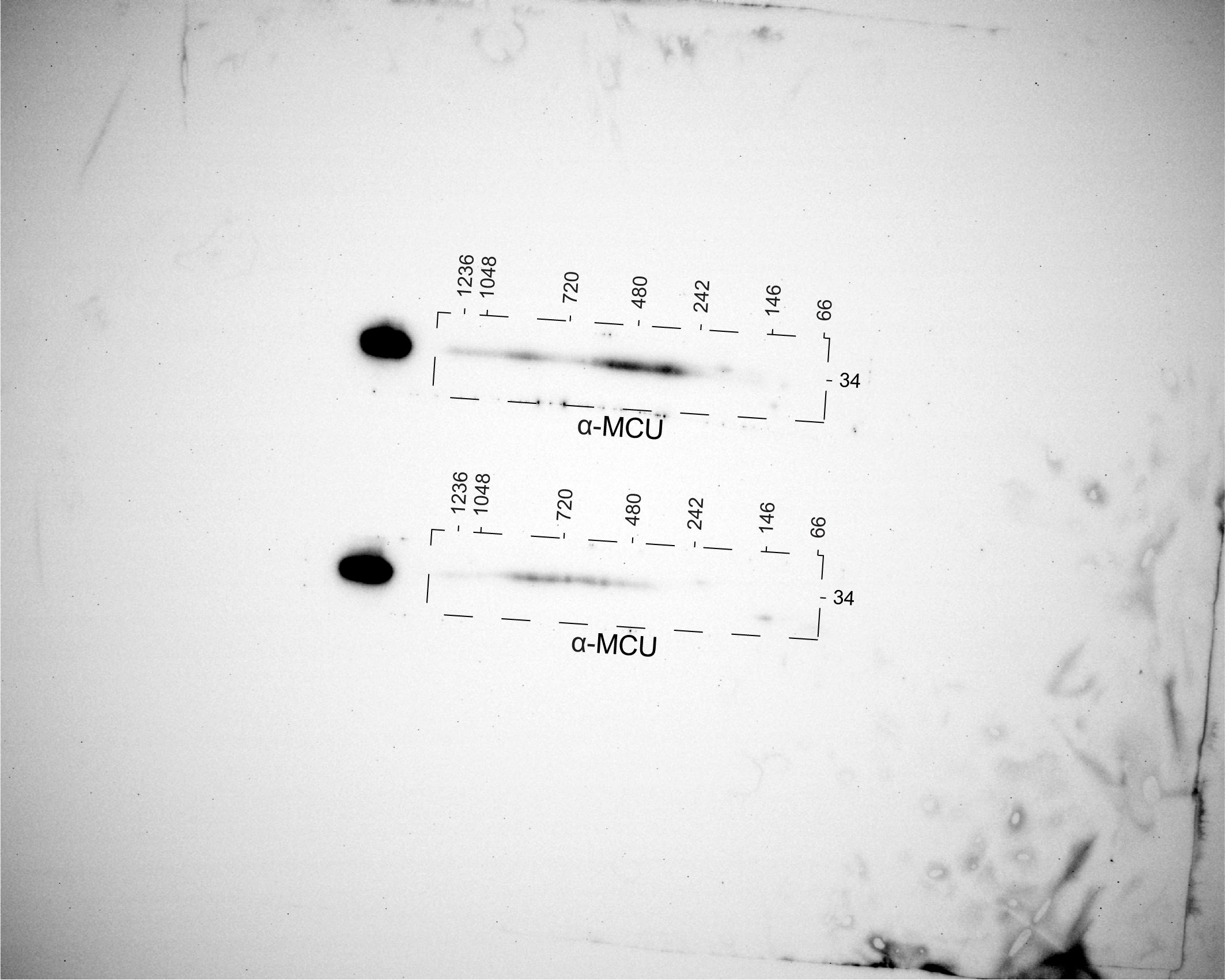

Supplement: Supplementary file 11 — Source data Fig. 6 [file 44318_2024_219_MOESM11_ESM.zip › Figure 6/6I/MCU_2D_MW.tif]

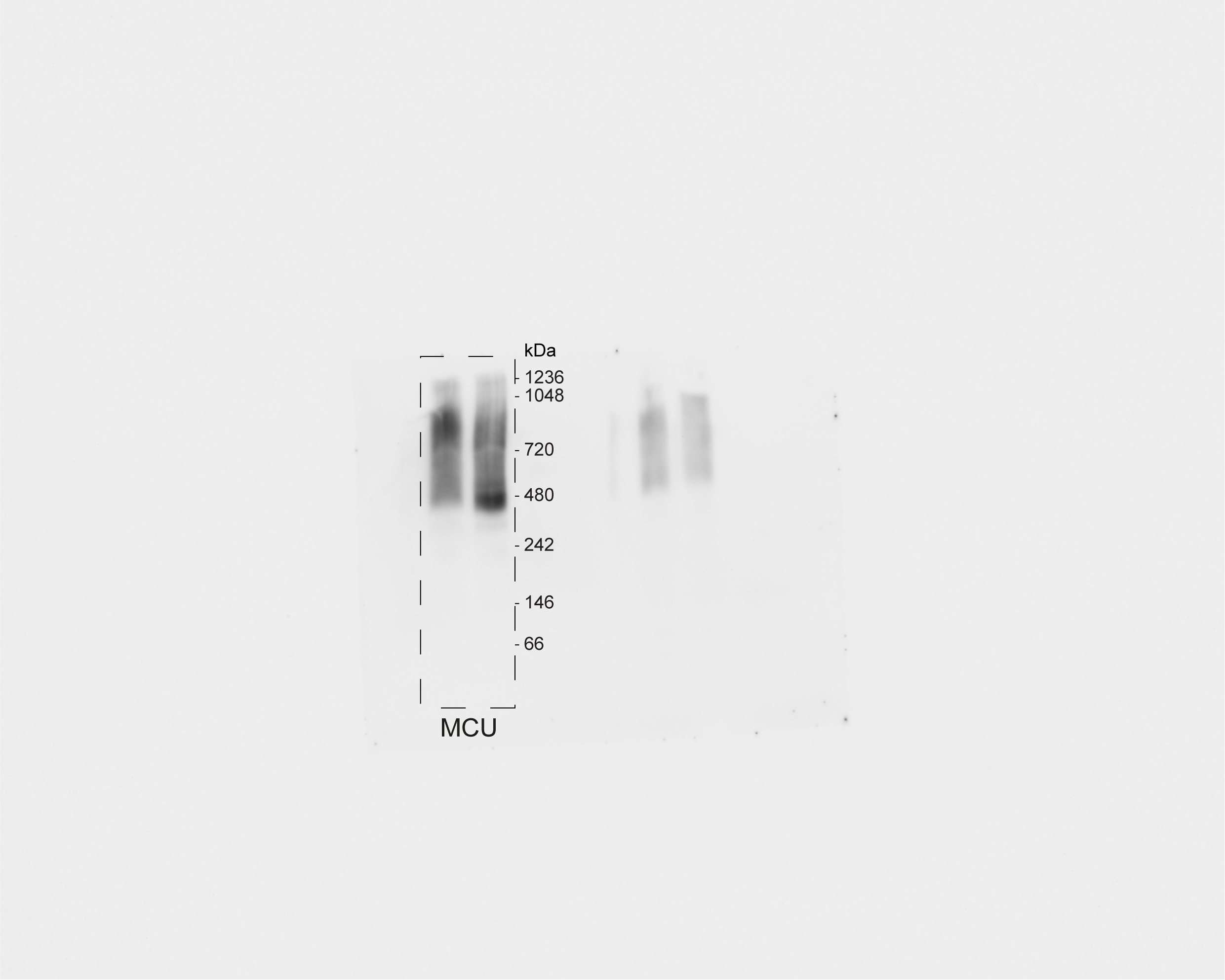

Supplement: Supplementary file 11 — Source data Fig. 6 [file 44318_2024_219_MOESM11_ESM.zip › Figure 6/6I/MCU_MW.tif]
