# Supplementary material for: Systematic mapping of mitochondrial calcium uniporter channel (MCUC)-mediated calcium signaling networks
Source: EMBO J. 2024 Sep 11;43(21):22. doi: 10.1038/s44318-024-00219-w (PMC11535509; doi:10.1038/s44318-024-00219-w)
Supplement: Supplementary file 12 — Expanded View Figures [file 44318_2024_219_MOESM12_ESM.pdf]

Expanded View Figures

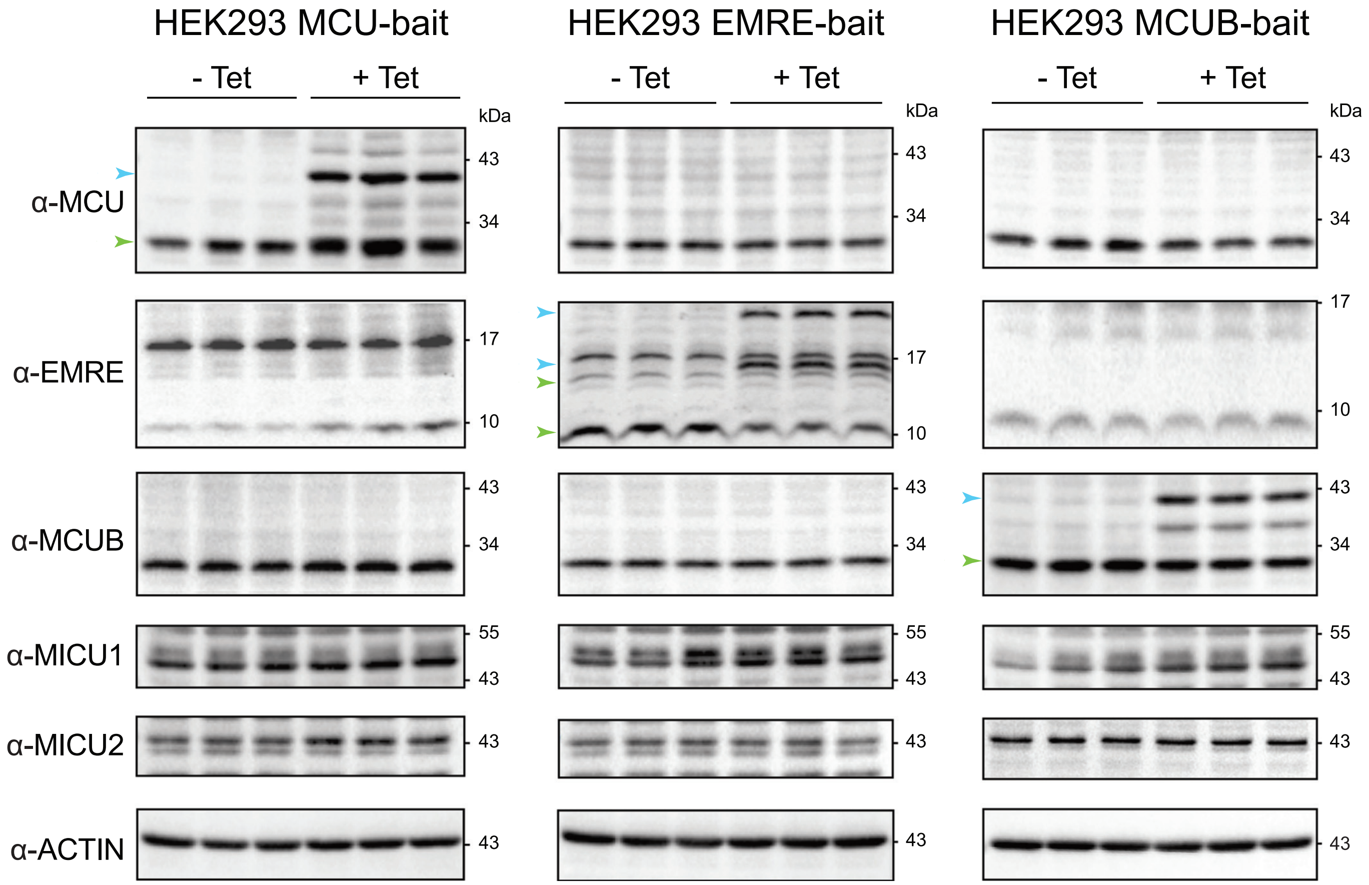

Figure EV1. Expression of MCUC components before and after bait induction.

Immunoblot analysis of MCU, EMRE, MCUB, MICU1, MICU2 and ACTIN (loading control) in whole cell lysates from Flp-In T-REx HEK293 cell lines before (-Tet) and after (+Tet) tetracycline-driven expression of each bait. Immunoblots of MCU from MCU-bait cells, EMRE from EMRE-bait cells and MCUB from MCUB-bait cells were re-used from Fig. 2A. Refer to quantification in Fig. 2A.

 © The Author(s)

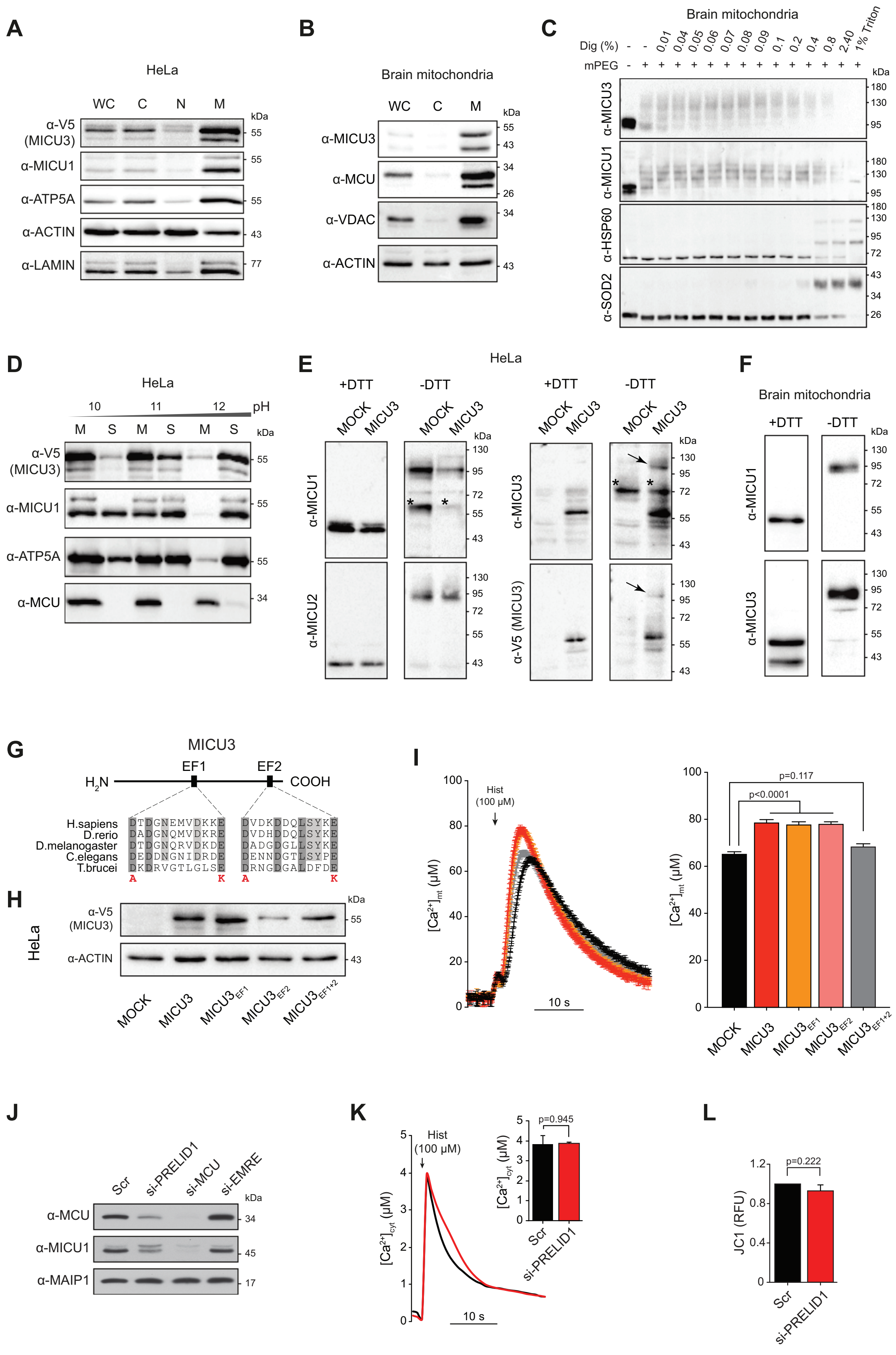
A
HeLa
WC C N M
α-V5 (MICU3)
α-MICU1
α-ATP5A
α-ACTIN
α-LAMIN
kDa 55 55 55 43 77
B
Brain mitochondria
WC C M
α-MICU3
α-MCU
α-VDAC
α-ACTIN
kDa 55 43 34 26 34 43
C
Brain mitochondria
Dig (%) - - 0.01 0.04 0.05 0.06 0.07 0.08 0.09 0.1 0.2 0.4 0.8 2.40 1% Triton
mPEG - + + + + + + + + + + + + + +
α-MICU3
α-MICU1
α-HSP60
α-SOD2
kDa 180 130 95 180 130 95 180 130 95 72 43 34 26
D
HeLa
10 11 12 pH
M S M S M S
α-V5 (MICU3)
α-MICU1
α-ATP5A
α-MCU
kDa 55 55 55 34
E
HeLa
+DTT -DTT +DTT -DTT
MOCK MICU3 MOCK MICU3 MOCK MICU3 MOCK MICU3
α-MICU1
α-MICU2
α-MICU3
α-V5 (MICU3)
kDa 130 95 72 55 43
F
Brain mitochondria
+DTT -DTT
α-MICU1
α-MICU3
kDa 130 95 72 55 43
G
MICU3
H2N EF1 EF2 COOH
H.sapiens
D.rerio
D.melanogaster
C.elegans
T.brucei
DTDGNEMVDKKE DVDKDDQLSYKE
DADGNQMVDKRE DVDHDDQLSYKE
DTDGNQRVDKDE DADGDGLLSYKE
DEDDNGNIDRDE DENNDGTLSYPE
DKDRVGTLGLSE DRNGDGALDFDE
A K A K
H
HeLa
α-V5 (MICU3)
α-ACTIN
kDa 55 43
MOCK MICU3 MICU3EF1 MICU3EF2 MICU3EF1+2
I
Hist (100 μM)
[Ca2+]mt (μM)
10 s
p=0.117
p<0.0001
MOCK MICU3 MICU3EF1 MICU3EF2 MICU3EF1+2
J
Scr si-PRELID1 si-MCU si-EMRE
α-MCU
α-MICU1
α-MAIP1
kDa 34 45 17
K
Hist (100 μM)
[Ca2+]cyt (μM)
10 s
p=0.945
Scr si-PRELID1
L
p=0.222
JC1 (RFU)
Scr si-PRELID1

Figure EV2. MICU3 positively regulates MCU-dependent mitochondrial Ca^{2+} uptake and PRELID1 is required for MCUC stability.

(**A**, **B**) MICU3 is enriched in mitochondria from (**A**) HeLa cells overexpressing MICU3-V5 and (**B**) mouse brain. WC whole cell lysate, C cytosol, N nuclei, M mitochondria. MICU1, ATP5A, MCU, and VDAC are used as markers of mitochondrial proteins, while ACTIN and LAMIN as markers of cytosolic and nuclear proteins, respectively. (**C**) Immunoblot analysis of mitochondria isolated from mouse brain in the presence of increasing concentrations of the membrane-impermeable sulfhydryl group reactive PEG derivative (mPEG, maleimide functionalized polyethylene glycol). MICU1 is used as positive control for IMS proteins, whereas HSP60 and SOD2 for mitochondrial matrix proteins. (**D**) Immunoblot analysis of mitochondrial soluble (S) and membrane (M) fractions isolated from HeLa cells overexpressing MICU3-V5 by alkaline carbonate extraction at pH 10, pH 11, and pH 12. MICU1 and ATP5A (soluble and membrane-associated proteins, respectively), and MCU (integral transmembrane protein) are used as positive controls. (**E**, **F**) MICU3 and MICU1 dimerize through a disulfide bond in mitochondria of (**E**) HeLa cells overexpressing MICU3-V5 compared to control (MOCK) and of (**F**) mouse brain. Immunoblot analysis was performed in both reducing (+DTT) and non-reducing (-DTT) conditions. *Indicates non-specific bands. MICU1/MICU3 dimers are indicated by an arrow. (**G**) Domain structure of MICU3. EF1 and EF2 refer to two evolutionarily conserved EF-hand domains. Amino acid substitution used to generate MICU3 EF-hand mutants are indicated in red ($EF1_{mut}$, D245A and E256K; $EF2_{mut}$, D483A and E494K). (**H**) Immunoblot analysis of exogenous MICU3 detected with an anti-V5 antibody and ACTIN (loading control) in whole cell lysates from HeLa mt-AEQ cells expressing either WT MICU3 (MICU3) or MICU3 mutants in the first ($MICU3_{EF1}$), the second ($MICU3_{EF2}$) or both ($MICU3_{EF1+2}$) EF-hands fused to a C-terminal V5 tag and compared to untransfected control cells (MOCK). (**I**) Average traces and quantification of $[Ca^{2+}]_{mt}$ transients in HeLa mt-AEQ cells expressing either WT or MICU3 mutants in response to histamine (Hist) and compared to control (MOCK). Data represent mean ± SEM ($n = 4$ biological replicates); one-way ANOVA with Dunnett's multiple comparison test. (**J**) Immunoblot analysis of MCU, MICU1 and MAIP1 (loading control) in whole cell lysate from HeLa cells transfected with si-PRELID1 and compared to negative control (Scr). si-MCU and si-EMRE are used as positive controls for MCUC expression and stability. (**K**), Representative traces and quantification of $[Ca^{2+}]_{cyt}$ transients upon histamine (Hist) stimulation in si-PRELID1 and Scr HeLa cells expressing cytosolic aequorin (mean ± SEM; $n = 3$ biological replicates); Student's *t* test. (**L**) JC1-based quantification of mitochondrial membrane potential upon PRELID1 knockdown in HeLa cells (RFU, relative fluorescence unit), (mean ± SEM; $n = 3$ biological replicates); Student's *t* test. Refer to Fig. 4.

 © The Author(s)

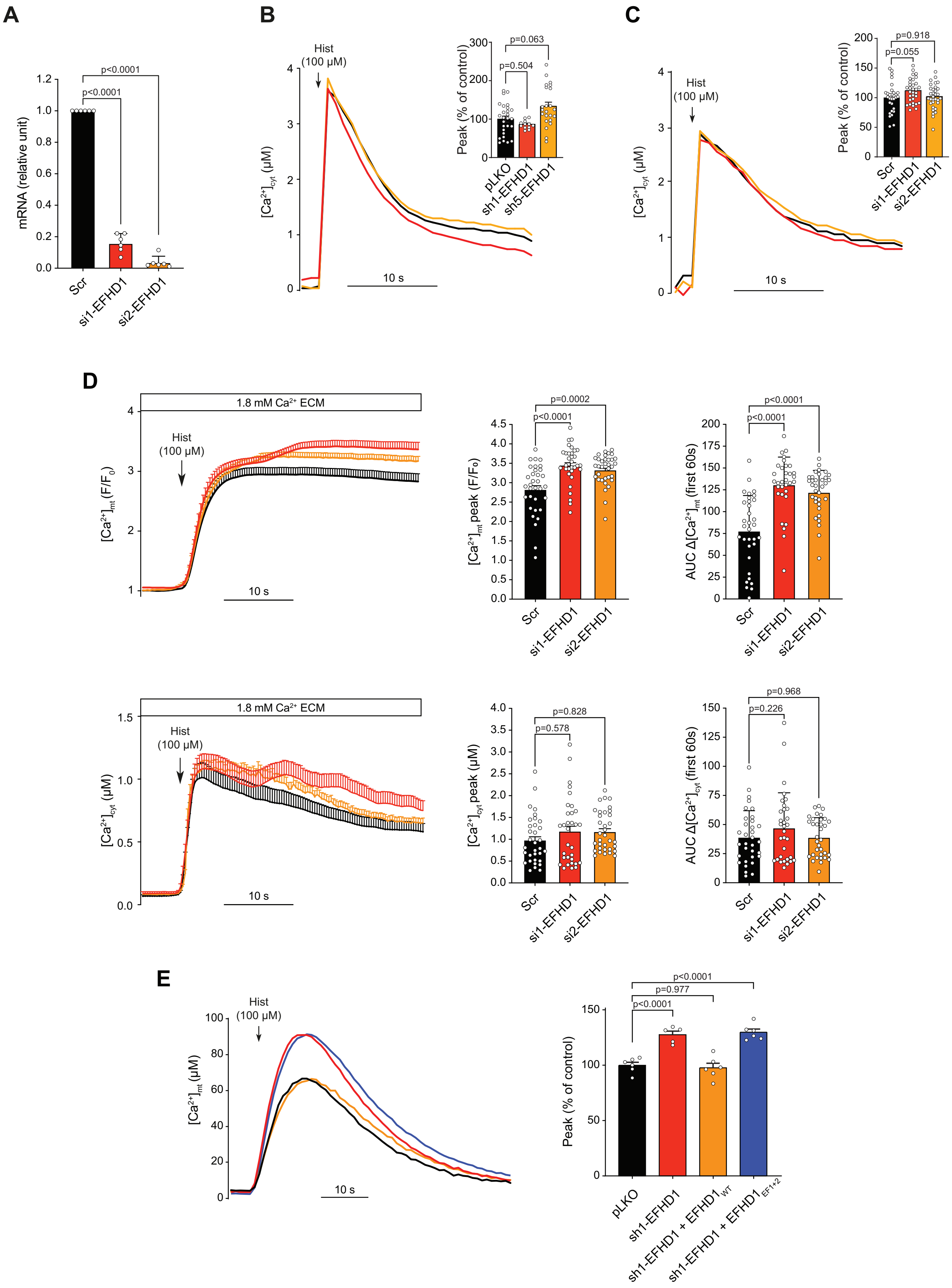
A
p<0.0001
p<0.0001
mRNA (relative unit)
Scr
si1-EFHD1
si2-EFHD1
B
Hist
(100 µM)
$[Ca^{2+}]_{cyt}$ (µM)
10 s
p=0.063
p=0.504
Peak (% of control)
pLKO
sh1-EFHD1
sh5-EFHD1
C
Hist
(100 µM)
$[Ca^{2+}]_{cyt}$ (µM)
10 s
p=0.918
p=0.055
Peak (% of control)
Scr
si1-EFHD1
si2-EFHD1
D
1.8 mM Ca²⁺ ECM
Hist
(100 µM)
$[Ca^{2+}]_{mt}$ (F/F$_0$)
10 s
p=0.0002
p<0.0001
$[Ca^{2+}]_{mt}$ peak (F/F₀)
p<0.0001
p<0.0001
AUC Δ$[Ca^{2+}]_{mt}$ (first 60s)
Scr
si1-EFHD1
si2-EFHD1
1.8 mM Ca²⁺ ECM
Hist
(100 µM)
$[Ca^{2+}]_{cyt}$ (µM)
10 s
p=0.828
p=0.578
$[Ca^{2+}]_{cyt}$ peak (µM)
p=0.968
p=0.226
AUC Δ$[Ca^{2+}]_{cyt}$ (first 60s)
Scr
si1-EFHD1
si2-EFHD1
E
Hist
(100 µM)
$[Ca^{2+}]_{mt}$ (µM)
10 s
p<0.0001
p=0.977
p<0.0001
Peak (% of control)
pLKO
sh1-EFHD1
sh1-EFHD1 + EFHD1$_{WT}$
sh1-EFHD1 + EFHD1$_{EF1+2}$

Figure EV3. EFHD1 inhibits MCU-dependent uptake of Ca^{2+} in mitochondria without affecting $[Ca^{2+}]_{cyt}$ transients.

(**A**) Quantification of EFHD1 KD by real-time PCR (mean ± SEM; $n = 6$ biological replicates); one-way ANOVA with Dunnett's multiple comparisons test. (**B**, **C**) Representative traces and quantification of $[Ca^{2+}]_{cyt}$ transients upon histamine (Hist) stimulation in (**B**) sh-EFHD1 and (**C**) si-EFHD1 HeLa cells expressing cytosolic aequorin (mean ± SEM; $n \geq 12$ biological replicates); one-way ANOVA with Dunnett's multiple comparisons test. (**D**) Quantification of $[Ca^{2+}]_{mt}$ (upper panel) and $[Ca^{2+}]_{cyt}$ (lower panel) responses in control (Scr) and si-EFHD1 treated HeLa cells upon histamine-induced ER Ca^{2+} release in presence of 1.8 mM Ca^{2+} in the extracellular medium (ECM). Peak and area under the curve (AUC) are calculated for the first 60 s of histamine (Hist) stimulation (mean ± SEM from 3 independent experiments ($n \geq 30$ cells from 2 independent experiments); Student's *t* test. (**E**) Representative traces and quantification of $[Ca^{2+}]_{mt}$ transients upon histamine (Hist) stimulation in HeLa cells either expressing EFHD1-targeting shRNA alone or with EFHD1 expression rescue using the $EFHD1_{WT}$ and $EFHD1_{EF1+2}$ constructs (mean ± SEM; $n \geq 5$ biological replicates); one-way ANOVA with Dunnett's multiple comparisons test. Refer to Fig. 4.

© The Author(s)

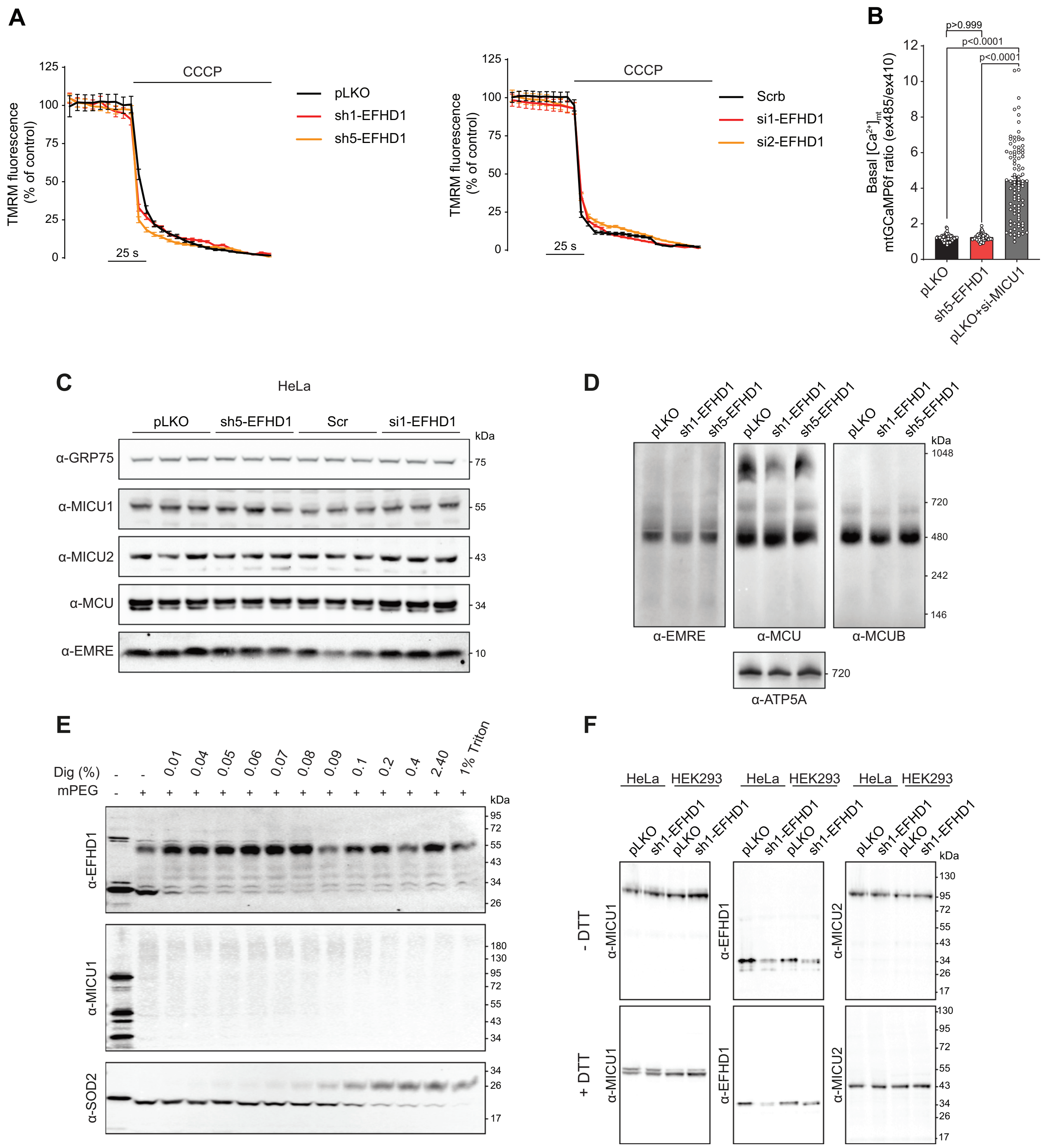

A
TMRM fluorescence (% of control)
CCCP
25 s
pLKO
sh1-EFHD1
sh5-EFHD1
Scrb
si1-EFHD1
si2-EFHD1
B
Basal [Ca2+]mt mtGCaMP6f ratio (ex485/ex410)
p>0.999
p<0.0001
p<0.0001
pLKO
sh5-EFHD1
pLKO+si-MICU1
C
HeLa
pLKO
sh5-EFHD1
Scr
si1-EFHD1
kDa
α-GRP75
α-MICU1
α-MICU2
α-MCU
α-EMRE
D
α-EMRE
α-MCU
α-MCUB
α-ATP5A
E
Dig (%)
mPEG
1% Triton
α-EFHD1
α-MICU1
α-SOD2
F
HeLa
HEK293
- DTT
+ DTT
α-MICU1
α-EFHD1
α-MICU2

Figure EV4. Effect of EFHD1 knockdown on mitochondrial membrane potential, basal Ca^{2+} level, and MCUC assembly.

(**A**) TMRM fluorescence in HeLa cells upon stable (sh-EFHD1) or transient (si-EFHD1) EFHD1 silencing. Data are expressed as a percentage of the control (mean ± SEM; n ≥ 53 cells). (**B**) Resting $[Ca^{2+}]_{mt}$ in HeLa cells upon stable EFHD1 silencing. Data are expressed as the ratio between mt-GCaMP6f fluorescence upon excitation at 485 and 410 nm (mean ± SEM; *n* ≥ 59 cells); one-way ANOVA with Tukey's multiple comparisons test. si-MICU1 is used as positive control. (**C**) Immunoblot analysis of MCUC protein level in whole cell lysates from HeLa cells upon stable (sh-EFHD1) or transient (si-EFHD1) EFHD1 silencing. GRP75 is used as a loading control. (**D**) BN-PAGE analysis of MCUC assembly in mitochondria isolated from sh-EFHD1 HeLa cells. ATP5A is used as a loading control. (**E**) Immunoblot analysis of EFHD1 in isolated mitochondria from HeLa cells treated with increasing concentrations of digitonin and maleimide functionalized polyethylene glycol (mPEG). MICU1 and SOD2 are used as positive controls for IMS and matrix proteins, respectively. (**F**) Immunoblot analysis of mitochondria from HeLa and HEK293 cells expressing either an empty vector (pLKO) or shRNA against EFHD1. Samples were analyzed in reducing (+DTT, dithiotreitol) and non-reducing (-DTT) conditions to detect disulfide-mediated oligomerization. Refer to Fig. 5.

 © The Author(s)

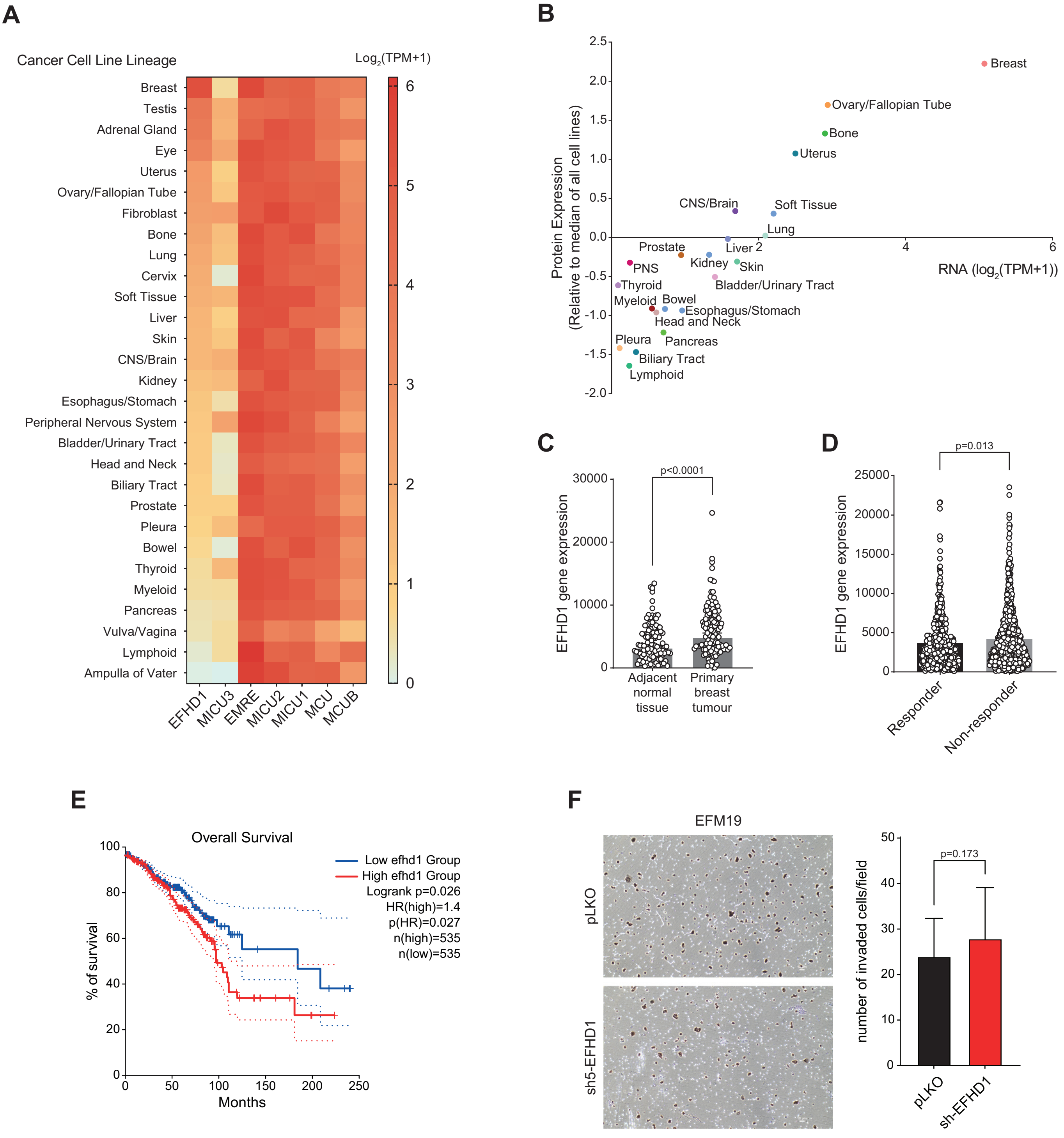
A
Cancer Cell Line Lineage
Log2(TPM+1)
Breast
Testis
Adrenal Gland
Eye
Uterus
Ovary/Fallopian Tube
Fibroblast
Bone
Lung
Cervix
Soft Tissue
Liver
Skin
CNS/Brain
Kidney
Esophagus/Stomach
Peripheral Nervous System
Bladder/Urinary Tract
Head and Neck
Biliary Tract
Prostate
Pleura
Bowel
Thyroid
Myeloid
Pancreas
Vulva/Vagina
Lymphoid
Ampulla of Vater
EFHD1
MICU3
EMRE
MICU2
MICU1
MCU
MCUB
B
Protein Expression
(Relative to median of all cell lines)
RNA (log2(TPM+1))
Breast
Ovary/Fallopian Tube
Bone
Uterus
CNS/Brain
Soft Tissue
Lung
Prostate
Liver
Kidney
Skin
PNS
Thyroid
Bladder/Urinary Tract
Myeloid
Bowel
Esophagus/Stomach
Head and Neck
Pleura
Pancreas
Biliary Tract
Lymphoid
C
EFHD1 gene expression
p<0.0001
Adjacent normal tissue
Primary breast tumour
D
EFHD1 gene expression
p=0.013
Responder
Non-responder
E
Overall Survival
Low efhd1 Group
High efhd1 Group
Logrank p=0.026
HR(high)=1.4
p(HR)=0.027
n(high)=535
n(low)=535
% of survival
Months
F
EFM19
pLKO
sh5-EFHD1
number of invaded cells/field
p=0.173
pLKO
sh-EFHD1

Figure EV5. Assessment of EFHD1 as a potential target in cancer.

(**A**) Heatmap of gene expression level (TPM, transcripts per million) for the known MCUC components and EFHD1 retrieved from the DepMap Public 23Q2 release (Ghandi et al, 2019) and grouped according to cell line lineage ($n = 1450$ cell lines; 29 lineages). Averaged values are inferred from RNA-sequencing data using the RSEM tool and $\log_2$ transformed, using a pseudo-count of 1 ($\log_2(\text{TPM} + 1)$). (**B**) Correlation between protein and RNA levels of EFHD1 in 1019 different cancer cell lines, grouped based on cell lineage average expression. RNA-sequencing data were retrieved from DepMap Public 23Q2 release (Ghandi et al, 2019) whereas normalized protein expression data were taken from (Nusinow et al, 2020); RNA-protein expression Pearson correlation $r^2 = 0.85$. (**C**) Median EFHD1 expression in pairs of primary breast tumor and their adjacent normal tissue ($n = 112$ paired samples). Data were extracted from TNMplot.com median ± 95% confidence interval (CI) and Wilcoxon match-paired two-tailed test. (**D**) Median EFHD1 expression in neoadjuvant chemotherapy in responder ($n = 532$ independent samples) and non-responder ($n = 1100$ independent samples) breast cancer patients analyzed using ROCplot.org from GEO/Array express data median ± 95% CI; Mann–Whitney two-tailed test. (**E**) Survival of breast cancer patients exhibiting high and low EFHD1 expression. Data were retrieved from TCGA-BRCA; Kaplan–Meier plot and the median expression was used as the cohort cutoff, respectively. HR hazard ratio. Dotted lines indicate 95% confidence interval (CI 95%). (**F**) Boyden Chamber migration assay on EFM19 pLKO and sh-EFHD1 cells. Representative image (left) and quantification of migrated cells per field (right). Mean ± SEM from 3 independent experiments ($n \geq 30$ biological replicates); Student's *t* test. Refer to Fig. 5.

 © The Author(s)

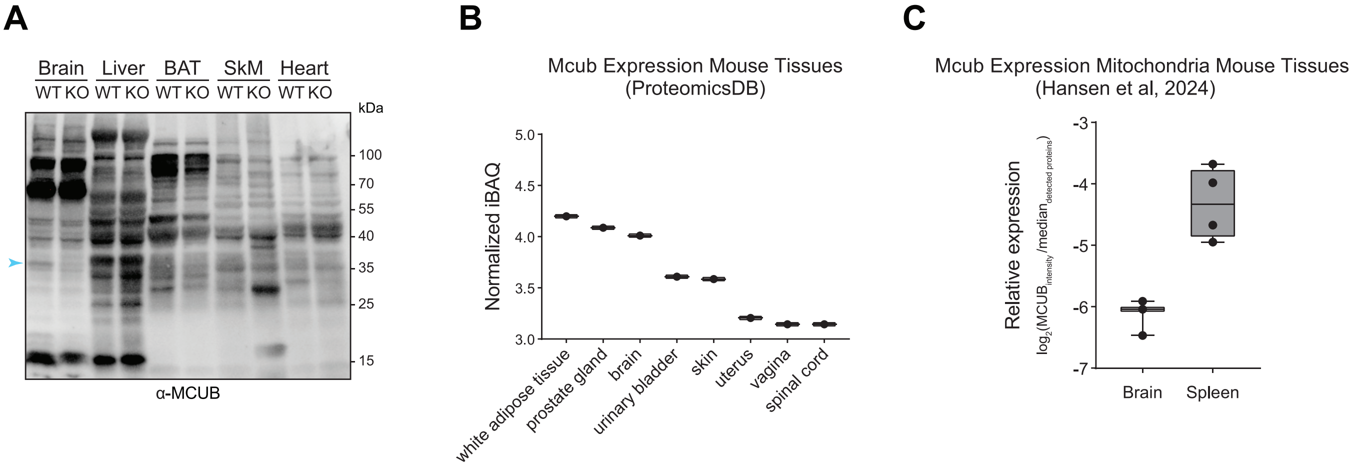

Figure EV6. Validation of MCUB as an inhibitor of MCU protein-protein interactions.

(**A**) Immunoblot analysis of MCUB in whole tissue lysates from MCUB KO and wild-type mice. Blue arrow indicates MCUB protein (BAT, brown adipose tissue; SkM, skeletal muscle). (**B**) Relative MCUB protein expression in mouse tissues from the ProteomicsDB database (white adipose tissue $n = 1$, prostate gland $n = 1$, brain $n = 4$, skin $n = 1$, uterus $n = 1$, vagina $n = 1$, urinary bladder $n = 1$, spinal cord $n = 1$). The line in the middle of the box is plotted at the mean normalized iBAQ, the boxes extend from the minimum normalized iBAQ to the maximum normalized iBAQ values. (**C**) Relative MCUB protein expression in pure mitochondrial proteomes extracted from Hansen et al, 2024. MCUB was detected only in spleen ($n = 4$ biological replicates) and brain ($n = 3$ biological replicates). The line in the middle of the box is plotted at the median, the boxes extend from the 25th to the 75th percentile, and the whiskers extend to the minimum and maximum values. Refer to Fig. 6.
